# OICR-41103 as a chemical probe for the DCAF1 WD40 domain

**DOI:** 10.1038/s42003-025-08491-0

**Published:** 2025-07-19

**Authors:** Serah W. Kimani, Mahmoud Noureldin, Brian Wilson, Laurent Hoffer, Stuart R. Green, Magdalena M. Szewczyk, Héctor González-Álvarez, Mohammed Mohammed, Manuel Chan, Chiara Krausser, Alice Shi Ming Li, Taraneh Hajian, Sarah Tucker, Dhananjay Joshi, Punit Saraon, Brigitte Thériault, Ji Sup Kim, Vijayaratnam Santhakumar, Peter Loppnau, Yanjun Li, Almagul Seitova, Aiping Dong, Taira Kiyota, Tobias Hammann, Paul Gehrtz, Bhashant Patel, Vaibhavi Rathod, Anand Vala, Bhimsen Rout, Paras Jagodra, Peter J. Brown, Ahmed Aman, Jailall Ramnauth, Gennady Poda, David Uehling, Cheryl H. Arrowsmith, Dalia Barsyte-Lovejoy, Richard Marcellus, Suzanne Ackloo, Ahmed Mamai, Rima Al-awar, Levon Halabelian

**Affiliations:** 1 Structural Genomics Consortium, University of Toronto, Toronto, ON Canada; 2 Drug Discovery Program, Ontario Institute for Cancer Research, Toronto, ON Canada; 3 Department of Pharmacology and Toxicology, University of Toronto, Toronto, ON Canada; 4 Medicinal Chemistry, Global Research & Development, Merck Healthcare KGaA, Darmstadt, Germany; 5 Piramal Discovery Solutions, Pharmaceutical Special Economic Zone, Ahmedabad, Gujarat India; 6 Structural Genomics Consortium, Eshelman School of Pharmacy, University of North Carolina at Chapel Hill, Chapel Hill, North Carolina USA; 7 Leslie Dan Faculty of Pharmacy, University of Toronto, Toronto, ON Canada; 8 Princess Margaret Cancer Centre, University Health Network, Toronto, ON Canada; 9 Department of Medical Biophysics, University of Toronto, Toronto, ON Canada; 10 Department of Chemistry, University of Toronto, Toronto, ON Canada

**Keywords:** Small molecules, Ubiquitylation

## Abstract

Human DCAF1 is a multidomain protein that plays a critical role in protein homeostasis. Its WDR domain functions as a substrate recruitment module for RING-type CRL4 and HECT family EDVP E3 ubiquitin ligases, enabling the ubiquitination and proteasomal degradation of specific substrates. DCAF1’s activity has been implicated in cell proliferation and is documented to promote tumorigenesis. Additionally, the DCAF1 WDR domain is hijacked by lentiviral accessory proteins to induce the degradation of host antiviral factors, such as SAMHD1 and UNG2. These diverse roles make DCAF1 an attractive target for therapeutic development in oncology and antiviral strategies. It is also a promising candidate for use in targeted protein degradation. We previously reported a novel ligand, OICR-8268, that targets the DCAF1 WDR domain. In this study, we present the development of OICR-41103, a potent, selective, and cell-active small molecule chemical probe for DCAF1, derived from OICR-8268. The co-crystal structure of the DCAF1-OICR-41103 complex reveals the ligand’s binding mode within the WDR central pocket, demonstrating its potential for PROTAC design and development. Notably, OICR-41103 effectively displaces the lentiviral Vpr protein from DCAF1 in both biochemical and cellular settings, highlighting its potential for the development of HIV therapeutics.

## Abstract (web summary)

OICR-41103 is a potent, selective probe targeting the DCAF1 WDR domain and displacing viral Vpr protein. It enables new opportunities in cancer research, antiviral therapy, and targeted protein degradation via PROTACs.

## Introduction

DCAF1 (DDB1-cullin4-associated-factor 1), also known as the VPR-bindingprotein (VPRBP), is a multifunctional protein that acts as a substrate recognition module in two distinct E3 ligase complexes: the Cullin4-RING E3 ligase (CRL4) and the HECT E3 ligase EDD-Dyrk2-DDB1 (EDVP)^1–3^. As a substrate receptor, DCAF1 facilitates the ubiquitination and subsequent proteasomal degradation of various proteins by mediating the recognition and binding of substrate proteins for transfer of ubiquitin from an E2 enzyme to the substrate protein. DCAF1 is characterized by its multi-domain structure, which includes a 7-bladed WD40 repeat (WDR) domain with a beta-propeller structure, an N-terminal armadillo (ARM) repeat motif spanning amino acids 80-796, the kinase-like and chromo domains within the ARM motif^4^, a central Lis homology (LisH) domain^5^, and an acidic domain in the C-terminus^6^ (Supplementary Fig. 1).

DCAF1 targets substrates for degradation that are involved in diverse cellular processes, including the cell cycle (e.g., Katanin p60, CP110, TERT^1,7,8^), DNA damage response (e.g., MCM10, p53^9–11^), and gametogenesis (e.g., TET, PP2A^12–14^). In the immune system, DCAF1 plays a role in promoting T-cell receptor activation and B-cell maturation^11,15^. DCAF1 negatively regulates TGF-β signaling by stabilizing Smurf1^16^. DCAF1 also inhibits microRNA biogenesis by controlling DICER levels^17^, influences muscle differentiation by degrading MyoD^18^, affects lipid metabolism through TR4 ubiquitination and degradation^19^, and plays a role in ribosomal biogenesis by regulating PWP1 levels^20^. Notably, DCAF1 also exhibits E3 ligase-independent functions, such as phosphorylating H2A^4^ (H2AT120p), p53^21^ (S367p), and EZH2^22^ (T367), and it dissociates from the E3 ligase complex during the G_2_/M phase to enhance FoxM1 activity and mitosis^23^. Additionally, DCAF1 binds to un-acetylated histone 3 (H3), thereby repressing p53-mediated transcription^21^.

Originally identified for its interaction with the HIV protein R (Vpr), DCAF1 is hijacked by primate lentiviruses via Vpr/Vpx to exploit the CRL4^DDB1-DCAF1^ E3 ligase complex. This interaction induces G_2_/M arrest and promotes the degradation of cellular antiviral factors, thus aiding viral replication and immune evasion. Vpr targets various DNA damage proteins (e.g., MUS81, HLTF, UNG2^24–31^) and epigenetic silencing proteins (e.g., HUSH complex, CTIP2, NuRD^32–35^), which are critical for viral replication. Understanding these interactions is crucial for advancing HIV treatment strategies.

DCAF1 is also implicated in tumor development, with overexpression observed in lung, breast, bladder, high-grade serous ovarian, and prostate cancers^4,23,36,37^. Its overexpression is associated with poor prognosis in lung adenocarcinoma, suggesting DCAF1 as a potential therapeutic target^37^. DCAF1 regulates known tumor suppressors, such as Lgl1 and p53, and contributes to tumorigenesis by inactivating the growth-suppressive Hippo pathway via ubiquitination of Lats1/2 kinases^11,21,36,38–41^. DCAF1 also modulates the oncogenic transcription factor FoxM1 in a cell cycle-specific manner, through both degradation and transcriptional activation^23^. These findings highlight DCAF1’s multiple roles in tumor progression and the utility of developing a high quality chemical probes for the DCAF1 WD40 domain to further characterize its role in cancer pathogenesis

**Fig. 1:**
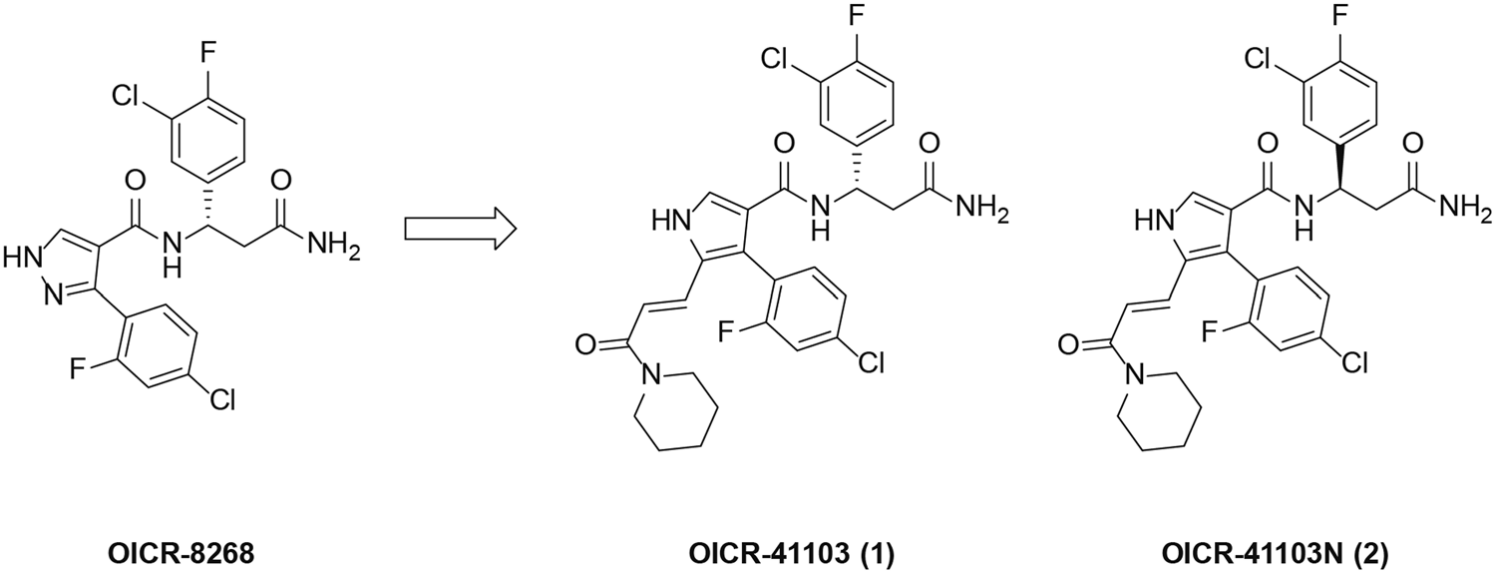
Development of pyrroles OICR-41103 and OICR-41103N from pyrazole OICR-8268. OICR-8268 is a previously reported DCAF1 ligand. OICR-41103 (1) represents the active chemical probe, while OICR-41103N serves as the corresponding negative control.

Previously, we reported OICR-8268, a novel small molecule binder targeting the WD40 domain of DCAF1, demonstrating in-cell target engagement at ~10 μM^42^. Our efforts to exploit this discovery as a method for hijacking DCAF1’s role as an E3 ligase has not only led us to develop functional PROTACs^43^, but also generated small molecule binders of DCAF1 that can be used as chemical probes to further interrogate its cellular function. The key replacement of the core pyrazole of OICR-8268 with a pyrrole enabled the synthesis of analogs that could target the solvent exposed region of the WD domain. Of the 2-substituted pyrroles synthesized, OICR-41103 stood out for its nanomolar biochemical activity and dramatically improved activity in cellular assays. We present here OICR-41103 as the small molecule chemical probe of the WD40 domain of DCAF1 (Fig. 1). The (R) enantiomer of OICR-41103, designated as OICR-41103N, provides a convenient negative control to be used alongside the active (S) enantiomer.

## Results

### OICR-41103 is a potent binder of the human DCAF1 WDR domain

**Fig. 2:**
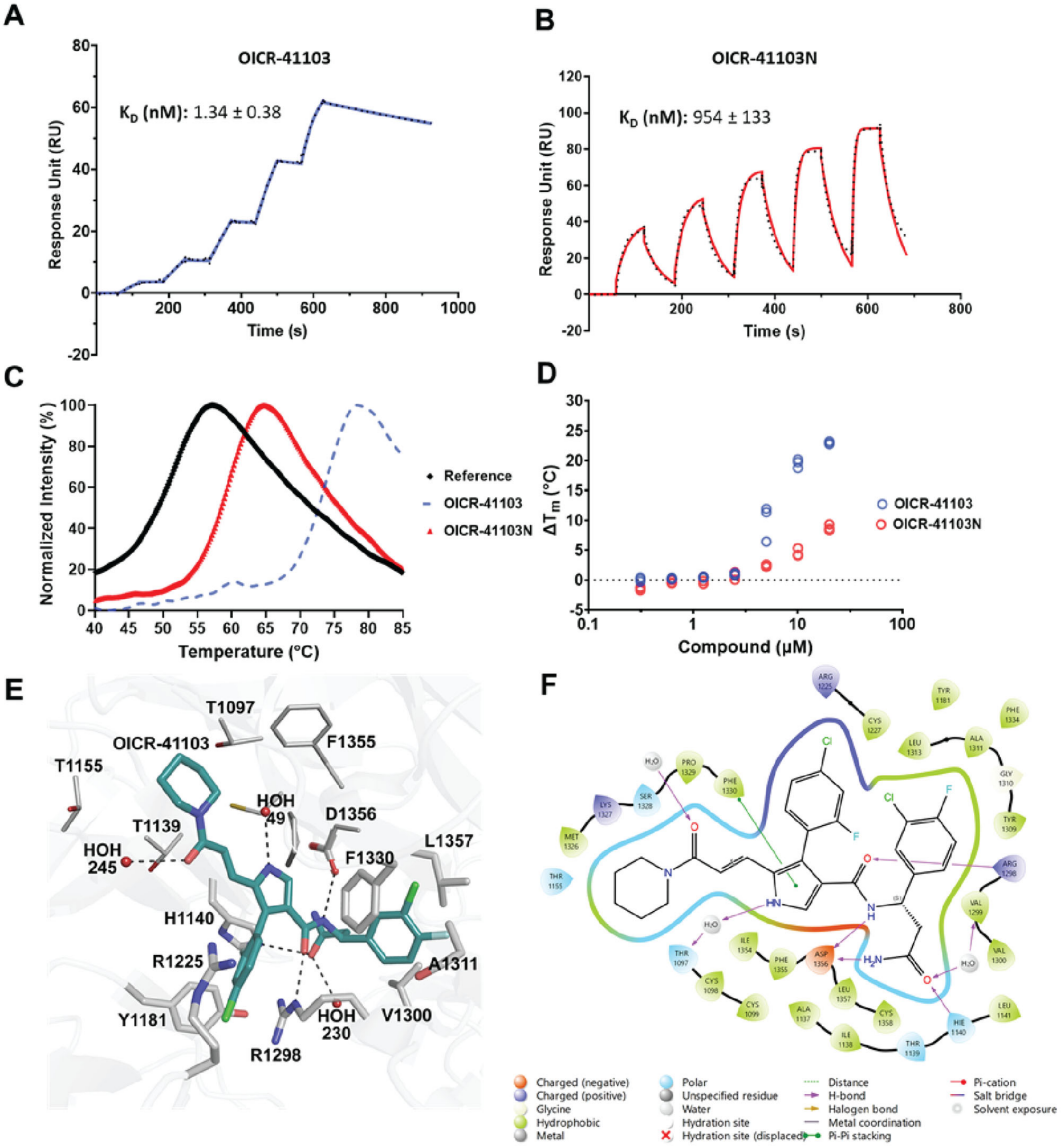
Binding of OICR-41103 and OICR-41103N to the DCAF1 WDR domain. Characterization of OICR-41103 (**A**) and OICR-41103N (**B**) by SPR. **C** Thermal stabilization of. DCAF1 WDR domain at 20 µM by OICR-41103 with a ΔTm of 23.0 ± 0.1 °C (blue dashed trace), and OICR-41103N with a ΔT_m_ of 8.8 ± 0.3 °C (red trace) compared to the DCAF1 WDR domain with no compound (black trace), as measured by DSF. **D** The WDR domain stabilization was concentration dependent. **E** A close-up view of the OICR-41103 (deep teal sticks) binding site within the DCAF1 WDR central pocket. Coordinating water molecules are shown as red spheres. Residues interacting with the ligands are rendered as thin sticks, and hydrogen bonds are shown in black dashes. **F** A 2D diagram of the protein-compound interactions. The most important interaction classes, including water-mediated hydrogen bonds, are represented. All protein residues within 5.0 Å from ligand are displayed. The picture was generated using the LID tool from Maestro software (Schrodinger) from the prepared X-ray structure (PDB: 9D4E).

Building on our previously reported DCAF1 ligand, OICR-8268 (PDB ID: 8F8E)^42^, we sought to enhance ligand binding by extending it toward the exposed solvent front. To achieve this, we replaced the core heterocycle from a pyrazole to a 2-subsituted pyrrole (Fig. 1). This structural modification led to significant improvements in both biochemical and cellular activity. The binding affinities of OICR-41103 and its opposite enantiomer, OICR-41103N, to the DCAF1 WDR domain were initially evaluated with SPR. OICR-41103 exhibited potent binding to DCAF1 with a *K*_D_ value below 2 nM, whereas OICR-41103N showed significantly weaker binding, with a *K*_D_ value of approximately 1 µM (Fig. 2A, B).

To confirm these binding interactions, differential scanning fluorimetry (DSF) was employed as an orthogonal method to assess the compounds’ ability to thermally stabilize the DCAF1 WDR domain. OICR-41103 significantly stabilized DCAF1, with a maximum ΔT_m_ of 23.0 ± 0.1 °C at 20 µM. In contrast, OICR-41103N demonstrated a much weaker stabilization effect, with a ΔT_m_ of 8.8 ± 0.3 °C at the same concentration (Fig. 2C). Moreover, the stabilization effect of OICR-41103 increased dramatically with increasing concentrations compared to OICR-41103N (Fig. 2D), highlighting its superior binding efficacy.

### OICR-41103 is selective for DCAF1 WDR domain

The selectivity of OICR-41103 and its negative enantiomer, OICR-41103N, for DCAF1 was assessed using SPR against a panel of six WDR proteins. OICR-41103 showed no significant binding to any of the WDR proteins in the panel, even at concentrations up to 20 µM (Supplementary Fig. 2A). As expected, the DCAF1 control in this experiment yielded a *K*_D_ consistent with its previously determined SPR *K*_D_ value (~1 nM) (Fig. 2A). In contrast, the negative control enantiomer, OICR-41103N, displayed weaker binding to DCAF1 (*K*_D_ = 1.5 µM) and no binding to any of the WDR proteins in the selectivity panel, except for WDR5. OICR-41103N exhibited moderate binding to WDR5 with a *K*_D_ of 1 µM (Supplementary Fig. 2B). However, further investigation revealed that the observed binding activity to WDR5 was likely due to an impurity, which was successfully removed during re-purification of OICR-41103N (Supplementary Fig. 3).

### The co-crystal structure of the DCAF1 WDR domain in complex with OICR-41103

To elucidate the compound binding mode, we co-crystallized the DCAF1 WDR domain (residues 1077–1390) with OICR-41103, referred to here as DCAF1-OICR-41103. The structure was solved in the P1211 space group with two molecules in the crystal asymmetric unit and was refined to 1.7 Å resolution. Table 1 summarizes the crystallographic data collection, structure refinement and validation statistics.

**Table. 1:** Diffraction data collection, phasing and refinement statistics

DCAF1-OICR-41103	
Data collection	
Space group	P12_1_1 Cell dimensions
*a*, *b*, *c* (Å)	48.574, 87.975, 73.136
α, β, γ (°)	90.00, 96.94, 90.00
Resolution (Å)	50.0–1.70 (1.73–1.70)^a^
*R*_sym_ or *R*_merge_	0.051 (0.981)
I/σ	28.11 (1.72)
Completeness (%)	99.8 (99.1)
Redundancy	6.4 (6.0)
Refinement	
Resolution (Å)	1.70
No. reflections	63834
^*R*^work^/*R*^free	
17.84/20.63	
No. atoms	
Protein	4754
Ligand/ion	91
Water	304
*B*-factors	
Protein	33.87
Ligand/ion	30.14
Water	36.70
R.m.s. deviations	
Bond lengths (Å)	0.0046
Bond angles (°)	1.33

^a^Values in parentheses are for the highest resolution shell.

Clear electron density was observed for the entire OICR-41103 molecule in both chains of the crystal asymmetric unit (Supplementary Fig. 4). As expected, OICR-41103 binds within the central pocket of the DCAF1 WDR domain. However, unlike the parent molecule (OICR-8268)^42^, OICR-41103 extends further out of the central pore towards the surface of the WDR domain (Supplementary Fig. 5). OICR-41103 engages in extensive interactions with residues lining the central pocket of the DCAF1 WDR domain, as well as the surrounding water molecules (Fig. 2E, F). The northern di-halogenated phenyl ring binds deep within a hydrophobic pocket formed by residues V1299, V1300, Y1309, G1310, A1311, F1330, I1354, and L1357 (Fig. 2E, F). Similarly, the southern di-halogenated aromatic ring occupies another hydrophobic pocket, surrounded by H1140, Y1181, C1227, L1313, P1329, and F1330. In addition to these hydrophobic interactions, the side chain of R1298 mediates cation-π interactions with the southern ring.

The central amide group of OICR-41103 forms hydrogen bonds with the side chain of D1356 on one side and with the guanidinium group of R1298 on the other. The pyrrole ring, which replaces the pyrazole moiety in the parent compound OICR-8268 (Supplementary Fig. 6A, B), engages in an edge-to-face aromatic interaction with the side chain of F1330 and forms a hydrogen bond with a nearby water molecule. Additional vinyl amide and piperidine groups extend toward the surface of the WDR domain, occupying a broader section of the central pocket. These groups establish hydrophobic and Van der Waals interactions with T1097, C1098, T1139, T1155, P1329, F1355 and I1369. The vinyl amide group is further stabilized by a hydrogen bond to a water molecule (Fig. 2E, F).

### OICR-41103 binds and engages the WDR domain of DCAF1 in cells

The Cellular Thermal Shift Assay (CETSA) was used to evaluate the engagement of OICR-41103 with the WDR domain (1038-1400aa) of DCAF1 in NCI-H460 cells stably expressing the HiBiT-tagged WDR domain of DCAF1. In this assay, the thermal stabilization of the protein indicates compound binding.

**Fig. 3:**
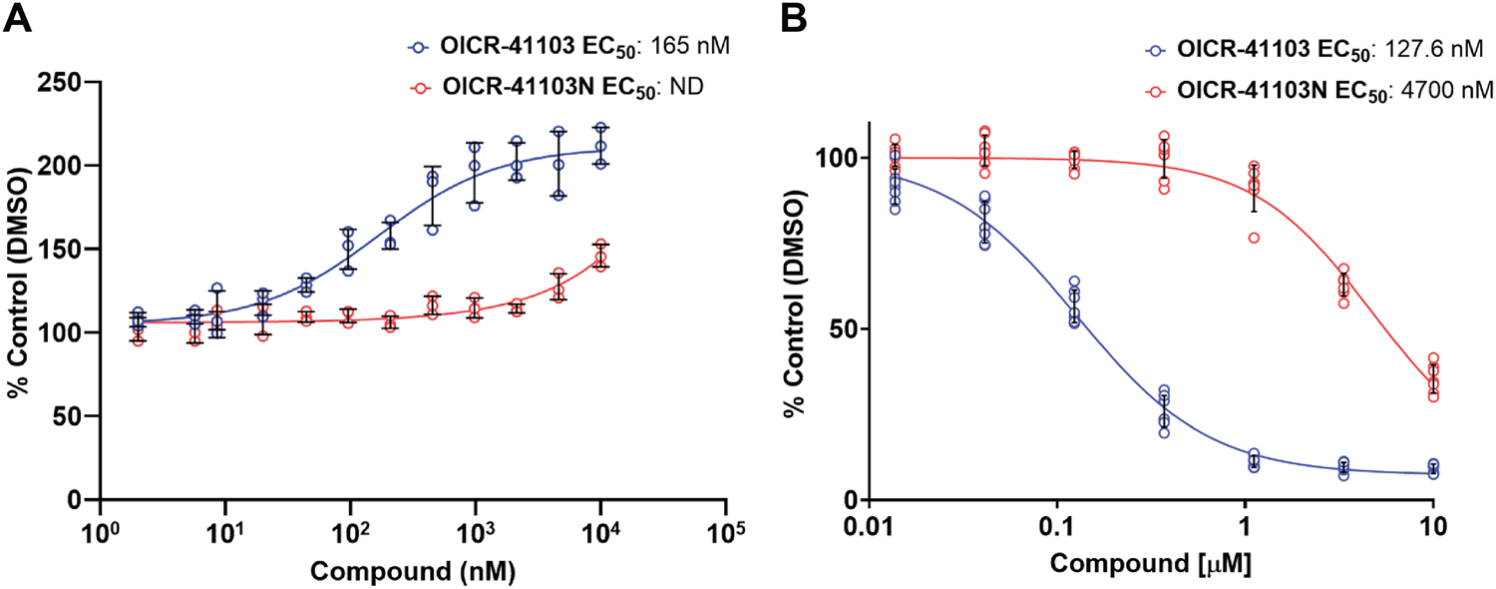
OICR-41103 engages and binds to the WDR domain of DCAF1 in cells. **A** Cellular Thermal Shift Assay (CETSA) was used to assess OICR-41103 binding to the WDR domain of DCAF1 in NCI-H460 cells expressing HiBiT-tagged WDR domain of DCAF1. OICR-41103 thermally stabilized the WDR domain of DCAF1 in a dose dependent manner with an EC_50_ of 167 nM. Results are shown as an average +/−SD (*n* = 3 biologically independent samples). **B** NanoBRET assay with fluorescently labeled DCAF1 tracer was used to confirm cellular target engagement. OICR-41103 decreases the NanoBRET ratio between DCAF1 tracer and N-terminally NL-tagged DCAF1(WD40) in a dose-dependent manner. The negative control compound OICR-41103N was over 35-fold less potent. HEK 293T cells were transfected with N-terminally NL-tagged DCAF1(WD40) for 24 h and treated with tracer in presence or absence of compounds for 1 h. Results are shown as an average +/−SD (*n* = 7, 2 biologically independent samples).

As shown in Fig. 3A, OICR-41103 effectively thermally stabilized the WDR domain of DCAF1 in a dose-dependent manner, with an EC_50_ of 165 nM. This stabilization indicates that OICR-41103 binds specifically to the WDR domain, protecting it from heat-induced denaturation. In contrast, the enantiomer negative control, OICR-41103N, did not show any significant thermal stabilization of the WDR domain, confirming the specificity of OICR-41103 stabilization. These results show that OICR-41103 specifically engages and stabilizes the WDR domain of DCAF1 in cells.

To confirm target engagement of OICR-41103 in cells, we utilized Bioluminescence Resonance Energy Transfer (BRET) technology, which measures energy transfer from exogenously expressed NanoLuc® luciferase (NL)-tagged DCAF1-WDR and a cell-permeable DCAF1 fluorescent tracer that reversibly binds DCAF1-WDR. The Pyrrolo-BODIPY tracer was developed by structural analogy to the BODIPY-containing tracers disclosed by Vulpetti^44^ and Schröder^45^. Extending the linker to PEG3 and inclusion of the potency-increasing spiro-cyclohexyl subunit compared to the starting point led to a permeable tracer allowing displacement studies. Cell treatment with OICR-41103 resulted in a dose dependent loss of the NanoBRET signal indicating that the compound displaced the tracer from DCAF1-WDR confirming binding specificity in a cellular environment. OICR-41103 potently displaced tracer with an EC_50_ of 128 nM, whereas its negative control compound, OICR-41103N, was over 35 times weaker with an EC_50_ of 4.7 µM (Fig. 3B).

### OICR-41103 displaces viral accessory protein Vpr from the DCAF1 WDR domain

**Fig. 4:**
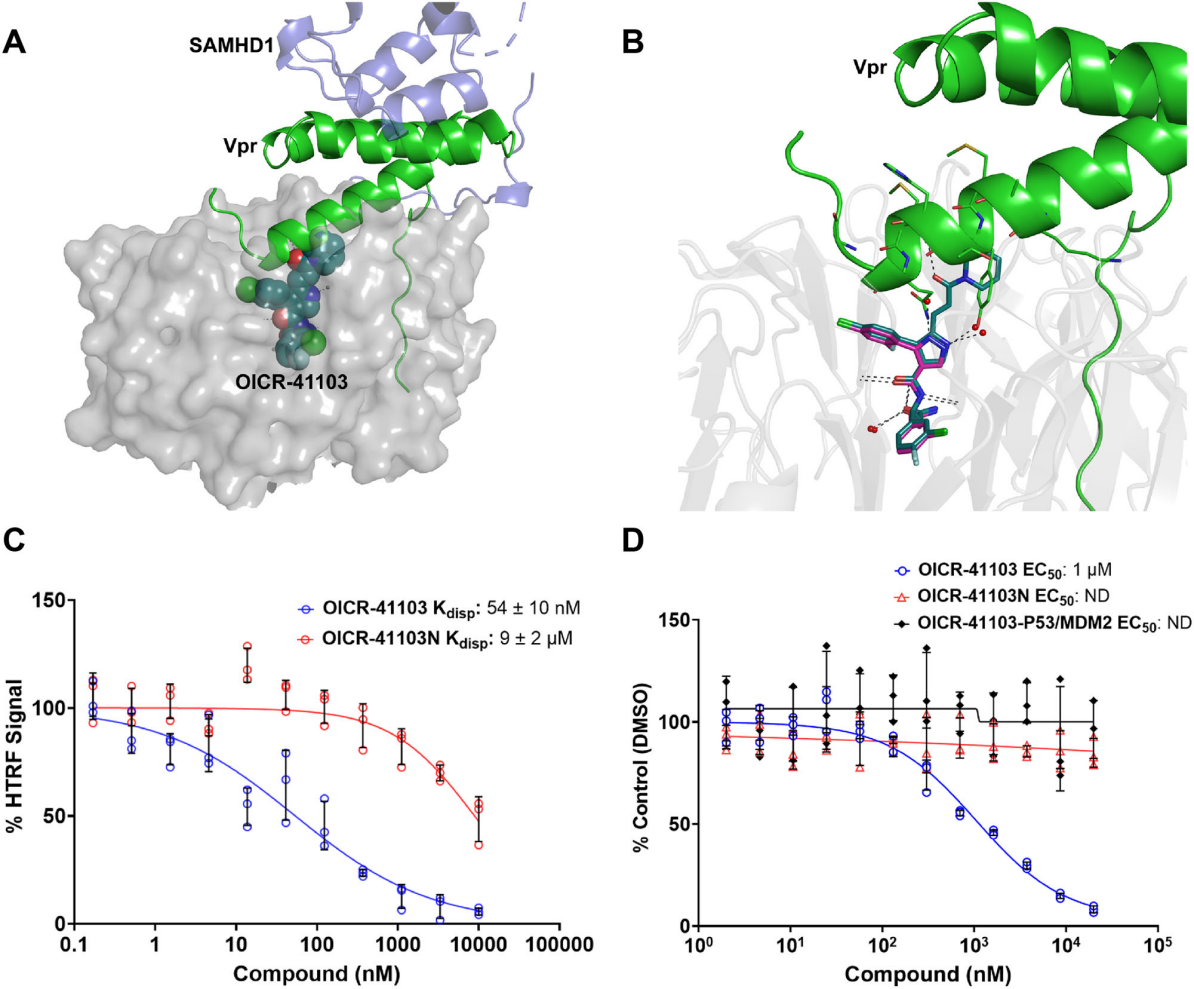
Displacement of Vpr by OICR-41103. **A** A transparent surface representation of the DCAF1 WDR domain with the bound OICR-41103 ligand (deep teal spheres), superimposed with the DCAF1-Vpr-SAMHD1 ternary structure (PDB: 5AJA). **B** A close-up view of the OICR-41103 (deep teal sticks) binding site, compared to the binding site of the parent compound, OICR-8268, (magenta sticks, PDB: 8F8E) and the Vpr binding site in the DCAF1-Vpr-SAMHD1 structure (PDB: 5AJA). OICR-41103 creates a steric clash with Vpr. **C** An HTRF assay demonstrating that OICR-41103 potently disrupts the interaction of the DCAF1 WDR domain and full-length Vpr protein. OICR-41103 displaces Vpr from DCAF1 with a *K*_disp_ below 100 nM, demonstrating competition for the same binding site as Vpr. The opposite enantiomer, OICR-41103N, was used as a negative control and demonstrates substantially weaker potency (~180 × difference in *K*_disp_) in displacing the interaction, ±SD *n* = 3 incubations performed in separate wells at given compound concentrations. **D** The effect of OICR-41103 on disrupting the WDR-DCAF1/Vpr interaction in cells was assessed using NanoBit complementation assay. HEK293EMT cells were transfected with the WDR-DCAF1 tagged to smBiT and Vpr tagged to LgBiT. OICR-41103 disrupted WDR-DCAF1/Vpr interaction in a dose-dependent manner, with an EC50 of 1 μM, and had no effect on the negative control pair, p53/MDM2 interaction. Results are shown as an average +/−SD (*n* = 3 biologically independent samples).

A structural comparison of the DCAF1-OICR-41103 complex with the previously reported structure of DCAF1 bound to Vpr and a SAMHD1 peptide^46^ (PDB ID: 5AJA) revealed that OICR-41103 reaches the Vpr binding site on the top side of the WDR domain (Fig. 4A, B), suggesting that OICR-41103 may have the potential to disrupt the interaction between the viral Vpr protein and the DCAF1 WDR domain.

To assess the ability of OICR-41103 to disrupt the interaction between full-length Vpr and the DCAF1 WDR domain, a Homogeneous Time-Resolved Fluorescence (HTRF) assay was developed. A cross-titration experiment first determined that the maximal HTRF signal could be obtained at 22 nM for both proteins (Supplementary Fig. 7A). However, for displacement assays, the protein concentrations were reduced to 2.5 nM to minimize protein consumption, as 22 nM was significantly higher than the SPR-derived *K*_D_ (~1 nM) of the probe. To confirm that the HTRF signal was specific to the interaction between DCAF1 and Vpr, both proteins were incubated at their optimal concentrations with equimolar amounts of unrelated decoy proteins tagged with either biotin or 6xHis. These decoys failed to produce any signal significantly above the baseline (wells only containing SA-XL665 and Tb-anti-6xHis) confirming the specificity of the DCAF1-Vpr interaction (Supplementary Fig. 7B).

A displacement assay was then carried out using unbiotinylated Vpr to demonstrate that the interaction between the biotinylated Vpr and 6xHis-tagged DCAF1 was specific and reversible (Supplementary Fig. 7C). While ~70% disruption of the interaction was apparent at 5 μM after one hour, an overnight incubation time was required to observe full displacement of the interaction, indicating that the DCAF1-Vpr interaction is quite stable and has a slow dissociation rate. Consequently, a 24-h incubation period was chosen for all subsequent displacement assays to ensure sufficient time for maximal displacement. Assessment of the previously reported Novartis compound 13,^44^ which was shown to interact with the WDR domain of DCAF1, demonstrated a *K*_disp_ of ~1 µM, with full displacement observed at a higher DMSO concentration (3%) due to solubility limitations at 2% DMSO (Supplementary Fig. 7D). Interestingly, this was comparable to OICR-8268, the parent molecule of OICR-41103, which demonstrated a *K*_disp_ of 620 nM and achieved full displacement despite showing minimal structural overlap with the Vpr protein (Supplementary Fig. 7E). In contrast, the DCAF1 probe (OICR-41103), which binds with higher affinity and directly overlaps with the Vpr protein, demonstrated substantially greater potency in the HTRF displacement assay, with a *K*_disp_ of 54 ± 10 nM (Fig. 4C). In contrast, the negative control (R-enantiomer) exhibited much weaker activity, with a *K*_disp_ of approximately 9 ± 2 µM (Fig. 4C).

To confirm that OICR-41103 disrupts the DCAF1-Vpr interaction in cells, we employed Promega’s NanoBiT assay, based on NanoLuc technology using SmBiT and LgBiT. As shown in Fig. 4D, OICR-41103 effectively disrupted the interaction between the WDR domain of DCAF1 and Vpr in cells with an EC_50_ of 1 µM. The negative control enantiomer, OICR-41103N, did not disrupt this interaction, confirming the specificity of OICR-41103. Furthermore, the unrelated p53/MDM2 interaction was unaffected, further confirming that the observed cellular disruption by OICR-41103 was specific to the DCAF1-Vpr complex. These findings collectively demonstrate that OICR-41103 selectively engages the WDR domain of DCAF1 and effectively disrupts its interaction with Vpr, both in vitro and in a cellular context.

### DCAF1 knockdown results in a growth suppression phenotype in NSCLC cell lines without toxicity from OICR-41103 treatment

**Fig. 5:**
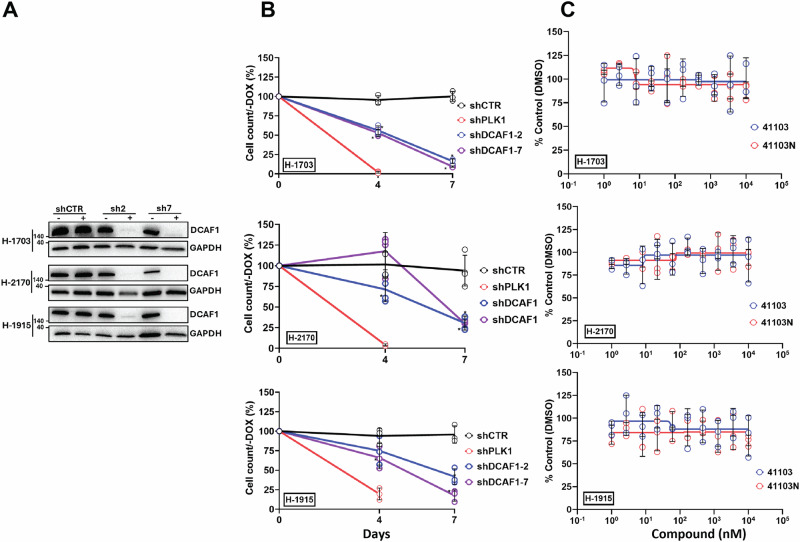
DCAF1 knockdown results in growth suppression of NCIH-1703, NCIH-2170, and NCIH-1915 cell lines, with no toxicity observed upon OICR-41103 treatment. **A** Western-blot analysis showing DCAF1 knockdown after 4 days of doxycycline induction (−/+DOX treatment) in NCI-H1703, NCI-H2170, and NCI-H1915 cells. **B** Trypan blue exclusion assay was used to count the number of cells in -DOX and +DOX treated cells. The percentage of remaining cells was calculated (+DOX/−DOX). shRNAs for Polo-like kinase (PLK1) and a non-targeting control (CTR2) were used as positive and negative growth suppression controls, respectively. **C** ATPlite assay was used to assess the effect of OICR-41103 in NCI-H1703, NCI-H2170, and NCI-H1915 cells after 7 days of compound treatment. Results are shown as an average +/−SD (*n* = 3 biologically independent samples). shDCAF1 cells are compared to shCTR2 cells using unpaired *t*-test, and *P-value < 0.05.

An shRNA doxycycline (DOX) inducible system was used to assess the effect of DCAF1 knockdown on the following NSCLC cell lines: H-1703, H-2170, and H-1915. Stable cell lines expressing two shRNAs against the mRNA of DCAF1 (shDCAF1-2 and shDCAF1-7). DCAF1 knockdown (KD) was confirmed after 4 days of DOX induction for all cell lines (Fig. 5A and Supplementary Fig. 9). Trypan blue exclusion assay showed that DCAF1 KD resulted in a growth suppression phenotype in all cell lines, with a 25% reduction in cell count by day 7 (Fig. 5B). These results suggest that DCAF1 is essential for the growth of the tested cell lines.

Furthermore, OICR-41103 was tested in these cell lines to assess its potential growth suppression effects. However, no growth suppression phenotype was observed up to 7 days of compound treatment, as indicated by the ATP-based viability assays (Fig. 5C). This suggests that OICR-41103 does not exhibit toxicity in the H-1703, H-2170, and H-1915 cell lines. We also tested the effect of OICR-41103 and OICR-41103N on cell growth in 4 additional cell lines HEK293T, MCF7, U2Os and HCT116. Treatment with the compounds for 5 days did not affect cell confluency measured in Incucyte live-cell imager up to 3 µM concentration (Supplementary Fig. 8).

## Discussion

DCAF1 contains a WDR domain and is a member of the WDR protein family, which is one of the most abundant classes of proteins in the human proteome. WDR domains typically adopt a conserved β-propeller fold, which serve as protein interaction scaffolds (Supplementary Fig. 1). By acting as a substrate receptor for both the CRL4^47^ and EDVP^1^ E3 ubiquitin ligase complexes, multiple DCAF1 domains, including the WDR domain, play a critical role in the ubiquitin-proteasome system (UPS). It contains a conserved helix-loop-helix motif for interaction with DDB1 adaptor protein, while also providing a planar, solvent-exposed structure capable of engaging with substrate proteins. Given its central role in recruiting substrates to E3 ligase complexes, the DCAF1 WDR domain has emerged as a promising target for ligand development.

Recent ligands developed for the DCAF1 WDR domain include, OICR-8268, a nanomolar-affinity ligand that binds deep within the central pocket^42^, CYCA-117-113, a double-digit micromolar affinity ligand that binds to the top surface of the WDR central pocket^48^, and a ligand series that binds both the top and side pockets of the WDR domain^44^. Some of these ligands have already been advanced into PROTACs targeting clinically relevant proteins, such as BRD9 and BTK tyrosine kinase^45^.

In this study, we present OICR-41103, a highly potent ligand developed from OICR-8268. OICR-41103 exhibits an exceptional binding affinity for the DCAF1 WDR domain, with an SPR *K*_D_ of ~1 nM (Fig. 2A), making it, to our knowledge, the most potent DCAF1 binder reported to date. It demonstrates strong selectivity for the DCAF1 WDR domain, showing no off-target activity across a panel of six other WDR domain proteins (Supplementary Figs. 2, 3).

We confirmed that OICR-41103 engages the DCAF1 WDR domain in cells. It thermally stabilized the WDR domain in a CETSA assay and displaced a fluorescent tracer in a NanoBret assay, with EC_50_ values of 167 nM and 126.7 nM, respectively (Fig. 3).

Both the CRL4^DCAF1^ and the EDVP^DCAF1^ E3 ligase complexes are hijacked by the primate lentiviral (HIV and SIV) accessory proteins, including the Vpr and Vpx, which bind to the DCAF1 WDR domain to degrade host defense proteins via the proteasome^49,50^. Developing ligands capable of disrupting the DCAF1-Vpr/x interaction is therefore a promising strategy for targeted HIV therapies.

Structural characterization of DCAF1 bound to OICR-41103 revealed that the compound extends to the surface of the WDR domain and occupies a pocket where Vpr peptide binds (Fig. 4A, B). This binding mode makes OICR-41103 a promising candidate for displacement of viral Vpr/x peptides for development of HIV inhibitors. We demonstrate that OICR-41103 potently displaces full-length Vpr from the DCAF1 WDR domain in vitro, with an IC_50_ of 54 ± 10 nM in an HTRF assay (Fig. 4C). In contrast, a previously reported ligand that binds to the top of the central pocket^45^ demonstrated substantially weaker displacement of the same Vpr protein at ~1 µM *K*_disp_ (Supplementary Fig. 7D).

In cells, OICR-41103 effectively disrupts the DCAF1-Vpr interaction, with an EC_50_ of approximately 1 µM determined by a NanoBit assay (Fig. 4D). The negative control compound, OICR-41103N, showed no activity, (Fig. 4D), confirming the specificity of OICR-41103 and highlighting its potential utility as a tool compound for the optimization and development of HIV therapeutics.

DCAF1 is known to regulate several essential cellular processes, including cell cycle regulation, DNA repair, immune function, and tumorigenesis^51,52^. It has also been identified as an essential gene in cancer pathogenesis in the DepMap shRNA and CRISPR screens^53^. However, the molecular mechanisms underlying these functions remain poorly understood. A specific non-toxic ligand that binds the WD domain of DCAF1 without causing off-target effects would be an invaluable tool for advancing our understanding of DCAF1 biology. To assess the cytotoxicity of OICR-41103 and its negative control compound OICR-41103N, we evaluated them in four diverse cell lines (HEK293T, MCF7, HCT116 and U2OS), and observed no effect on cell viability at concentrations up to 3 µM of compound concentration (Supplementary Fig. 8). These findings highlight the potential of utilizing OICR-41103 in probing the biological role of the DCAF1 WDR domain and enabling the development of PROTACs as demonstrated in our recent publication^43^.

To extend our understanding of the overall cellular function and possibly the effect of OICR-41103 on the potential tumorigenesis activity of DCAF1, we compared the effects of DCAF1 knockdown and OICR-41103 treatment in Non-Small Cell Lung Cancer (NSCLC) cells. We observed that DCAF1 knockdown resulted in significant growth suppression, indicating its essential role in cell viability (Fig. 5A, B). However, treatment with OICR-41103, did not produce the same phenotype (Fig. 5C). This difference may arise from the nature or mode of chemical inhibition. While genetic knockdown depletes the entire protein and its associated functions, chemical inhibition with OICR-41103 selectively targets only the WDR domain function, potentially leaving other functional domains of DCAF1 unaffected. Additionally, some DCAF1 substrates may interact transiently or shallowly, making them less susceptible to inhibition by OICR-41103. The functional relevance of targeting the WDR domain may also vary depending on the cancer subtype, warranting further investigation across additional cancer models and disease systems, including HIV, to fully assess its therapeutic potential.

In conclusion, OICR-41103 serves as a powerful tool compound for the interrogation of DCAF1 function in cells. OICR-41103 exhibits high affinity and selectivity for the DCAF1 WDR domain, with no detectable cytotoxicity at concentrations below 3 µM. We recommend the use of OICR-41103 as a DCAF1 WDR domain chemical probe at a 1 µM concentration.

## Methods

### Chemistry methods

All reagents were purchased from commercial vendors and used without further purification. The yields given refer to chromatographically purified and spectroscopically pure compounds, unless stated otherwise. Flash column chromatography was performed on a Teledyne ISCO CombiFlash Rf system using Teledyne ISCO RediSep Rf silica or RediSep Rf C18 cartidges as required. ^1^H, ^19^F and ^13^C NMR spectra were recorded on a Bruker Avance-IV 400 MHz spectrometer at ambient temperature. Residual protons of DMSO-d_6_ or Methanol-d_4_ solvents were used as internal references. ^1^H NMR spectral data are reported as follows: chemical shift (δ in ppm), multiplicity (br = broad, s = singlet, d = doublet, dd = doublet of doublets, t = triplet, m = multiplet), coupling constants (J in Hz) and proton integration. Compound purity was determined by UV absorbance at 254 nm during tandem liquid chromatography/mass spectrometry (LCMS) with a Waters Alliance H class UPLC system using an ACQUITY UPLC BEH-C18 column (1.7 μm, 2.1 mm × 50 mm) with a flow rate of 0.5 mL/min. Elution was carried out using water containing 0.1% formic acid +2 mM NH_4_OAc as mobile phase A and CH_3_CN containing 0.1% formic acid as mobile phase B. Elution with a gradient of 2% B for 0.4 min, 2 to 65% B over 2.1 min, 65% B for 1 min, 65 to 95% B over 1.2 min then 2% B for 0.3 min. The purity of all compounds was >95% by this method. Preparative HPLC was performed on a Shimadzu LC-20AP with a UV detector using a SUNFIRE C18 column (5 mm, 30 mm × 250 mm) with a flow rate of 26 mL/min. Elution was carried out using water containing 0.1% formic acid +2 mM NH_4_OAc as mobile phase A and CH_3_CN as mobile phase B. For compound (**1**) the gradient used was 30 to 50% B over 30 min, 50% B for 9.0 min, 50 to 100% B over 2 min then 100 to 30% B over 5.0 min. For compound (**2**) the gradient used was 25 to 60% B over 24 min, 50% B for 4.0 min, 50 to 100% B over 2 min then 100 to 25% B over 6.0 min. Optical rotations were measured on an Anton Paar MCP 200 polarimeter.

### Cloning, expression and purification of DCAF1 protein for binding studies

The expression construct for biotinylated DCAF1 WDR protein used for binding studies was prepared using a DNA fragment encoding residues 1036–1400 with Avitag on upstream and 3XFlag/Histag on downstream termini designed to subclone under polyhedrin promoter of pFastBacDual vector with BirA sequence under P10 promoter control. The expression construct for the unbiotinylated DCAF1 protein used for thermostability assays was designed in a pFastBacDual vector containing the WDR domain coding sequence 1077–1390 with N-terminal His-tagged under polyhedrin promoter control. Both constructs were synthesized by ThermoFisher Scientific.

The two constructs were processed and expressed in the baculovirus- *Spodoptera frugiperda* 9 (Sf9) cells protein expression system according to the 2021 protocol by Hutchinson and Seitova^54^. The cultures for biotinylated DCAF1 protein were supplemented with biotin to enable in situ biotinylation by the co-expressed BirA biotin ligase. Following expression, the Sf9 culture pellet was collected via centrifugation and stored at −80 °C until required for protein purification.

Both proteins were purified following a two-step protocol involving immobilized metal affinity chromatography (IMAC) and size exclusion chromatography. Briefly, the pelleted Sf9 cells were lysed by sonication and the soluble protein-containing supernatant collected following centrifugation of the crude lysate. The clarified supernatant was then passed through a HispurTM Ni-NTA resin (ThermoFisher; Cat. #88223) column, after which the resin was washed and the protein eluted using 250 mM imidazole-containing buffer. The eluted protein was then concentrated and further purified by gel filtration on a Superdex200 26/60 column (Cytiva; Cat. #28989336) pre-equilibrated with the final protein buffer (50 mM Tris pH 7.5, 150 mM NaCl, 5% glycerol, and 1 mM TCEP) using the BioRad purification system (BioRad; Cat. #7880009). The purity of the fractions was confirmed via SDS-PAGE, whereafter the fractions were pooled, concentrated and flash frozen.

### Expression and purification of WDR Proteins for SPR selectivity assay

The WD40 domain of DCAF1 and six additional WDR proteins (WDR61, WDR92, WDR5, DDB1, FBXW7 and PAFAH1B1) were expressed in biotinylated or 6xHis-tagged forms for SPR selectivity assays. Details of the protein boundaries, expression vectors, tags, and expression systems are provided in Supplementary Table 1.

The biotinylation of six WDR proteins containing an N-terminal AviTag and a C-terminal 6XHis tag was performed in situ via co-expression with the BirA enzyme. In contrast, the PAFAH1B1 protein containing only an N-terminal 6XHis-tag, was ligated with a biotinylated peptide in vitro using the sortase enzyme on an N-terminal glycine after TEV protease cleavage of the His-tag.

DNA fragments encoding the WDR domains were amplified through PCR and cloned into the respective vectors (Supplementary Table 1). The resulting plasmid was transformed into DH10Bac™ competent E. coli cells (Invitrogen) and a recombinant viral bacmid DNA was purified and followed by a recombinant baculovirus generation for baculovirus mediated protein production in Sf9 insect cells with biotin supplementation.

The WDR5 protein was expressed in E. coli BL21 (DE3) pRARE2 cells by inducing them overnight at 16 °C with supplemental biotin.

Following cell lysis, proteins were purified through Ni^2+-^NTA affinity chromatography, followed by gel filtration using an ӒKTA protein purification system and a Superdex S200 26/60 column. For PAFAH1B1, the N-terminal 6XHis-tag was cleaved by overnight incubation with TEV protease, followed by reverse Ni^2+^-NTA affinity chromatography to remove the His-tagged TEV enzyme. The exposed N-terminal glycine of the protein was then ligated to a biotinylated peptide via incubation with the sortase enzyme at room temperature. All proteins were aliquoted and stored at −80 °C until use. LC/MS was used to determine the molecular weight of all proteins and confirm their biotinylation.

### Expression and purification of DCAF1 protein for HTRF assay

The WDR domain of DCAF1 (1038–1400) was expressed and purified as an N-terminally 6xHis-tagged protein for the HTRF based Vpr displacement assays. The DNA fragment encoding the WDR domain of DCAF1 (1038–1400) was amplified through PCR and cloned into the pFBOH-MHL vector. The resulting plasmid was transformed into DH10Bac™ Competent E. coli (Invitrogen) and a recombinant viral bacmid DNA was purified and followed by a recombinant baculovirus generation for baculovirus mediated protein production in Sf9 insect cells. After harvesting and lysing the cells, proteins were purified through Ni^2+^-NTA affinity chromatography and gel filtration on an ӒKTA protein purification system using a Superdex S200 26/60 column. DCAF1 protein was stored in a final storage buffer of 50 mM Tris-HCl pH 8, 150 mM NaCl, 5% glycerol and 5 mM 2-mercaptoethanol. LC/MS was used to confirm the protein had the expected molecular weight, after which it was aliquoted and stored at −80 °C until use.

### Expression and purification of Vpr protein for HTRF assay

The HIV viral Vpr, which binds to the central pocket of the DCAF1 WDR domain, was purified for use in HTRF displacement assays. Due to poor solubility of the full-length protein when expressed alone, the Vpr was expressed with a C-terminal NusA solubility tag to improve the yield^28^. The Vpr construct contained an N-terminal Avi tag removable by a thrombin cleavage site followed by the full-length (1-96) Vpr protein, the NusA solubility tag and finally a C-terminal 6X His tag cleavable by a TEV cut site. The DNA fragments encoding for the Vpr fusion protein were cloned into a pNIC-CH vector and transformed into *E. coli* cells. After growth and expression, cells were harvested through centrifugation and subsequently lysed. Protein was purified using Ni^2^ + -NTA affinity chromatography, after which the C-terminal 6xHis-tag was cleaved using TEV overnight at 4 °C while dialyzing into Ni^2^ + -NTA binding buffer. The His-tag was removed from the sample by flowing the protein through Ni^2^ + -NTA column and then subsequently subjected to gel filtration on a Superdex S200 26/60 column on an ӒKTA protein purification system. Vpr protein was stored at −80 °C in a 20 mM Tris-HCl (pH 8.0), 300 mM NaCl, 1 mM TCEP buffer until use. LC/MS was used to confirm that the produced Vpr protein had the expected molecular weight.

### Surface plasmon resonance (SPR)

SPR experiments for OICR-41103 and OICR-41103N binding studies were performed using a Biacore T200 instrument and a S-series SA (Streptavidin) sensor chip. A single chip was used to immobilize biotinylated DCAF1 in the buffer: 0.01 M HEPES pH 7.4,0.15 M NaCl, 0.05% v/v Surfactant P20. The protein was immobilized in two channels to a signal ranging between 9500 and 10500 RU. All binding experiments were performed in HBS-P buffer: 0.01 M HEPES pH 7.4, 0.15 M NaCl, 0.05% v/v Surfactant P20 with 5% DMSO. Compounds were injected for 60 s followed by buffer for 600 s at a flow rate of 40 μL/min. Compounds were injected in a concentration titration series with a top concentration of 15.625 µM, 7.812 µM, 480 nM, 192 nM or 96 nM, and then titrated down in 1:2 dilution in four dilutions. Injection time was set at 60 s with a flow rate of 40 µL/min followed by a dissociation time of 60 s. All experiments were performed in triplicate. Blank-subtracted sensorgrams were used to extract the maximum response and compounds *K*_D_, On-rate, Off-rate and RU_max_ were measured using kinetic curve fitting.

### Differential scanning fluorimetry (DSF)

DSF experiments were performed using an Applied Biosystems^TM^ QuantStudio^TM^ 5 Real-Time PCR System. Compounds were serially diluted in 10% DMSO before adding to the reactions. Reactions (20 µL) contained 0.1 mg/mL DCAF1 protein with various concentrations of compound in 100 mM Hepes pH 7.5, 150 mM NaCl and 5x SYPRO Orange (Invitrogen, Cat# S6650) with 1.0% final DMSO. The reactions were transferred to a MicroAmp^TM^ EnduraPlate^TM^ Optical 384-well plate (Applied Biosystems, Cat# A36931) and sealed with MicroAmp^TM^ Optical Adhesive Film (Applied Biosystems, Cat# 4311971). Experiment was performed in triplicate. Thermal denaturation was monitored from 25 °C to 95 °C at a rate of 0.066 °C increase per second and data points were collected every second. Data was analyzed using Protein Thermal Shift^TM^ Software v1.4 and data were plotted using GraphPad Prism 10.2.

### SPR - selectivity testing

To assess the selectivity of OICR-41103, we used the DALI web server^55^ to identify WDR domains in the Protein Data Bank (PDB) that are structurally similar to DCAF1. From these, we selected six WDR proteins that had also been successfully purified in our lab during previous projects^56^, enabling their use as negative controls in SPR experiments.

In addition, we evaluated the binding of the negative control R-enantiomer (OICR-41103N) against the same panel of WDR proteins. The following WDR proteins were included in the assay, with their corresponding amino acid boundaries indicated in parentheses; WDR61 (1–305), PAFAH1B1 (86–410), FBXW7 (350–707), WDR92 (1–357), WDR5 (2–334) and DDB1 (1–1140).

Each protein was immobilized onto the second flow cell of separate flow channels on a series S SA sensor chip using a Biacore 8K instrument, with the first flow cell surface left unmodified for reference subtraction. The level of protein immobilization was selected for each protein so that the theoretical maximum signal assuming 1:1 binding for the compound would yield 50 RU (most protein was immobilized between 3000 and 3500 RU above the reference cell signal). Assay were performed at 20 °C in a buffer containing 10 mM HEPES (pH 7.5), 150 mM NaCl, 0.05% Tween-20, 3 mM EDTA and 3% DMSO. Compounds were injected in increasing concentrations using single cycle kinetics mode following a three-fold 8-point serial dilution starting at 20 µM. The flow rate was set to 45 µL/min and compound injections were performed for 80 s at each concentration with a 300 s dissociation period after each injection series. A series of blank injections was performed following the same protocol as before to rinse the sensor chip of any residual compound. Data was analyzed and presented with Biacore Insight Evaluation software.

### Expression and purification of DCAF1 WDR domain for structural studies

The human DCAF1 WDR protein (residues 1077–1390) for structural studies was expressed and purified as described previously^42,48^. Briefly, the DCAF1 WDR gene having residues 1077 (Phe) and 1079 (Arg) mutated to alanines and contained in an in-house insect cell expression vector pFBOH-MHL was expressed as a N-terminally 6xHis-tagged protein in a baculovirus-Sf9 expression system following a protocol by Hutchinson and Seitova^54^. Protein purification was performed by TALON® immobilized cobalt affinity chromatography and clarified by size exclusion chromatography. Briefly, the cell-free extract was incubated with TALON® affinity resin for 1 h, after which the unbound proteins were removed by centrifugation. The resin in an open column was then washed two times with a low imidazole buffer before protein elution with a buffer containing 250 mM Imidazole. The eluted WDR protein was subjected to 6xHis-tag removal by cleavage with the TEV protease overnight, after which the protein sample was re-applied to TALON® resin and the unbound (cleaved) protein collected.

The collected protein was concentrated and loaded onto the HiLoad^TM^ 26/60 Superdex^TM^ 200 gel filtration column on an AKTA Pure chromatography system (GE Healthcare) running in the final protein buffer containing 20 mM Tris-HCl pH 7.5, 150 mM NaCl and 1 mM TCEP. Protein fractions containing pure DCAF1 WDR protein as confirmed by SDS-PAGE were pooled and concentrated using a 10 kDa cutoff spin column (Millipore). The final protein concentration was determined using the Nanodrop (Thermo Scientific) and calculated using the DCAF1 WDR protein extinction coefficient of 35,410 M^–1^ cm^–1^ as computed from the amino acid sequence using Expasy ProtParam (https://web.expasy.org/protparam/).

### Protein crystallization

To generate DCAF1-ligands co-crystals, purified DCAF1 WDR protein at 10 mg/mL (0.281 mM) concentration was mixed with 5 times molar excess of OICR-41103 (1.405 mM) and incubated at room temperature for 15 min prior to crystallization set-up. Crystallization was carried out via screening of an in-house Redwing kit, using the sitting drop vapor-diffusion method by mixing the protein-ligand complex with an equal volume of the reservoir solution in 1 µL drops over 90 μL reservoir. Crystals were observed within 72 h at 18 °C in a precipitant solution containing 20% PEG3350 and 0.2 M di-Ammonium citrate.

### Diffraction data collection, structure determination and refinement

Crystals were cryoprotected by briefly soaking in cryo-solutions containing crystallization mother liquor supplemented with 10% ethylene glycol and 1 mM ligand, before cryo-cooling in liquid nitrogen. Diffraction data were collected on the CMCF-BM beamline at the Canadian Light Source. Diffraction data were processed with HKL3000^57^ and the structure solved by molecular replacement in Phaser^58^ using the DCAF1-8268 crystal structure (PDB ID: 8F8E) as the starting model. The model was refined by alternating cycles of manual rebuilding in Coot^59^ and refinement with Refmac^60^ within the CCP4 crystallographic suite^61^. The refined structure was validated using the Molprobity server^62^. Protein-ligand interactions were analyzed using UCSF Chimera^63^ and ICM-Pro (v. 3.8-2c, MolSoft CA, USA), and the molecular graphics images were rendered using PyMOL^64^.

### Homogeneous time-resolved fluorescence assays (HTRF)

HTRF assays were used to assess the displacement of Vpr from the DCAF1 binding site by probe candidates. This assay utilized an N-terminally biotinylated full-length Vpr protein with a C-terminal NusA solubility tag for ease of purification and storage, an N-terminal 6xHis tag WDR domain of DCAF1 (residues 1038–1400), streptavidin conjugated to XL-665 (Revvity Cat. No. 610SAXLF) and Mab-Anti-6XHis-Tb-cryptate (Revvity Cat. No. 61HI2TLF). Displacement assays were performed with 2.5 nM concentrations of both DCAF1 and Vpr with SA-XL665 and Tb-cryptate anti-6XHis mAb diluted to manufacturer’s specifications. All assays were performed in 20 μL volume in black 384-well Greiner plates (Part No. 784209) diluted in buffer to final concentrations of 20 mM HEPES pH 7.5, 0.05% Tween 20, 5 mM DTT, 150 mM NaCl, and 2% DMSO. Proteins were incubated for 2 h at RT before incubation with compounds for up to 24 h. Fluorescence emission at 620/10 nm and 665/10 nm was read in a Biotek Synergy H1 multimode plate reader following excitation at 330/80 nm using a xenon flash lamp. HTRF signal is defined as fluorescence ratio between reads at 665 nm to 620 nm and blank subtracted by the equivalent ratio in the reference wells (same assay components without DCAF1 and Vpr). For displacement assays the HTRF signal was expressed as a percent of the HTRF signal without the addition of compound. *K*_disp_ values for given compounds were determined by fitting a concentration versus response four variable curve against the average % HTRF signal at each concentration using GraphPad Prism 10 software.

### Cell culture

The following cell lines: NCI-H1703, NCI-H1915, NCI-H2170, MCF7, U2Os and HCT116 were purchased from the American Type Culture Collection (ATCC) and HEK293T cells are a kind gift from Dr. Sam Benchimol, York University. NCI-H1703, NCI-H1915, NCI-H2170 cells were cultured in RPMI (Life Tech, 11875-119) supplemented with 10% FBS (Corning, 35075CV) [MS1] at 37 °C in a 5% CO_2_ incubator. MCF7, U2OS and HEK293T were cultured in DMEM (Wisent) and HCT116 in RPMI (Wisent) supplemented with 10% FBS and penicillin (100 U.mL^−1^) and streptomycin (100 µg.mL^−1^) (Wisent). All cell lines were mycoplasma negative, as determined by MycoAlert™ Mycoplasma Detection Kit (Lonza).

### Cellular thermal shift assay (CETSA)

Two hundred thousand NCI-H460 cells constitutively expressing Flag-HiBiT-WD40DCAF1 (1038-1400aa) were seeded in a 96-well PCR plate (Eurofins DiscoverX; 92-0031) with RPMI + 10% FBS. DMSO, OICR-41103, or OICR-41103N

were added using the HP D300e Digital Dispenser, then incubated for 3 h at 37 °C in a 5% CO_2_ incubator. The plate containing the cells was heated at 59 °C using C1000 Touch Thermal Cycler (Bio-Rad; 185119) for 3 min and 30 s. NanoGlo HiBiT Lytic (Promega N3040), containing the LgBiT and furimazine substrate, was added to the plate. An orbital shaker was used to shake the plate for 10 min at 500 rpm. The luminescence signal was measured using the BioTek Cytation 3 imaging reader (Agilent).

### NanoBRET displacement assay with a DCAF1 Tracer

DCAF1(WD40, 1038-1400 aa) was cloned into the pNLF1-N vector (Promega). HEK293T cells were plated in 6-well plate (8 ×105 cells/well) and reverse-transfected with 0.2 µg of N-terminally NL-tagged DCAF1(WD40) vector and 1.8 µg of empty vector using XtremeGene HP transfection reagent (Roche), following manufacturer’s instructions. Next day cells were trypsinized and resuspended in optiMEM (no phenol red, Gibco) at 2 ×105 cells/ml density with 1 µM of DCAF1 tracer (Synthesis described in Supporting Information). Compound serial dilutions were prepared in DMSO and added to cells. Cells were transferred to 384-well white low binding white plates (10 µL/well, Corning #3574. After 1 h, 5 µL/well of NanoBRET™ Nano-Glo® Substrate (Promega) and Extracellular NanoLuc® Inhibitor (Promega) diluted in optiMEM (no phenol red) 200-fold and 500-fold, respectively, was added. The donor emission at 450 nm and acceptor emission at 618 nm was read immediately after substrate addition and shaking plate for 20 s using ClarioStar plate reader. NanoBRET ratios were calculated by subtracting the mean of 610/460 nm signal from cells without tracer ×1000 from the 610/460 nm signal from cells with tracer ×1000.

### DCAF1 WDR domain and Vpr disruption NanoBiT assay

Promega’s NanoLuc Binary Technology (NanoBiT) protein-protein interaction system was utilized and modified to develop the WD40-DCAF1 domain and Vpr disruption assay (N2014). WD40 (1038-1400aa) DCAF1-smBiT and LgBiT-Vpr cDNA constructs were synthesized and cloned into pcDNA3.1 (+) plasmids by GeneArt Gene Synthesis (Invitrogen), while smBiT-MDM2 and LgBiT-p53 cDNA constructs (NanoBiT™ PPI Control Pair) were purchased from Promega (CS1603B09).

HEK293EMT cells were transfected with the respective construct pairs in a 1:1 DNA ratio and 3:1 DNA to FuGENE® 6 Transfection Reagent (Promega; E2691). After 16–24 h of transfection, cells were seeded in 96-well white plates (PerkinElmer; 6005680). The HP D300e Digital Dispenser was used to add DMSO, OICR-41103, or OICR-41103N, and the cells were incubated for 3 h at 37 °C in a 5% CO2 incubator. Cells were lysed with Nano-Glo® Luciferase Assay Buffer (Promega; N1120) and then Nano-Glo® Luciferase Assay Substrate (Promega; N1120) was added. BioTek Cytation 3 imaging reader (Agilent) was used to measure the signal of reconstituted nanoluciferase.

### Western blotting

NCI-H1703, NCI-H1915, and NCI-H2170 cells were trypsinized and pelleted by centrifugation at 500 × *g* for 5 min. Cells were lysed using M-PER lysis buffer (ThermoFisher, 78501), 1% SDS, and 1X Halt protease and phosphatase inhibitor cocktail (ThermoFisher, 78444). The lysate was sonicated, centrifuged for 10 min at 10,000 × *g*, and transferred to a new 1.5 mL Eppendorf tube. The Colorimetric DC Protein Assay (BioRad, 500-0207) was used to quantify the amount of protein in each lysate. Sodium dodecyl sulfate–polyacrylamide gel electrophoresis (SDS-PAGE) was used to run and separate the samples. The iBlot2 transfer system by Invitrogen (IB21001) was used to transfer the samples onto a polyvinylidene difluoride (PVDF) membrane (IB24001). Anti-DCAF1 (CST, 14966S, 1:1000) and anti-GAPDH (CST, 2118S, 1:5000) antibodies were used to detect the respective proteins and HRP-conjugated goat anti-rabbit secondary antibody (Bio-Rad, 1706515, 1:10,000) was used to detect the primary antibodies. After the addition of Immobilon chemiluminescent HRP substrate (Thermo Scientific), ChemiDoc imager was used to visualize the different bands on the membrane.

### Trypan blue exclusion assay

Vi-CELL XR Cell Viability Analyzer (Beckman Coulter) was used to count the number of cells in doxycycline treated and untreated flasks. Cells were seeded (0.5–1.5 million) at day 0 in a T25 flask and counted at different time points after the addition of 0.1 μg/mL doxycycline. The Vi-Cell automates cell viability assessment by mixing the cells with trypan blue dye. The system is equipped with a camera to capture images and uses images analysis to count the number of live and dead cells. Trypan blue stains dead cells, while live cells remain unstained.

### ATPlite assay

The ATPlite 1 step kit by Perkin Elmer (6016739) was used as a proxy assay to measure the effect of OICR-41103 and OICR-41103N on cell count and cell proliferation. NCI-H1703 (250 cells), NCI-H1915 (500 cells), and NCI-H2170 (500 cells) were seeded in 150 μL in a 96-well plate. HP D300e Digital Dispenser was used to add compounds and then cells were incubated for 7 days at 37 °C in a 5% CO_2_ incubator. After 7 days, 150 μL of reconstituted ATPlite reagent (containing luciferase and D-luciferin) was added to the cells to measure the levels of ATP. Biotek Cytation 3 plate reader was used to measure the levels of emitted light, which is proportional to ATP levels.

### Cytotoxicity

Different cell lines were seeded on 96-well and treated with compounds for 5 days. MCF7 (5 ×103 cells/well), U2Os (1 ×103 cells/well) and HEK293T (2 ×103 cells/well) were grown in DMEM and HCT116 (2 ×103 cells/well) in RPMI supplemented with 10% FBS (Wisent), penicillin (100 units/mL) and streptomycin (100 µg/mL). The compounds were topped up after 3 days. The confluency was measured using IncuCyte™ ZOOM live cell imaging device (Essen Bioscence) and analyzed with IncuCyte™ ZOOM (2023A) software based on phase contrast images.

### Statistics and reproducibility

For the following experiments: CETSA, WD40-DCAF1/Vpr protein-protein interaction NanoBiT assay, and cell count assays (Trypan blue exclusion and ATPlite), all cellular assays were independently performed at least three times unless otherwise stated. Results are presented as mean ± standard deviation (SD). Statistical analysis was performed for the Trypan blue exclusion assay using an unpaired two-tailed Student’s *t* test, and *p*-values < 0.05 were considered statistically significant. Sample sizes and number of replicates are indicated in the figure legends where applicable.

### Reporting summary

Further information on research design is available in the Nature Portfolio Reporting Summary linked to this article.

## Supplementary information

Supplemental Information

Description of Additional Supplementary Files

Supplementary Data

Reporting Summary

## Data Availability

Atomic coordinates and structure factors for DCAF1-OICR-41103 structure have been deposited in the Protein Data bank under the accession code: 9D4E. Source data for Figs. 2A–D, 3A, B, 4C, D, and 5A–C, Supplementary Figs. 3, 7A–E, and 8 are available in the supplementary data. Uncropped and unedited Fig. 5A blot/gel images are available in the Supplementary Fig. 9.

## References

[1] Maddika, S. & Chen, J. Protein kinase DYRK2 is a scaffold that facilitates assembly of an E3 ligase. *Nat. Cell Biol.* **11**, 409–419 (2009). 19287380 10.1038/ncb1848 PMC2754075

[2] Angers, S. et al. Molecular architecture and assembly of the DDB1-CUL4A ubiquitin ligase machinery. *Nature* **443**, 590–593 (2006). 16964240 10.1038/nature05175

[3] He, Y. J., McCall, C. M., Hu, J., Zeng, Y. & Xiong, Y. DDB1 functions as a linker to recruit receptor WD40 proteins to CUL4-ROC1 ubiquitin ligases. *Genes Dev.* **20**, 2949–2954 (2006). 17079684 10.1101/gad.1483206 PMC1620025

[4] Kim, K. et al. VprBP has intrinsic kinase activity targeting histone H2A and represses gene transcription. *Mol. Cell* **52**, 459–467 (2013). 24140421 10.1016/j.molcel.2013.09.017 PMC3851289

[5] Mohamed, W. I. et al. The CRL4(DCAF1) cullin-RING ubiquitin ligase is activated following a switch in oligomerization state. *EMBO J.* **40**, e108008 (2021). 34595758 10.15252/embj.2021108008 PMC8591539

[6] Wang, D. et al. Acetylation-regulated interaction between p53 and SET reveals a widespread regulatory mode. *Nature* **538**, 118–122 (2016). 27626385 10.1038/nature19759 PMC5333498

[7] Hossain, D., Javadi Esfehani, Y., Das, A. & Tsang, W. Y. Cep78 controls centrosome homeostasis by inhibiting EDD-DYRK2-DDB1(Vpr)(BP). *EMBO Rep.* **18**, 632–644 (2017). 28242748 10.15252/embr.201642377 PMC5376967

[8] Jung, H. Y., Wang, X., Jun, S. & Park, J. I. Dyrk2-associated EDD-DDB1-VprBP E3 ligase inhibits telomerase by TERT degradation. *J. Biol. Chem.* **288**, 7252–7262 (2013). 23362280 10.1074/jbc.M112.416792 PMC3591633

[9] Sharma, A., Kaur, M., Kar, A., Ranade, S. M. & Saxena, S. Ultraviolet radiation stress triggers the down-regulation of essential replication factor Mcm10. *J. Biol. Chem.* **285**, 8352–8362 (2010). 20064936 10.1074/jbc.M109.041129 PMC2832985

[10] Kaur, M., Khan, M. M., Kar, A., Sharma, A. & Saxena, S. CRL4-DDB1-VPRBP ubiquitin ligase mediates the stress triggered proteolysis of Mcm10. *Nucleic Acids Res.* **40**, 7332–7346 (2012). 22570418 10.1093/nar/gks366 PMC3424545

[11] Guo, Z. et al. DCAF1 controls T-cell function via p53-dependent and -independent mechanisms. *Nat. Commun.* **7**, 10307 (2016). 26728942 10.1038/ncomms10307 PMC4728445

[12] Yu, C. et al. CRL4 complex regulates mammalian oocyte survival and reprogramming by activation of TET proteins. *Science* **342**, 1518–1521 (2013). 24357321 10.1126/science.1244587

[13] Nakagawa, T. et al. CRL4(VprBP) E3 ligase promotes monoubiquitylation and chromatin binding of TET dioxygenases. *Mol. Cell* **57**, 247–260 (2015). 25557551 10.1016/j.molcel.2014.12.002 PMC4304937

[14] Yu, C., Ji, S. Y., Sha, Q. Q., Sun, Q. Y. & Fan, H. Y. CRL4-DCAF1 ubiquitin E3 ligase directs protein phosphatase 2A degradation to control oocyte meiotic maturation. *Nat. Commun.* **6**, 8017 (2015). 26281983 10.1038/ncomms9017 PMC4557334

[15] Kassmeier, M. D. et al. VprBP binds full-length RAG1 and is required for B-cell development and V(D)J recombination fidelity. *EMBO J.* **31**, 945–958 (2012). 22157821 10.1038/emboj.2011.455 PMC3280554

[16] Li, Y. et al. VprBP mitigates TGF-beta and Activin signaling by promoting Smurf1-mediated type I receptor degradation. *J. Mol. Cell Biol.* **12**, 138–151 (2020). 31291647 10.1093/jmcb/mjz057 PMC7109606

[17] Schabla, N. M., Perry, G. A., Palmer, V. L. & Swanson, P. C. VprBP (DCAF1) regulates RAG1 expression independently of dicer by mediating RAG1 degradation. *J. Immunol.* **201**, 930–939 (2018). 29925675 10.4049/jimmunol.1800054 PMC6084458

[18] Jung, E. S. et al. Jmjd2C increases MyoD transcriptional activity through inhibiting G9a-dependent MyoD degradation. *Biochim. Biophys. Acta* **1849**, 1081–1094 (2015). 26149774 10.1016/j.bbagrm.2015.07.001

[19] Karim, M. F., Yoshizawa, T., Sobuz, S. U., Sato, Y. & Yamagata, K. Sirtuin 7-dependent deacetylation of DDB1 regulates the expression of nuclear receptor TR4. *Biochem. Biophys. Res Commun.* **490**, 423–428 (2017). 28623141 10.1016/j.bbrc.2017.06.057

[20] Han, X. R. et al. CRL4(DCAF1/VprBP) E3 ubiquitin ligase controls ribosome biogenesis, cell proliferation, and development. *Sci. Adv.* **6**, 10.1126/sciadv.abd6078 (2020). 10.1126/sciadv.abd6078 PMC11206221 33355139

[21] Ghate, N. B. et al. VprBP/DCAF1 regulates p53 function and stability through site-specific phosphorylation. *Oncogene* **42**, 1405–1416 (2023). 37041410 10.1038/s41388-023-02685-8 PMC10121470

[22] Ghate, N. B. et al. Phosphorylation and stabilization of EZH2 by DCAF1/VprBP trigger aberrant gene silencing in colon cancer. *Nat. Commun.* **14**, 2140 (2023). 37069142 10.1038/s41467-023-37883-1 PMC10110550

[23] Wang, X. et al. VprBP/DCAF1 Regulates the Degradation and Nonproteolytic Activation of the Cell Cycle Transcription Factor FoxM1. *Mol. Cell Biol.* **37**, 10.1128/MCB.00609-16 (2017). 10.1128/MCB.00609-16 PMC5472828 28416635

[24] Yan, J., Shun, M. C., Zhang, Y., Hao, C. & Skowronski, J. HIV-1 Vpr counteracts HLTF-mediated restriction of HIV-1 infection in T cells. *Proc. Natl. Acad. Sci. USA* **116**, 9568–9577 (2019). 31019079 10.1073/pnas.1818401116 PMC6511057

[25] Zhou, X. et al. HIV-1 Vpr protein directly loads helicase-like transcription factor (HLTF) onto the CRL4-DCAF1 E3 ubiquitin ligase. *J. Biol. Chem.* **292**, 21117–21127 (2017). 29079575 10.1074/jbc.M117.798801 PMC5743084

[26] Hrecka, K. et al. HIV-1 and HIV-2 exhibit divergent interactions with HLTF and UNG2 DNA repair proteins. *Proc. Natl. Acad. Sci. USA* **113**, E3921–3930 (2016). 27335459 10.1073/pnas.1605023113 PMC4941427

[27] Lahouassa, H. et al. HIV-1 Vpr degrades the HLTF DNA translocase in T cells and macrophages. *Proc. Natl. Acad. Sci. USA* **113**, 5311–5316 (2016). 27114546 10.1073/pnas.1600485113 PMC4868422

[28] Wu, Y. et al. The DDB1-DCAF1-Vpr-UNG2 crystal structure reveals how HIV-1 Vpr steers human UNG2 toward destruction. *Nat. Struct. Mol. Biol.* **23**, 933–940 (2016). 27571178 10.1038/nsmb.3284 PMC5385928

[29] Wen, X., Casey Klockow, L., Nekorchuk, M., Sharifi, H. J. & de Noronha, C. M. The HIV1 protein Vpr acts to enhance constitutive DCAF1-dependent UNG2 turnover. *PLoS One* **7**, e30939 (2012). 22292079 10.1371/journal.pone.0030939 PMC3265533

[30] Ahn, J. et al. HIV-1 Vpr loads uracil DNA glycosylase-2 onto DCAF1, a substrate recognition subunit of a cullin 4A-ring E3 ubiquitin ligase for proteasome-dependent degradation. *J. Biol. Chem.* **285**, 37333–37341 (2010). 20870715 10.1074/jbc.M110.133181 PMC2988339

[31] Zhou, X., DeLucia, M. & Ahn, J. SLX4-SLX1 Protein-independent Down-regulation of MUS81-EME1 Protein by HIV-1 Viral Protein R (Vpr). *J. Biol. Chem.* **291**, 16936–16947 (2016). 27354282 10.1074/jbc.M116.721183 PMC5016100

[32] Yurkovetskiy, L. et al. Primate immunodeficiency virus proteins Vpx and Vpr counteract transcriptional repression of proviruses by the HUSH complex. *Nat. Microbiol.* **3**, 1354–1361 (2018). 30297740 10.1038/s41564-018-0256-x PMC6258279

[33] Chougui, G. et al. HIV-2/SIV viral protein X counteracts HUSH repressor complex. *Nat. Microbiol.* **3**, 891–897 (2018). 29891865 10.1038/s41564-018-0179-6

[34] Forouzanfar, F. et al. HIV-1 Vpr mediates the depletion of the cellular repressor CTIP2 to counteract viral gene silencing. *Sci. Rep.* **9**, 13154 (2019). 31511615 10.1038/s41598-019-48689-x PMC6739472

[35] Maudet, C. et al. HIV-1 Vpr induces the degradation of ZIP and sZIP, adaptors of the NuRD chromatin remodeling complex, by hijacking DCAF1/VprBP. *PLoS One* **8**, e77320 (2013). 24116224 10.1371/journal.pone.0077320 PMC3792905

[36] Kim, K. et al. Vpr-binding protein antagonizes p53-mediated transcription via direct interaction with H3 tail. *Mol. Cell Biol.* **32**, 783–796 (2012). 22184063 10.1128/MCB.06037-11 PMC3272969

[37] Wang, B. S. et al. Autophagy negatively regulates cancer cell proliferation via selectively targeting VPRBP. *Clin. Sci.* **124**, 203–214 (2013). 10.1042/CS20120270 22963397

[38] Yamashita, K. et al. Tumor suppressor protein Lgl mediates G1 cell cycle arrest at high cell density by forming an Lgl-VprBP-DDB1 complex. *Mol. Biol. Cell* **26**, 2426–2438 (2015). 25947136 10.1091/mbc.E14-10-1462 PMC4571298

[39] Li, W. et al. Merlin/NF2 suppresses tumorigenesis by inhibiting the E3 ubiquitin ligase CRL4(DCAF1) in the nucleus. *Cell* **140**, 477–490 (2010). 20178741 10.1016/j.cell.2010.01.029 PMC2828953

[40] Li, W. et al. Merlin/NF2 loss-driven tumorigenesis linked to CRL4(DCAF1)-mediated inhibition of the hippo pathway kinases Lats1 and 2 in the nucleus. *Cancer Cell* **26**, 48–60 (2014). 25026211 10.1016/j.ccr.2014.05.001 PMC4126592

[41] Poulose, N. et al. VPRBP functions downstream of the androgen receptor and OGT to restrict p53 activation in prostate cancer. *Mol. Cancer Res.* **20**, 1047–1060 (2022). 35348747 10.1158/1541-7786.MCR-21-0477 PMC9381113

[42] Li, A. S. M. et al. Discovery of nanomolar DCAF1 small molecule ligands. *J. Med. Chem*. 10.1021/acs.jmedchem.2c02132 (2023). 10.1021/acs.jmedchem.2c02132 PMC10108359 36948210

[43] Mabanglo, M. F. et al. Crystal structures of DCAF1-PROTAC-WDR5 ternary complexes provide insight into DCAF1 substrate specificity. *Nat. Commun.* **15**, 10165 (2024). 39580491 10.1038/s41467-024-54500-x PMC11585590

[44] Vulpetti, A. et al. Discovery of new binders for DCAF1, an emerging ligase target in the targeted protein degradation field. *ACS Med. Chem. Lett.* **14**, 949–954 (2023). 37465299 10.1021/acsmedchemlett.3c00104 PMC10350940

[45] Schroder, M. et al. DCAF1-based PROTACs with activity against clinically validated targets overcoming intrinsic- and acquired-degrader resistance. *Nat. Commun.* **15**, 275 (2024). 38177131 10.1038/s41467-023-44237-4 PMC10766610

[46] Schwefel, D. et al. Molecular determinants for recognition of divergent SAMHD1 proteins by the lentiviral accessory protein Vpx. *Cell Host Microbe* **17**, 489–499 (2015). 25856754 10.1016/j.chom.2015.03.004 PMC4400269

[47] Sharma, P. & Nag, A. CUL4A ubiquitin ligase: a promising drug target for cancer and other human diseases. *Open Biol.* **4**, 130217 (2014). 24522884 10.1098/rsob.130217 PMC3938054

[48] Kimani, S. W. et al. Discovery of a novel DCAF1 ligand using a drug-target interaction prediction model: generalizing machine learning to new drug targets. *J. Chem. Inf. Model.* **63**, 4070–4078 (2023). 37350740 10.1021/acs.jcim.3c00082 PMC10337664

[49] Zhang, S., Feng, Y., Narayan, O. & Zhao, L. J. Cytoplasmic retention of HIV-1 regulatory protein Vpr by protein-protein interaction with a novel human cytoplasmic protein VprBP. *Gene* **263**, 131–140 (2001). 11223251 10.1016/s0378-1119(00)00583-7

[50] Hossain, D., Ferreira Barbosa, J. A., Cohen, E. A. & Tsang, W. Y. HIV-1 Vpr hijacks EDD-DYRK2-DDB1(DCAF1) to disrupt centrosome homeostasis. *J. Biol. Chem.* **293**, 9448–9460 (2018). 29724823 10.1074/jbc.RA117.001444 PMC6005440

[51] Nakagawa, T., Mondal, K. & Swanson, P. C. VprBP (DCAF1): a promiscuous substrate recognition subunit that incorporates into both RING-family CRL4 and HECT-family EDD/UBR5 E3 ubiquitin ligases. *BMC Mol. Biol.* **14**, 22 (2013). 24028781 10.1186/1471-2199-14-22 PMC3847654

[52] Schabla, N. M., Mondal, K. & Swanson, P. C. DCAF1 (VprBP): emerging physiological roles for a unique dual-service E3 ubiquitin ligase substrate receptor. *J. Mol. Cell Biol.* **11**, 725–735 (2019). 30590706 10.1093/jmcb/mjy085 PMC6821201

[53] Cancer Dependency Map, https://depmap.org/portal/ (2025).

[54] Hutchinson, A. & Seitova, A. Production of recombinant PRMT proteins using the baculovirus expression vector system. *J Vis Exp*. 10.3791/62510 (2021). 10.3791/62510 34338677

[55] Holm, L., Laiho, A., Toronen, P. & Salgado, M. DALI shines a light on remote homologs: one hundred discoveries. *Protein Sci.* **32**, e4519 (2023). 36419248 10.1002/pro.4519 PMC9793968

[56] Ackloo, S. et al. A target class ligandability evaluation of WD40 repeat-containing proteins. *J. Med. Chem.* **68**, 1092–1112 (2025). 39495097 10.1021/acs.jmedchem.4c02010 PMC11770632

[57] Minor, W., Cymborowski, M., Otwinowski, Z. & Chruszcz, M. HKL-3000: the integration of data reduction and structure solution-from diffraction images to an initial model in minutes. *Acta Crystallogr. D. Biol. Crystallogr.* **62**, 859–866 (2006). 16855301 10.1107/S0907444906019949

[58] McCoy, A. J. et al. Phaser crystallographic software. *J. Appl. Crystallogr.* **40**, 658–674 (2007). 19461840 10.1107/S0021889807021206 PMC2483472

[59] Emsley, P. & Cowtan, K. Coot: model-building tools for molecular graphics. *Acta Crystallogr D. Biol. Crystallogr* **60**, 2126–2132 (2004). 15572765 10.1107/S0907444904019158

[60] Murshudov, G. N., Vagin, A. A. & Dodson, E. J. Refinement of macromolecular structures by the maximum-likelihood method. *Acta Crystallogr. D. Biol. Crystallogr.* **53**, 240–255 (1997). 15299926 10.1107/S0907444996012255

[61] Winn, M. D. et al. Overview of the CCP4 suite and current developments. *Acta Crystallogr. D. Biol. Crystallogr.* **67**, 235–242 (2011). 21460441 10.1107/S0907444910045749 PMC3069738

[62] Chen, V. B. et al. MolProbity: all-atom structure validation for macromolecular crystallography. *Acta Crystallogr. D. Biol. Crystallogr.* **66**, 12–21 (2010). 20057044 10.1107/S0907444909042073 PMC2803126

[63] Pettersen, E. F. et al. UCSF Chimera-a visualization system for exploratory research and analysis. *J. Comput Chem.* **25**, 1605–1612 (2004). 15264254 10.1002/jcc.20084

[64] DeLano, W. & Schrödinger, L. Pymol, http://www.pymol.org/pymol (2002).

