# Supplementary material for: OICR-41103 as a chemical probe for the DCAF1 WD40 domain
Source: Commun Biol. 2025 Jul 19;8:1076. doi: 10.1038/s42003-025-08491-0 (PMC12276300; doi:10.1038/s42003-025-08491-0)
Supplement: Supplementary file 1 — Supplemental Information [file 42003_2025_8491_MOESM1_ESM.pdf]

OICR-41103 as a Chemical Probe for the DCAF1 WD40 Domain

Serah W. Kimani,[1†], Mahmoud Noureldin,[2,3†], Brian Wilson, [2†] Laurent Hoffer,[2] Stuart R. Green,[1] Magdalena M. Szewczyk,[1] Héctor González-Álvarez,[2,3] Mohammed Mohammed,[2] Manuel Chan,[2] Chiara Krausser[2], Alice Shi Ming Li,[2,3] Taraneh Hajian,[2] Sarah Tucker,[2] Dhananjay Joshi, [2] Punit Saraon,[2] Brigitte Thériault,[2] Ji Sup Kim,[2] Vijayaratnam Santhakumar,[1] Peter Loppnau,[1] Yanjun Li,[1] Almagul Seitova,[1] Aiping Dong, [1] Taira Kiyota,[2] Tobias Hammann, [4] Paul Gehrtz, [4] Bhashant Patel,[5] Vaibhavi Rathod,[5] Anand Vala,[5] Bhimsen Rout,[5] Paras Jagodra,[5] Peter J. Brown[6], Ahmed Aman,[2,7] Jailall Ramnauth,[2] Gennady Poda,[2,7] David Uehling,[2] Cheryl H. Arrowsmith, [1,8,9] Dalia Barsyte-Lovejoy,[1,3] Richard Marcellus,[2] Suzanne Ackloo,[1] Ahmed Mamai, [2] Rima Al-awar,[2,3,10]*, Levon Halabelian,[1,3]*

[1]Structural Genomics Consortium, University of Toronto, Toronto, Ontario M5G 1L7, Canada.
[2]Drug Discovery Program, Ontario Institute for Cancer Research, Toronto, Ontario M5G 0A3, Canada.
[3]Department of Pharmacology and Toxicology, University of Toronto, Toronto, Ontario M5S 1A8, Canada.
[4]Medicinal Chemistry, Global Research & Development, Merck Healthcare KGaA, 64293 Darmstadt, Germany.
[5]Piramal Discovery Solutions, Pharmaceutical Special Economic Zone, Ahmedabad, Gujarat 382213, India.
[6]Structural Genomics Consortium, Eshelman School of Pharmacy, University of North Carolina at Chapel Hill, Chapel Hill, NC 27599, USA.
[7]Leslie Dan Faculty of Pharmacy, University of Toronto, Toronto, Ontario M5S 3M2, Canada.
[8]Princess Margaret Cancer Centre, University Health Network, Toronto, Ontario, M5G 1L7, Canada.
[9]Department of Medical Biophysics, University of Toronto, Toronto, Ontario M5G 1L7 Canada
[10]Department of Chemistry, University of Toronto, Toronto, Ontario M5S 3H6, Canada.

[†]These authors contributed equally.
*Corresponding Authors:
Rima Al-awar: ralawar@oicr.on.ca; Drug Discovery Program, Ontario Institute for Cancer Research, Toronto, Ontario M5G 0A3, Canada; Department of Pharmacology and Toxicology, University of Toronto, Toronto, Ontario M5S 1A8, Canada; Department of Chemistry, University of Toronto, Toronto, Ontario M5S 3H6, Canada.

Levon Halabelian: l.halabelian@utoronto.ca; Tel: 416 946 3876; Structural Genomics Consortium, Toronto, Ontario M5G 1L7, Canada; Department of Pharmacology and Toxicology, University of Toronto, Toronto, Ontario M5S 1A8, Canada.

Supplementary information

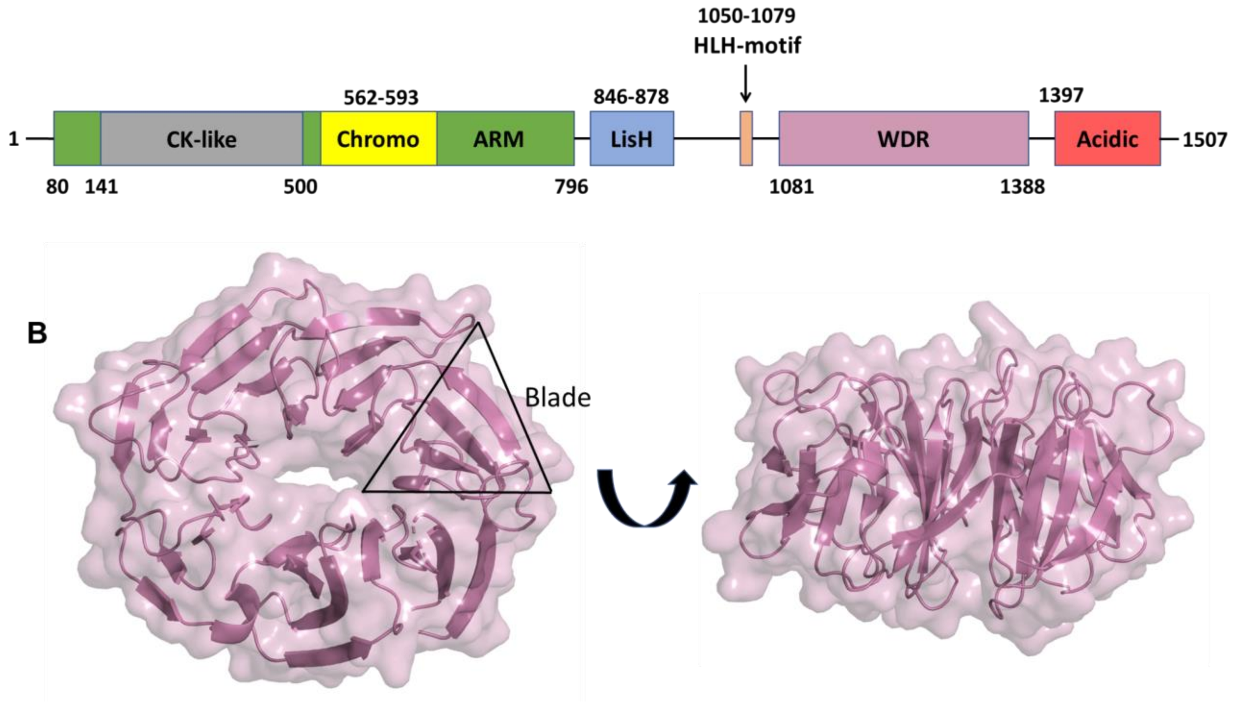

Supplementary Figure 1: The human DCAF1 protein domains. (A) The DCAF1 domains schematic. The alpha-helical Armadillo (ARM) repeats domain, the helix-loop-helix H-box motif and the acidic domain mediate protein-protein interactions. The casein kinase-like (CK-like) domain has phosphorylation activity, the chromo-like domain mediates interactions with methylated proteins, while the Lis1-homology (LisH) domain mediates dimerization of DCAF1. The WD40 repeat (WDR) domain that is the subject of this study is a protein-protein interaction module that mediates recruitment of substrate proteins to the CRL4 and EDVR E3 ligase complexes. (B) A transparent surface and cartoon representation of the DCAF1 WDR domain β-propeller structure (PDB ID: 4PXW). One of the seven WD40 blades is highlighted.

Supplementary Table 1: **Construct boundaries and protein expression details for the WDR domain proteins used for compound selectivity studies.**

Protein (UniProt ID)	Amino acid range	Expression vector	Protein tags	Expression system	Biotinylation
**DCAF1** (Q9Y4B6)	1038-1400	pFBD-BirA	N-terminal Avi C-terminal 6XHis	BVES/Sf9	*In situ* - BirA biotin ligase
**WDR61** (Q9GZS3)	1-305	pFBD-BirA	N-terminal Avi C-terminal 6XHis	BVES/Sf9	*In situ - BirA biotin ligase*
**WDR92** (Q96MX6)	1-357	pFBD-BirA	N-terminal Avi C-terminal 6XHis	BVES/Sf9	*In situ - BirA biotin ligase*
**PAFAH1B1** (P43034)	86-410	pFBOH-MHL	N-terminal 6XHis (cleaved with TEV)	BVES/Sf9	*In vitro* with sortase
**WDR5** (P61964)	2 - 334	pNICBio2	N-terminal Avi C-terminal 6XHis	*E. coli*	*In situ - BirA biotin ligase*
**FBXW7** (Q969H0)	350-707	pFBOH-avi-BirAD-BirA	N-terminal 6XHis-Avi	BVES/Sf9	*In situ - BirA biotin ligase*
**DDB1** (Q16531)	1-1140	pFBD-BirA	N-terminal Avi C-terminal 6XHis	BVES/Sf9	*In situ - BirA biotin ligase*

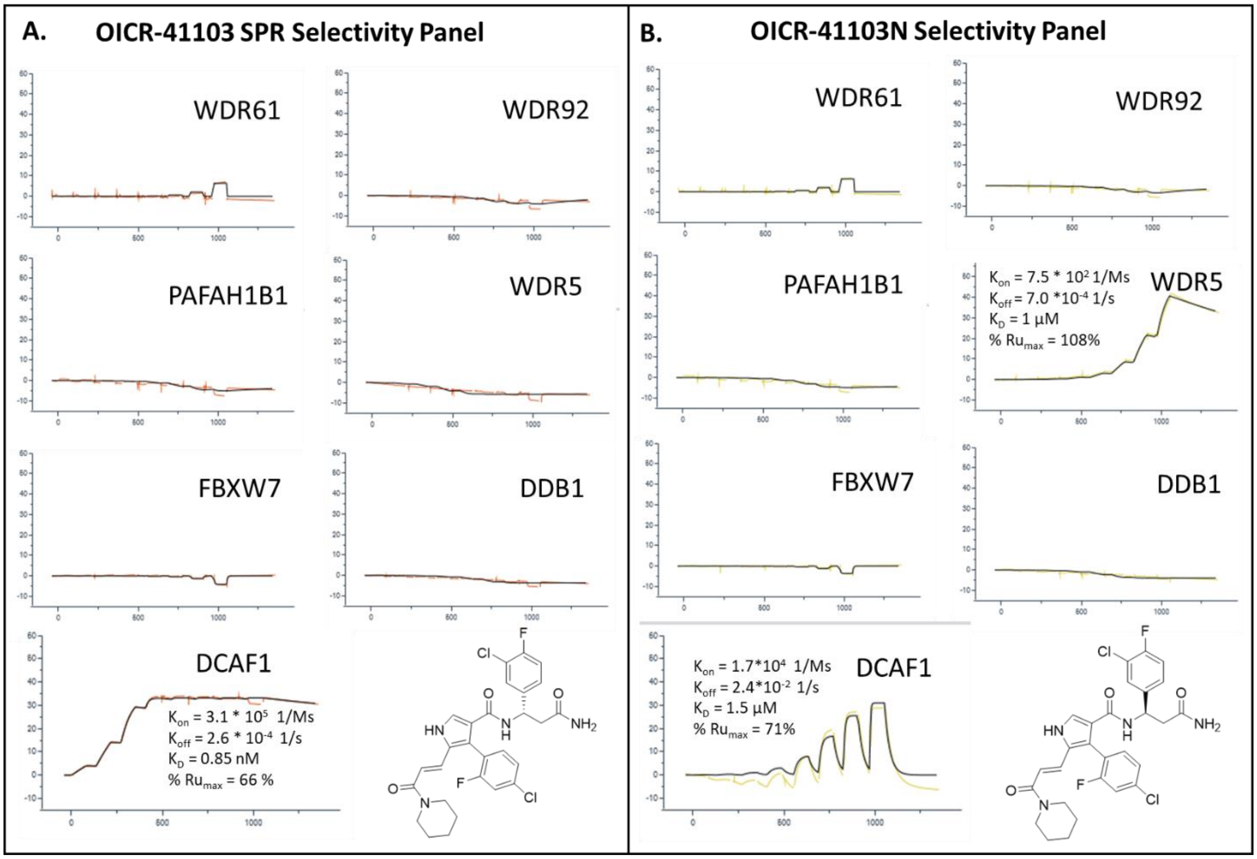

Supplementary Figure 2: Probing a panel of 6 WDR proteins on SPR to test for selectivity of the DCAF1-OICR-41103 interaction. A. Representative sensorgrams of the data displaying the reference subtracted response units (RU) over time during successive OICR-41103 injections with increasing concentration show that none of the WDR proteins demonstrated any significant binding to OICR-41103, despite the K_D for the strong interaction between DCAF1 and the molecule (~1 nM) and the maximum concentration applied being 20 µM. **B.** Representative sensorgrams for the OICR-41103N negative control compound show similar binding response as the DCAF1 probe compound OICR-41103, except for WDR5 which demonstrated a 1 µM K_D. Maximum theoretical RU is ~50 for each protein assuming 1:1 binding. Kinetic fitting is shown as the black line for each data set. Concentrations were increased from 9.15 nM to 20 µM.

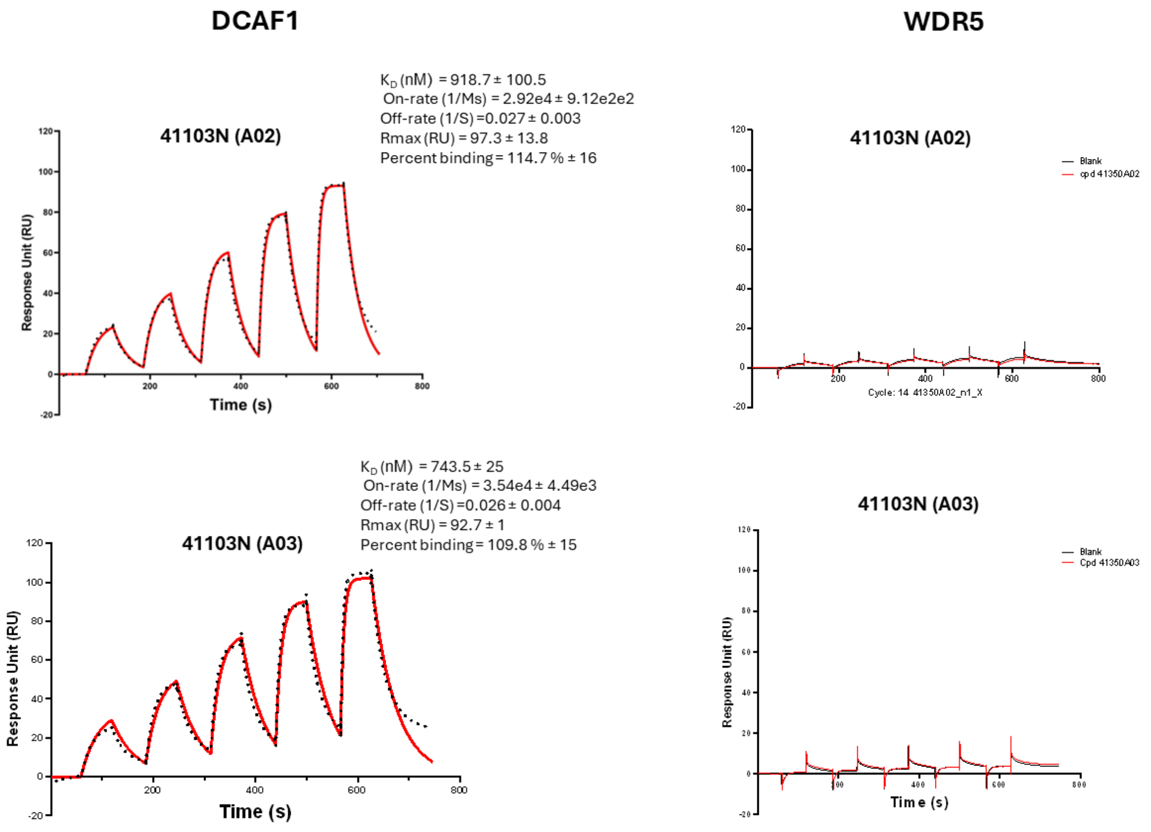

Supplementary Figure 3: SPR sensorgrams depicting the binding behavior of OICR-41103N batches A02 and A03 to the WD40 domain of DCAF1 and WDR5. (**A**) Sensorgrams for batch A02 demonstrate a lack of binding to the WDR5 protein. (**B**) Similarly, sensorgrams for batch A03 (a repurified version of batch A01) show no binding interaction with WDR5. These results, in contrast to the binding observed with batch A01 (Suppl. Fig. 2), indicate that the binding activity in batch A01 was likely due to a trace impurity that was removed during repurification.

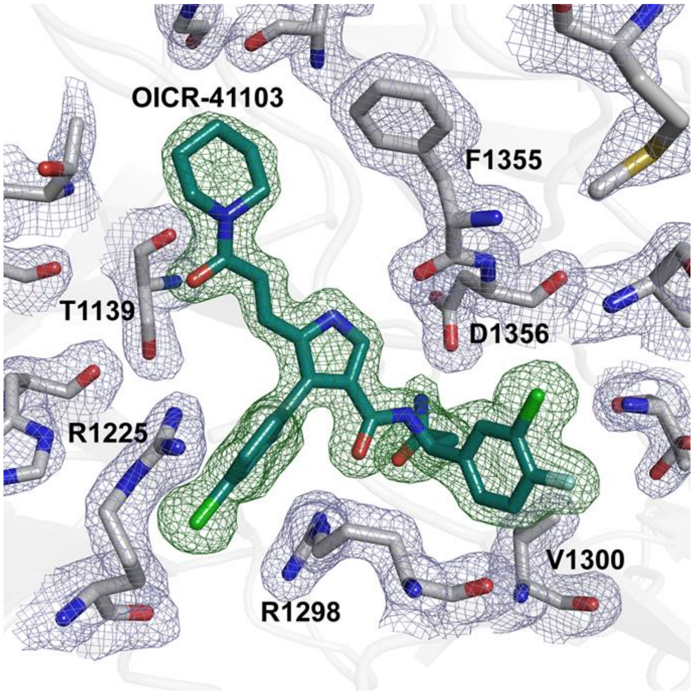

Supplementary Figure 4. Electron density map of OICR-41103 bound to the DCAF1 WDR domain central tunnel. The protein residues in the binding site are shown as sticks and the measured 2Fo-Fc electron density map around the residues in the vicinity of the compound is shown as blue mesh, contoured at 1.0σ level. OICR-41103 electron density omit map (Fo-Fc) is shown as green mesh contoured at 3σ level and the compound is rendered as deep teal sticks.

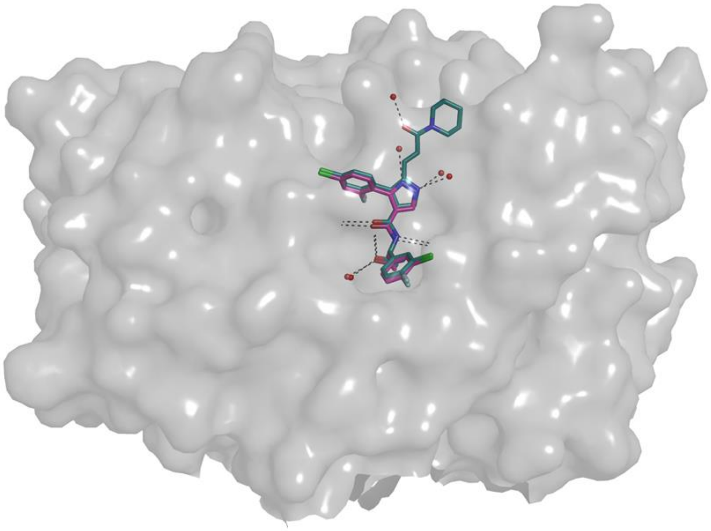

Supplementary Figure 5: The binding and location of OICR-8268 and OICR-41103 in the DCAF1 WDR domain central channel. A transparent surface representation of the DCAF1 WDR

domain in complex with OICR-8268 (magenta sticks) and the superimposed OICR-41103 ligand (deep teal sticks). OICR-41103 extends to the surface of the WDR doughnut central tunnel.

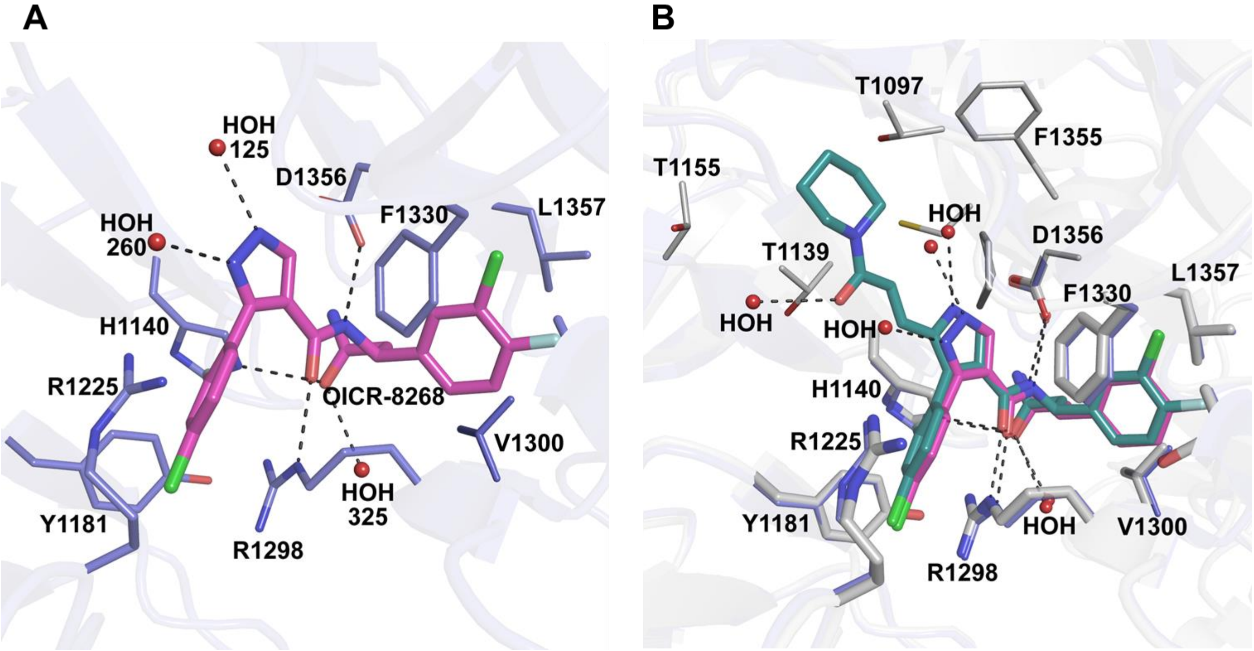

Supplementary Figure 6: DCAF1 WDR domain interactions with OICR-41103 compared to the parent compound, OICR-8268. (A) The binding site of the parent compound OICR-8268 (magenta sticks). **(B)** A superposition of the DCAF1-OICR-41103 and DCAF1-OICR-8268 (PDB 8f8e) showing how the binding of the two ligands compares.

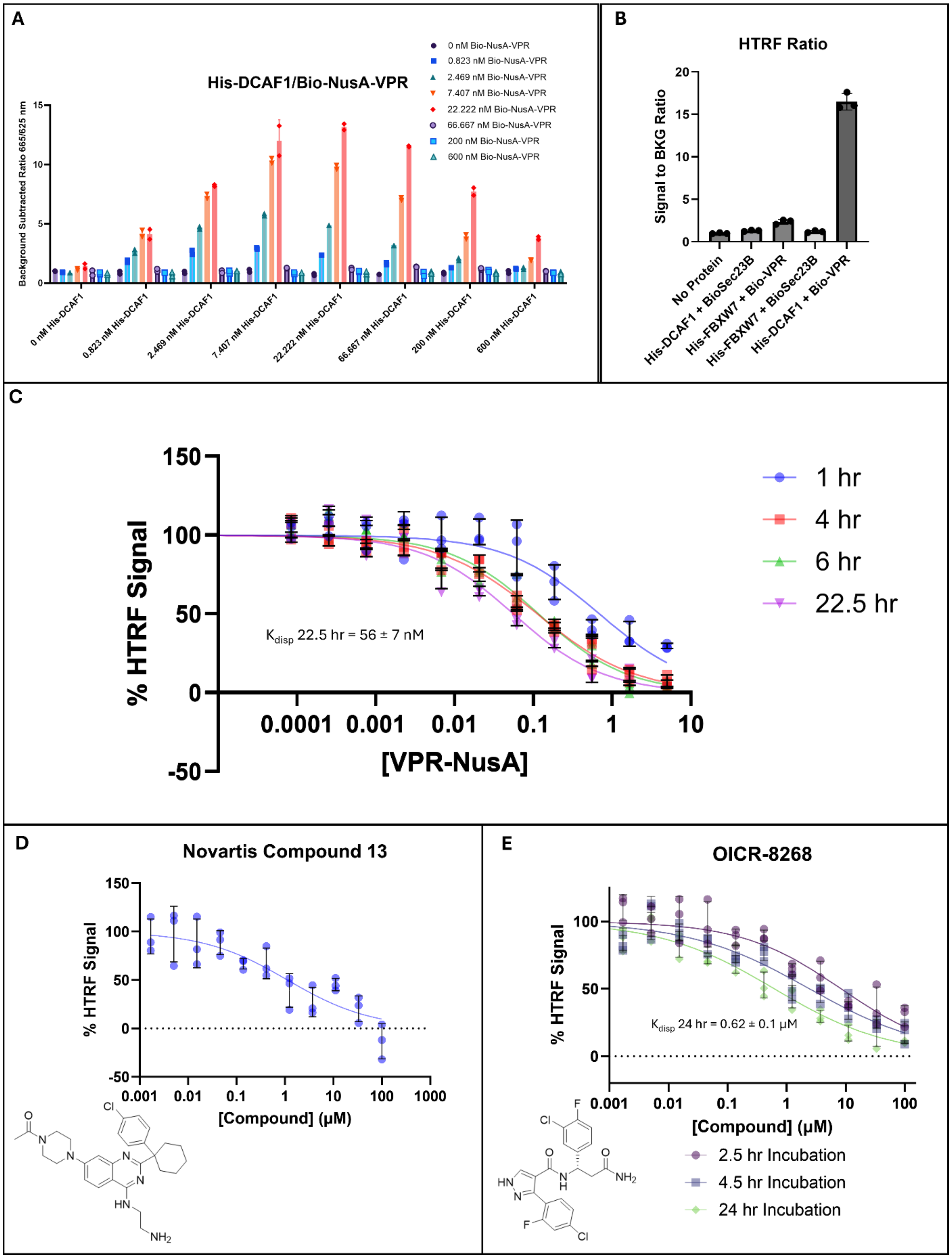
A
His-DCAF1/Bio-NusA-VPR
Background Subtracted Ratio 665/625 nm
0 nM Bio-NusA-VPR
0.823 nM Bio-NusA-VPR
2.469 nM Bio-NusA-VPR
7.407 nM Bio-NusA-VPR
22.222 nM Bio-NusA-VPR
66.667 nM Bio-NusA-VPR
200 nM Bio-NusA-VPR
600 nM Bio-NusA-VPR
0 nM His-DCAF1
0.823 nM His-DCAF1
2.469 nM His-DCAF1
7.407 nM His-DCAF1
22.222 nM His-DCAF1
66.667 nM His-DCAF1
200 nM His-DCAF1
600 nM His-DCAF1
B
HTRF Ratio
Signal to BKG Ratio
No Protein
His-DCAF1 + BioSec23B
His-FBXW7 + Bio-VPR
His-FBXW7 + BioSec23B
His-DCAF1 + Bio-VPR
C
% HTRF Signal
[VPR-NusA]
1 hr
4 hr
6 hr
22.5 hr
K$_{disp}$ 22.5 hr = 56 ± 7 nM
D
Novartis Compound 13
% HTRF Signal
[Compound] (μM)
E
OICR-8268
% HTRF Signal
K$_{disp}$ 24 hr = 0.62 ± 0.1 μM
[Compound] (μM)
2.5 hr Incubation
4.5 hr Incubation
24 hr Incubation

Supplementary Figure 7: HTRF assay demonstrates displacement of the full-length Vpr protein from the DCAF1 WDR domain (1038-1400) with non-biotinylated Vpr and previously published compound interacting with the central pocket of the DCAF1 WDR domain. A. Cross-titration experiment demonstrating protein concentrations required for optimal HTRF signal, n = 2 independent incubations. **B.** Substituting decoy proteins in place of DCAF1 or Vpr in the assay failed to produce a signal significantly greater than the background, demonstrating the signal in the assay is indeed specific to these two proteins. **C.** Loss of HTRF signal is apparent by the addition of increasing concentrations of the otherwise identical non-biotinylated full-length Vpr protein. Displacement of the Vpr protein and required overnight incubation at room temperature to witness maximal displacement of the biotinylated Vpr protein. Both His-DCAF1 and Bio-Vpr concentrations were held at 2.5 nM for displacement experiments. **D.** Compound reported to interact with the DCAF1 WDR pocket (PDB ID: 8OO5) demonstrated complete displacement of Vpr protein with K_{disp} around 1 µM after 24 hr incubation. **E.** Displacement of the DCAF1-Vpr interaction is observed as a reduction in HTRF signal *in vitro* by the originally published compound (OICR-8268) and approaches complete loss of signal. All points represent the average of three replicate incubations in separate wells, adjusted by subtracting the signal from blank wells containing compound without protein ± standard deviation. In all panels except panel A, the data represent the mean of three replicate incubations in separate wells, with values adjusted by subtracting the signal from blank wells containing compound without protein. Error bars indicate ± standard deviation.

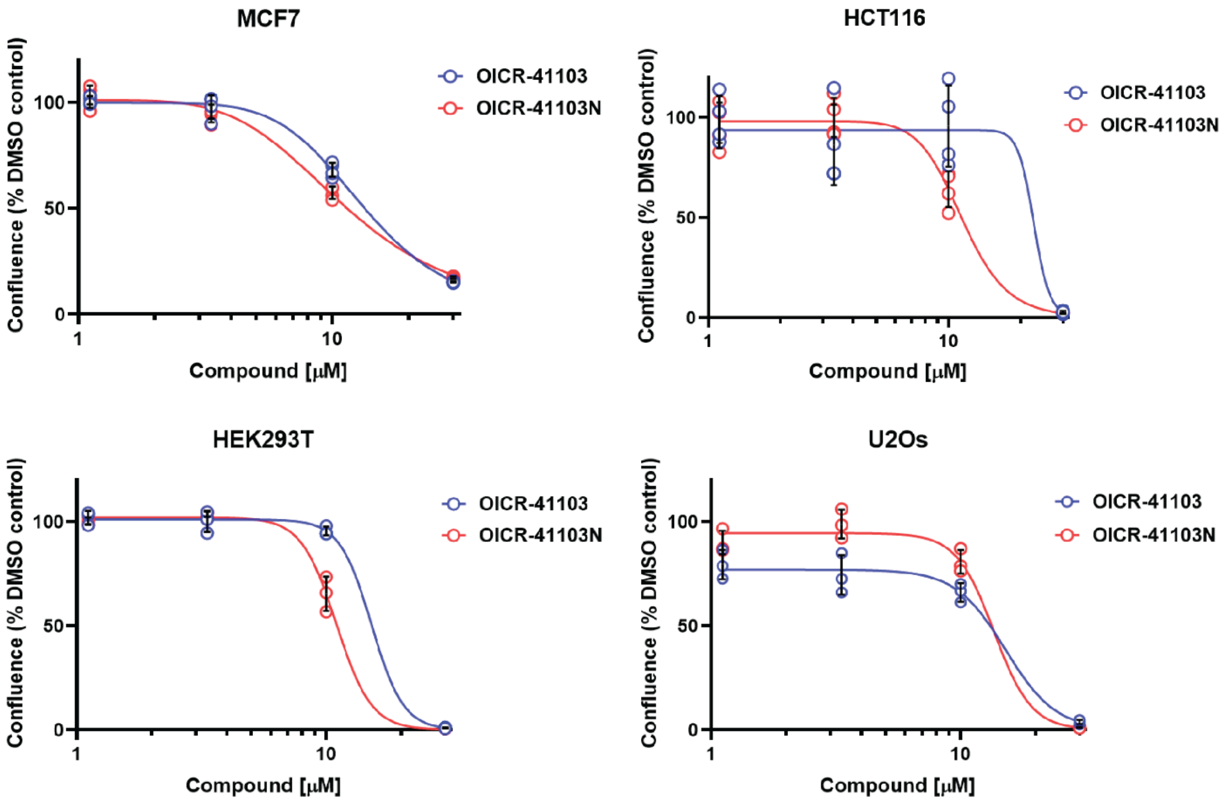

Supplementary Figure 8: OICR-41103 and OICR-41103N do not affect cell growth up to 3 µM. Cells were treated with indicated compounds concentrations for 5 days. The confluency was measured using IncuCyte™ ZOOM live cell imaging device. The results are MEAN+/-SEM, n=3-4 technical replicates.

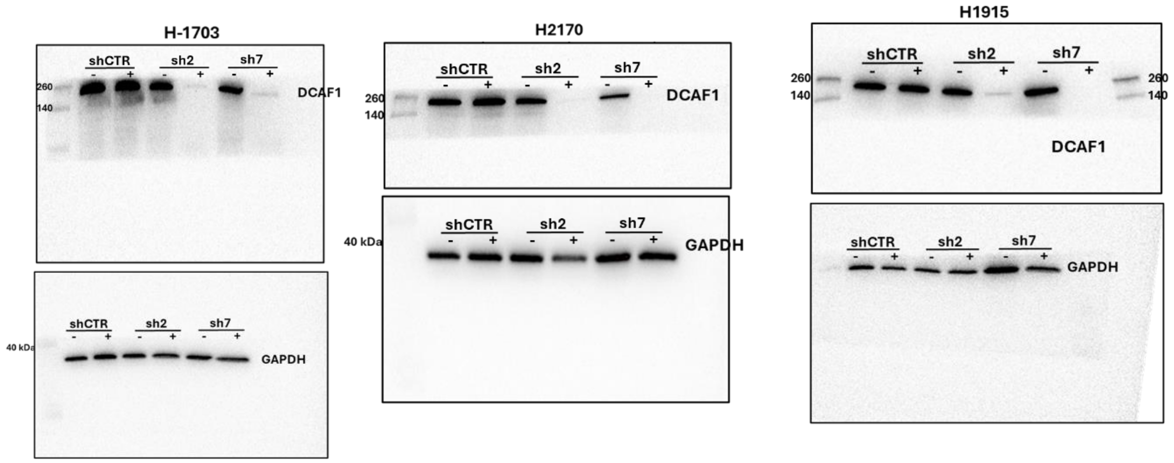

Supplementary Figure 9: DCAF1 protein levels following shRNA knockdown in NSCLC cell lines. Western blot analysis of DCAF1 protein expression in three non-small cell lung cancer (NSCLC) cell lines: H-1703, H2170, and H1915.

Chemistry Methods

All reagents were purchased from commercial vendors and used without further purification. The yields given refer to chromatographically purified and spectroscopically pure compounds, unless stated otherwise. Flash column chromatography was performed on a Teledyne ISCO CombiFlash Rf system using Teledyne ISCO RediSep Rf silica or RediSep Rf C18 cartidges as required. ^{1}H, ^{19}F and ^{13}C NMR spectra were recorded on a Bruker Avance-IV 400 MHz spectrometer at ambient temperature. Residual protons of DMSO-d_6 or Methanol-d_4 solvents were used as internal references. ^{1}H NMR spectral data are reported as follows: chemical shift (δ in ppm), multiplicity (br = broad, s = singlet, d = doublet, dd = doublet of doublets, t = triplet, m = multiplet), coupling constants (*J* in Hz) and proton integration. Compound purity was determined by UV absorbance at 254 nm during tandem liquid chromatography/mass spectrometry (LCMS) with a Waters Alliance H class UPLC system using an ACQUITY UPLC BEH-C18 column (1.7 µm, 2.1 mm x 50 mm) with a flow rate of 0.5 mL/min. Elution was carried out using water containing 0.1% formic acid + 2 mM NH_4OAc as mobile phase A and CH_3CN containing 0.1% formic acid as mobile phase B. Elution with a gradient of 2% B for 0.4 min, 2 to 65% B over 2.1 min, 65% B for 1 min, 65 to 95% B over 1.2 min then 2% B for 0.3 min. The purity of all compounds was > 95% by this method. Preparative HPLC for (**1**) and (**2**) was performed on a Shimadzu LC-20AP with a UV detector using a SUNFIRE C18 column (5 mm, 30 mm x 250 mm) with a flow rate of 26 mL/min. Elution was carried out using water containing 0.1% formic acid + 2 mM NH_4OAc as mobile phase A and CH_3CN as mobile phase B. For compound (**1**) the gradient used was 30 to 50% B over 30 min, 50% B for 9.0 min, 50 to 100% B over 2 min then 100 to 30% B over 5.0 min. For compound (**2**) the gradient used was 25 to 60% B over 24 min, 50% B for 4.0 min, 50 to 100% B over 2 min then 100 to 25% B over 6.0 min. Preparative HPLC for (**30**) and (**31**) was performed using a Waters CORTECS_T3 column (2.7 µm, 3 mm, 50mm). Elution was carried out using water containing 0.05% formic acid as mobile phase A and CH3CN containing 0.04% formic acid as mobile phase B. The gradient used was 1% to 99% B over 2.5 min, 99% B for 0.4 min. Optical rotations were measured on an Anton Paar MCP 200 polarimeter.

Synthesis of OICR-41103 (1)

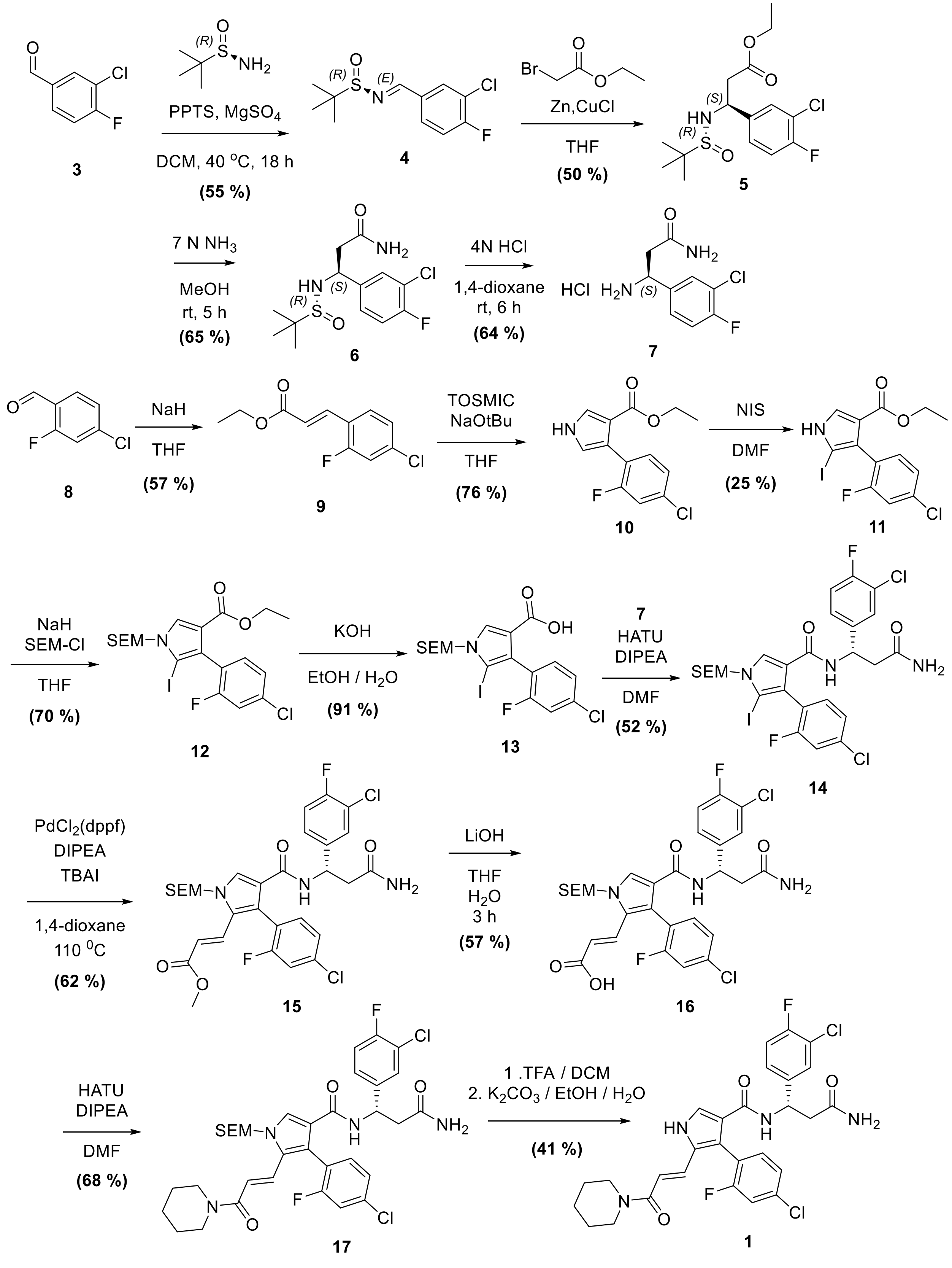

(R, E)-*N*-(3-chloro-4-fluorobenzylidene)-2-methylpropane-2-sulfinamide (**4**)

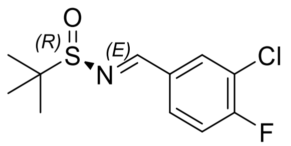

To a stirred solution of commercial 3-chloro-4-fluorobenzaldehyde (**3**) (5.0 g, 32 mmol, 1.0 eq.) in DCM (80 mL) were added PPTS (0.4 g, 1.6 mmol, 0.05 eq.), (*R*)-2-methylpropane-2-sulfinamide (3.9 g, 32 mmol, 1.3 eq.) and $MgSO_4$ (19 g, 160 mmol, 5.0 eq.). The resulting reaction mixture was heated at 40 ^oC for 18 h. The reaction mixture was filtered through a celite pad and concentrated under reduced pressure. The residue was purified by flash column chromatography on silica gel [10 to 20% EtOAc in hexanes] to afford (R, E)-*N*-(3-chloro-4-fluorobenzylidene)-2-methylpropane-2-sulfinamide (**4**) as a yellow oil.

Yield: 4.5 g (55%); ^1H NMR (400 MHz, DMSO-d_6) δ: 8.56 (s, 1H), 8.19 (br d, J = 7.3 Hz, 1H), 8.00 (br d, J = 6.1 Hz, 1H), 7.65 - 7.57 (m, 1H), 1.19 (s, 9H); ^{19}F NMR (377 MHz, DMSO-d_6) δ: -109.95; ^{13}C NMR (101 MHz, DMSO-d_6) δ: 160.78, 160.70, 158.26, 131.58, 131.58, 131.28, 130.18, 130.10, 120.71, 120.53, 117.84, 117.63, 57.40, 27.97, 24.13, 22.02; LRMS (ESI+) *m/z*: 262.2 $[M + H]^+$; HRMS (ESI+) m/z $[M+H]^+$ calcd for $C_{11}H_{14}ClFNOS$ 262.0469, found 262.0465; $[\alpha]^{25}_D$ = -45.4 (c = 0.10 g/100mL, MeOH).

ethyl (S)-3-(((R)-*tert*-butylsulfinyl)amino)-3-(3-chloro-4-fluorophenyl) propanoate (**5**)

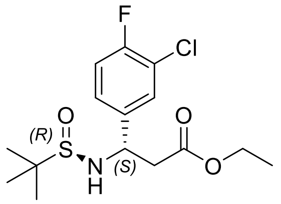

A stirred solution of zinc powder (7.4 g, 15 mmol, 10 eq.) and copper (I) chloride (0.11 g, 11 mmol, 0.1 eq.) in THF (80 mL) was heated at reflux for 30 min under nitrogen atmosphere. After cooling to room temperature, a solution of ethyl bromo acetate (4.7 g, 29 mmol, 2.5 eq.) in THF (40 mL) was added dropwise. The resulting reaction mixture was heated at 50 ^oC for 30 min. The reaction mixture was cooled to 0 ^oC and a solution of (R, E)-*N*-(3-chloro-4-fluorobenzylidene)-2-methylpropane-2-sulfinamide (**4**) (3.0 g, 11 mmol, 1.0 eq.) in THF (30 mL) was added dropwise *via* addition funnel. The resulting reaction mixture was stirred at 0 ^oC for 2 h. The reaction mixture was filtered through a celite pad, washing with EtOAc (50 mL). The filtrate was washed with water

(100 mL) and saturated brine (50 mL) before drying over sodium sulfate. The desiccant was removed by filtration and the filtrate was concentrated under reduced pressure. The residue was purified by flash column chromatography on silica gel [40 to 60% EtOAc in hexanes] to afford ethyl (*S*)-3-(((*R*)-*tert*-butylsulfinyl)amino)-3-(3-chloro-4-fluorophenyl) propanoate (**5**) as a yellow oil.

Yield: 2.0 g (50%); ^{1}H NMR (400 MHz, DMSO-d_6) δ: 7.59 (br d, J = 7.3 Hz, 1H), 7.37 (br d, J = 7.3 Hz, 2H), 5.66 (br d, J = 6.1 Hz, 1H), 4.73 - 4.54 (m, 1H), 4.06 - 3.95 (m, 2H), 3.06 - 2.95 (m, 1H), 2.77 (br dd, J = 7.3, 14.7 Hz, 1H), 1.17 (t, J = 7.3 Hz, 1H), 1.12 - 1.08 (m, 3H), 1.06 (s, 9H); ^{19}F NMR (377 MHz, DMSO-d_6) δ: -118.30; ^{13}C NMR (101 MHz, DMSO-d_6) δ : 169.87, 157.61, 155.16, 140.02, 139.98, 129.43, 128.09, 128.02, 119.22, 119.05, 116.80, 116.60, 60.16, 55.50, 55.26, 54.94, 42.31, 22.43, 13.96; LRMS (ESI+) *m/z*: 350.1 $[M + H]^+$; HRMS (ESI+) m/z $[M+H]^+$ calcd for $C_{15}H_{22}ClFNO_3S$ 350.0993, found 350.0987; $[\alpha]^{25}_D$ = -70.8 (c = 0.10 g/100mL, MeOH).

(S)-3-(((R)-*tert*-butylsulfinyl)amino)-3-(3-chloro-4-fluorophenyl) propanamide (**6**)

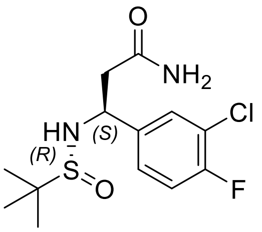

A solution of ethyl (*S*)-3-(((*R*)-*tert*-butylsulfinyl)amino)-3-(3-chloro-4-fluorophenyl) propanoate (**5**) (1.8 g, 5.3 mmol, 1.0 eq.) in 7N ammonia in MeOH (30 mL) was stirred at room temperature for 5 h. The volatiles were removed under reduced pressure and the residue was purified by flash column chromatography on silica gel [0 to 1% MeOH in DCM] to afford (*S*)-3-(((*R*)-*tert*-butylsulfinyl)amino)-3-(3-chloro-4-fluorophenyl) propanamide (**6**) as a yellow solid.

Yield: 1.2 g (71%); ^{1}H NMR (400 MHz, DMSO-d_6) δ: 7.53 (br d, J = 7.3 Hz, 1H), 7.44 - 7.31 (m, 3H), 6.93 (br s, 1H), 5.84 (br d, J = 3.1 Hz, 1H), 4.62 (br d, J = 6.1 Hz, 1H), 2.71 - 2.63 (m, 1H), 2.60 - 2.53 (m, 1H), 1.09 (br s, 9H); ^{19}F NMR (377 MHz, DMSO-d_6) δ: -118.53; ^{13}C NMR (101 MHz, DMSO-d_6) δ: 171.70, 157.53, 155.09, 140.34, 140.30, 129.38, 128.14, 128.06, 119.14, 118.96, 116.75, 116.54, 55.02, 55.02, 42.74, 22.43; LRMS (ESI+) *m/z*: 321.2 $[M + H]^+$; HRMS (ESI+) m/z $[M+H]^+$ calcd for $C_{13}H_{19}ClFN_2O_2S$ 321.0840, found 321.0831; $[\alpha]^{25}_D$ = -121.2 (c = 0.10 g/100mL, MeOH).

(S)-3-amino-3-(3-chloro-4-fluorophenyl)propanamide hydrochloride (**7**)

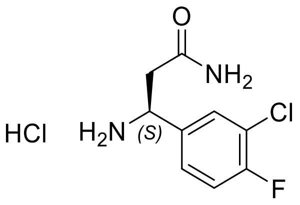

A solution of (*S*)-3-(((*R*)-*tert*-butylsulfinyl)amino)-3-(3-chloro-4-fluorophenyl)propanamide (**6**) (1.0 g, 4.7 mmol, 1.0 eq.) in 4M HCl in 1,4-dioxane (15 mL) was stirred at room temperature for 6 h. The volatiles were removed under reduced pressure. The residue was triturated using diethyl ether to afford (*S*)-3-amino-3-(3-chloro-4-fluorophenyl)propanamide hydrochloride (**7**) as a cream colored solid.

Yield: 0.6 g (64%); ^{1}H NMR (400 MHz, DMSO-d_6) δ: 8.51 (br s, 3H), 7.85 - 7.74 (m, 1H), 7.67 - 7.43 (m, 3H), 7.08 (br s, 1H), 4.71 - 4.53 (m, 1H), 2.86 - 2.70 (m, 2H); ^{19}F NMR (377 MHz, DMSO-d_6) δ: -116.56; ^{13}C NMR (101 MHz, DMSO-d_6) δ: 170.16, 158.29, 155.83, 135.17, 135.13, 130.17, 128.87, 128.79, 119.43, 119.26, 117.01, 116.79, 50.32, 25.66; LRMS (ESI+) *m/z*: 217.1 [M + H]$^+$; HRMS (ESI+) m/z [M+H]$^+$ calcd for $C_9H_{11}ClFN_2O$ 217.0544, found 217.0539; $[\alpha]^{25}_{D}$ = +7.0 (c = 0.10 g/100mL, MeOH).

ethyl (E)-3-(4-chloro-2-fluorophenyl)acrylate (**9**)

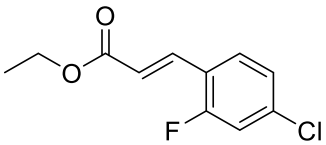

To a stirred solution of ethyl 2-(diethoxyphosphoryl)acetate (7.7 g, 35 mmol, 1.1 eq.) in THF (30 mL) was added NaH (60% in mineral oil) (1.5 g, 38 mmol , 1.2 eq.) portion wise at 0 ^{o}C under nitrogen atmosphere. After stirring at room temperature for 30 minutes, the reaction was cooled back to 0 ^{o}C and a solution of commercial 4-chloro-2-fluorobenzaldehyde (**8**) (5.0 g, 32 mmol, 1.0 eq.) in THF (20 mL) was added dropwise. The resulting reaction mixture was stirred at room temperature for 2 h. The reaction mixture was poured onto water (200 mL) and extracted with EtOAc (2 x 200 mL). The combined organic layers were dried over sodium sulfate. The desiccant was removed by filtration and the filtrate was concentrated under reduced pressure. The residue was purified by flash column chromatography on silica gel [10 to 20% EtOAc in hexanes) to afford (*E*)-3-(4-chloro-2-fluorophenyl) acrylate (**9**) as a white solid.

Yield: 4.5 g, (57%); ^{1}H NMR (400 MHz, DMSO- d_6) δ: 7.98 - 7.89 (m, 1H), 7.65 (d, J = 16.5 Hz, 1H), 7.57 (br d, J = 10.4 Hz, 1H), 7.37 (br d, J = 8.5 Hz, 1H), 6.72 (d, J = 16.5 Hz, 1H), 4.25 - 4.16 (m, 2H), 1.26 (t, J = 7.3 Hz, 3H); ^{19}F NMR (377 MHz, DMSO- d_6) δ: -113.30 (s, 1F); ^{13}C NMR (101 MHz, DMSO-d_6) δ: 165.69, 161.53, 159.00, 135.81, 135.69, 134.87, 134.87, 130.43, 130.43, 125.30, 125.27, 121.31, 121.25, 120.84, 120.73, 116.79, 116.53, 60.28, 14.06; LRMS (ESI+) *m/z*: 229.0 [M + H]$^+$; HRMS (ESI+) m/z [M+H]$^+$ calcd for $C_{11}H_{11}ClFO_2$ 229.0432, found 229.0424.

ethyl 4-(4-chloro-2-fluorophenyl)-1H-pyrrole-3-carboxylate (**10**)

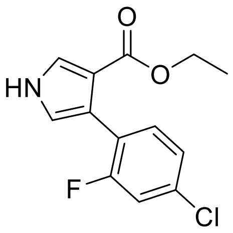

To a stirred solution of ethyl (*E*)-3-(4-chloro-2-fluorophenyl) acrylate (**9**) (4.5 g, 20 mmol, 1.0 eq.) and 1-((isocyanomethyl)sulfonyl)-4-methylbenzene (3.8 g, 20 mmol, 1.0 eq.) in THF (70 mL) was added sodium *tert*-butoxide (2.0 g, 22 mmol, 1.1 eq.) at -40 °C under nitrogen atmosphere. The reaction mixture was stirred at -40 °C for 2 h and then allowed to come to room temperature for 3 h. The reaction mixture was poured into water (200 mL) and extracted with EtOAc (2 x 300 mL). The combined organic layers were dried over sodium sulfate. The desiccant was removed by filtration and the filtrate was concentrated under reduced pressure. The residue was purified by flash column chromatography on silica gel [20 to 30% EtOAc in hexanes) to afford ethyl 4-(4-chloro-2-fluorophenyl)-1*H*-pyrrole-3-carboxylate (**10**) as a yellow solid.

Yield 4.0 g (76%); MS: ES+ 268.1; ^{1}H NMR (400 MHz, DMSO- d_6) δ: 11.67 (br s, 1H), 7.51 - 7.47 (m, 1H), 7.40 - 7.32 (m, 2H), 7.27 - 7.22 (m, 1H), 6.95 (d, J = 2.4 Hz, 1H), 4.13 - 4.00 (m, 2H), 1.18 - 1.07 (m, 3H); ^{19}F NMR (377 MHz, DMSO- d_6) δ: -111.09 (s, 1F); ^{13}C NMR (101 MHz, DMSO- d_6) δ: 163.75, 160.77, 158.30, 132.64, 132.60, 131.51, 131.41, 124.99, 123.75, 123.75, 122.68, 122.52, 119.84, 119.84, 116.73, 115.58, 115.31, 113.52, 58.74, 14.06; LRMS (ESI+) *m/z*: 268.1 [M + H]$^+$; HRMS (ESI+) m/z [M+H]$^+$ calcd for $C_{13}H_{12}ClFNO_2$ 268.0541, found 268.0536.

ethyl 4-(4-chloro-2-fluorophenyl)-5-iodo-1H-pyrrole-3-carboxylate (**11**)

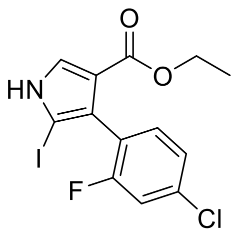

To a solution of ethyl 4-(4-chloro-2-fluorophenyl)-1*H*-pyrrole-3-carboxylate (**10**) (4.0 g, 15 mmol, 1.0 eq.) in DMF (160 mL) was added NIS (4.3 g, 20 mmol, 1.3 eq.) at room temperature under nitrogen atmosphere. The resulting reaction mixture was stirred for 3 h. The reaction mixture was poured into cold-water (500 mL) and extracted with EtOAc (2 x 200 mL). The combined organic layers were dried over sodium sulfate. The desiccant was removed by filtration and the filtrate was concentrated under reduced pressure. The residue was purified by flash column chromatography on silica gel [0.5 to 0.7% EtOAc in hexanes] to yield ethyl 4-(4-chloro-2-fluorophenyl)-5-iodo-1*H*-pyrrole-3-carboxylate (**11**) as a yellow solid.

NOTE: The bis-iodinated product, ethyl 4-(4-chloro-2-fluorophenyl)-2,5-diiodo-1*H*-pyrrole-3-carboxylate was also isolated in significant quantity and can be converted into the desired mono-iodinated product to improve the throughput of the synthetic sequence.

Yield 1.5 g (25%); ^{1}H NMR (400 MHz, DMSO- d_6) δ: 12.27 (br s, 1H), 7.63 (s, 1H), 7.42 (br d, J = 9.8 Hz, 1H), 7.37 - 7.18 (m, 2H), 4.01 (br s, 2H), 1.06 (t, J = 6.7 Hz, 3H); ^{19}F NMR (377 MHz, DMSO- d_6) δ: -110.08 (s, 1F); ^{13}C NMR (101 MHz, DMSO- d_6) δ: 162.46, 160.99, 158.53, 133.67, 133.63, 132.52, 132.42, 128.30, 123.87, 123.87, 122.72, 122.56, 122.38, 115.78, 115.56, 115.52, 72.86, 59.01, 13.98; LRMS (ESI+) *m/z*: 394.2 $[M + H]^+$; HRMS (ESI+) m/z $[M+H]^+$ calcd for $C_{13}H_{11}ClFINO_2$ 393.9507, found 393.9496.

ethyl 4-(4-chloro-2-fluorophenyl)-5-iodo-1-((2-(trimethylsilyl) ethoxy) methyl)-1H-pyrrole-3-carboxylate (**12**)

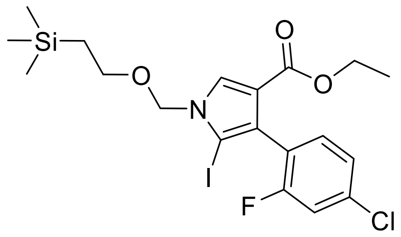

To a stirred solution of ethyl 4-(4-chloro-2-fluorophenyl)-5-iodo-1*H*-pyrrole-3-carboxylate (**11**) (1.5 g, 3.8 mmol, 1.0 eq.) in THF (20 mL) sodium hydride (60% in mineral oil) (0.23 g,

5.7 mmol, 1.5 eq.) was added portion wise at 0 °C under nitrogen atmosphere. The reaction mixture was stirred at room temperature for 30 min. After cooling back down to 0 °C, SEM-Cl (0.76 g, 4.6 mol, 1.2 eq.) was added and the resulting reaction mixture was stirred at room temperature for 2 h. The reaction mixture was poured into water (200 mL) and extracted with EtOAc (2 x 100 mL). The combined organic layers were dried over anhydrous sodium sulfate. The desiccant was removed by filtration and the filtrate was concentrated under reduced pressure. The residue was purified by flash column chromatography on silica gel [2 to 3 % EtOAc in hexanes] to afford ethyl 4-(4-chloro-2-fluorophenyl)-5-iodo-1-((2-(trimethylsilyl) ethoxy) methyl)-1*H*-pyrrole-3-carboxylate (**12**) as a brown solid.

Yield 1.4 g (70%); ^{1}H NMR (400 MHz, DMSO- d_6) δ: 8.04 (s, 1H), 7.44 (br d, J = 9.8 Hz, 1H), 7.36 - 7.29 (m, 1H), 7.29 - 7.19 (m, 1H), 5.34 (br s, 2H), 4.07 - 3.96 (m, 2H), 3.54 (br t, J = 7.9 Hz, 2H), 1.07 (br t, J = 7.3 Hz, 3H), 0.89 - 0.80 (m, 2H), -0.03 (s, 9H); ^{19}F NMR (377 MHz, DMSO-d_6) δ: -110.25 (s, 1F); ^{13}C NMR (101 MHz, DMSO- d_6) δ: 162.32, 160.90, 158.43, 133.59, 133.55, 132.78, 132.68, 131.35, 124.27, 123.92, 123.92, 122.78, 115.83, 115.79, 115.53, 78.97, 65.29, 59.18, 17.03, 13.90, -1.35; LRMS (ESI+) *m/z*: 524.3 $[M + H]^+$; HRMS (ESI+) m/z $[M+H]^+$ calcd for $C_{19}H_{25}ClFINO_3Si$ 524.0321, found 524.0320.

4-(4-chloro-2-fluorophenyl)-5-iodo-1-((2-(trimethylsilyl) ethoxy) methyl)-1H-pyrrole-3-carboxylic acid (**13**)

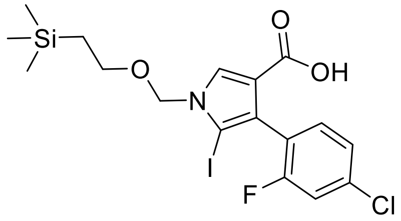

To a stirred solution of ethyl 4-(4-chloro-2-fluorophenyl)-5-iodo-1-((2-(trimethylsilyl)ethoxy)methyl)-1*H*-pyrrole-3-carboxylate (**12**) (1.4 g, 2.7 mmol, 1.0 eq.) in ethanol (14 mL) was added a solution of KOH (7.5 g, 130 mmol, 50.0 eq.) in water (14 mL) dropwise at rt. The resulting reaction mixture was heated to 100 °C for 2 h. The volatiles were removed under reduced pressure and the resultant was diluted with cold water (100 mL) then acidified with aqueous HCl. The product was then extracted with EtOAc (2 x 500 mL). The combined organic layers were dried over sodium sulfate. The desiccant was removed by filtration and the filtrate was concentrated under reduced pressure. The residue was purified by flash column chromatography on silica gel [40 to 50% EtOAc in hexanes] to yield 4-(4-chloro-2-

fluorophenyl)-5-iodo-1-((2-(trimethylsilyl)ethoxy)methyl)-1*H*-pyrrole-3-carboxylic acid (**13**) as a brown oil.

Yield 1.2 g, (91%); ^{1}H NMR (400 MHz, DMSO-d_6) δ ppm: 11.95 (br s, 1H), 7.99 - 7.93 (m, 1H), 7.46 - 7.39 (m, 1H), 7.34 - 7.28 (m, 1H), 7.27 - 7.19 (m, 1H), 5.33 (br d, J = 2.4 Hz, 2H), 3.54 (br t, J = 7.9 Hz, 2H), 0.85 (br t, J = 7.9 Hz, 2H), 0.01 - -0.08 (m, 9H); ^{19}F NMR (377 MHz, DMSO-d_6) δ: -110.01 (br s, 1F); ^{13}C NMR (101 MHz, DMSO-d_6) δ: 163.85, 160.90, 158.44, 133.64, 133.60, 132.63, 132.53, 131.39, 124.44, 123.87, 123.87, 123.02, 122.87, 116.79, 115.81, 115.55, 79.51, 78.94, 65.27, 40.18, 17.07, -1.34; LRMS (ESI+) *m/z*: 496.1 [M + H]$^{+}$; HRMS (ESI+) m/z [M+H]$^{+}$ calcd for $C_{17}H_{21}ClFINO_3Si$ 496.0008, found 496.0001.

(S)-N-(3-amino-1-(3-chloro-4-fluorophenyl)-3-oxopropyl)-4-(4-chloro-2-fluorophenyl)-5-iodo-1-((2-(trimethylsilyl)ethoxy)methyl)-1H-pyrrole-3-carboxamide (**14**)

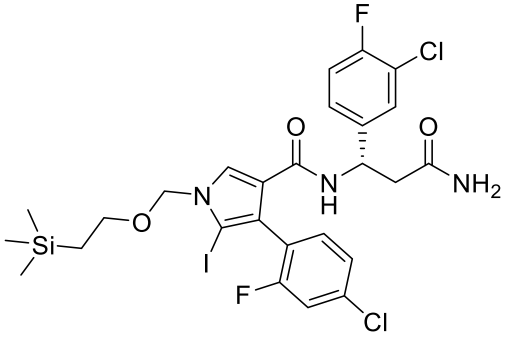

To a stirred solution of 4-(4-chloro-2-fluorophenyl)-5-iodo-1-((2-(trimethylsilyl)ethoxy)methyl)-1*H*-pyrrole-3-carboxylic acid (**13**) (1.1 g, 2.2 mmol, 1.0 eq.) and (*S*)-3-amino-3-(3-chloro-4-fluorophenyl)propanamide hydrochloride (**7**) (0.73 g, 2.9 mmol, 1.3 eq.) in DMF (12 mL) were added HATU (1.3 g, 3.3 mmol, 1.5 eq.) and DIPEA (0.86 g, 6.7 mmol, 3.0 eq.) at rt. The resulting reaction mixture was stirred at rt for 3 h. The reaction mixture was poured into water (100 mL) and extracted with EtOAc (2 X 100 mL). The combined organic layers were dried over sodium sulfate. The desiccant was removed by filtration and the filtrate was concentrated under reduced pressure. The residue was purified by flash column chromatography on silica gel [70 to 100% EtOAc in hexanes] to yield (*S*)-*N*-(3-amino-1-(3-chloro-4-fluorophenyl)-3-oxopropyl)-4-(4-chloro-2-fluorophenyl)-5-iodo-1-((2-(trimethylsilyl)ethoxy)methyl)-1*H*-pyrrole-3-carboxamide (**14**) as an off white solid.

Yield 0.8 g (52%); ^{1}H NMR (400 MHz, DMSO-d_6) δ ppm: 8.24 - 8.17 (m, 1H), 7.93 (br s, 1H), 7.49 - 7.14 (m, 8H), 6.83 (br s, 1H), 5.32 (br s, 2H), 5.22 - 5.12 (m, 1H), 3.57 (br t, J = 7.9 Hz,

2H), 0.92 - 0.82 (m, 2H), -0.02 (s, 9H); ^{19}F NMR (377 MHz, DMSO-d_6) δ: -109.77 (s, 1F), -119.33 (br s, 1F); ^{13}C NMR (101 MHz, DMSO-d_6) δ: 171.26, 171.21, 161.74, 158.31, 157.22, 154.78, 141.06, 141.06, 133.62, 133.58, 132.46, 132.35, 128.44, 127.34, 127.34, 127.24, 123.92, 123.86, 123.86, 123.57, 123.03, 122.87, 120.42, 118.98, 118.81, 116.50, 116.30, 115.83, 115.57, 79.34, 78.98, 65.38, 48.92, 41.40, 40.18, 17.14, -1.29; LRMS (ESI+) *m/z*: 694.4 [M + H]$^+$; HRMS (ESI+) m/z [M+H]$^+$ calcd for $C_{26}H_{29}Cl_2F_2IN_3O_3Si$ 694.0368, found 694.0362; $[\alpha]^{25}_D$ = +0.8 (c = 0.10 g/100mL, MeOH).

methyl (S, E)-3-(4-((3-amino-1-(3-chloro-4-fluorophenyl)-3-oxopropyl)carbamoyl)-3-(4-chloro-2-fluorophenyl)-1-((2-(trimethylsilyl)ethoxy)methyl)-1H-pyrrol-2-yl)acrylate (**15**)

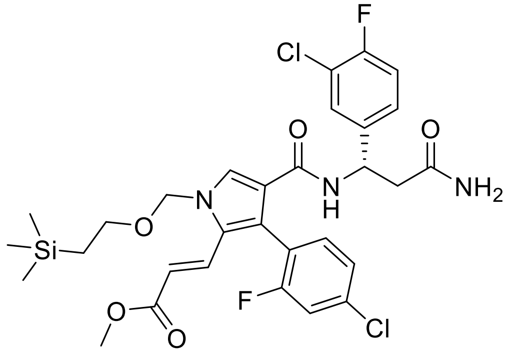

To a stirred solution of (*S*)-*N*-(3-amino-1-(3-chloro-4-fluorophenyl)-3-oxopropyl)-4-(4-chloro-2-fluorophenyl)-5-iodo-1-((2-(trimethylsilyl)ethoxy)methyl)-1*H*-pyrrole-3-carboxamide (**14**) (5.0 g, 7.2 mmol, 1.0 eq.) in 1,4 dioxane (50 mL) were added DIPEA (50 mL) and TBAI (4.0 g, 3.0 mmol , 1.5 eq.) at room temperature under nitrogen atmosphere. The resulting reaction mixture was degassed with nitrogen atmosphere for 15 min and then ($PdCl_2$(dppf)) (0.52 g, 0.70 mmol, 0.1 eq.) and methyl acrylate (1.8 g, 22 mmol, 3.0 eq.) were added at room temperature. The reaction mixture was heated at 110 °C for 48 h. The reaction mixture was poured into water (500 mL) and extracted with EtOAc (2 x 500 mL). The combined organic layers were dried over anhydrous sodium sulfate. The desiccant was removed by filtration and the filtrate was concentrated under reduced pressure. The residue was purified by reverse phase flash column chromatography [C18 column; 40 to 45% MeCN in water] to afford methyl (*S*,*E*)-3-(4-((3-amino-1-(3-chloro-4-fluorophenyl)-3-oxopropyl)carbamoyl)-3-(4-chloro-2-fluorophenyl)-1-((2-(trimethylsilyl)ethoxy)methyl)-1*H*-pyrrol-2-yl)acrylate (**15**) as a brown oil.

Yield 2.9 g (62%); ^{1}H NMR (400 MHz, Methanol-d_4) δ ppm: 7.69 (s, 1H), 7.48 (d, J = 16.2 Hz, 1H), 7.36 (dd, J = 1.8, 7.0 Hz, 1H), 7.29 - 7.11 (m, 5H), 6.01 (d, J = 16.2 Hz, 1H), 5.47 (s, 2H),

5.33 - 5.26 (m, 1H), 3.69 (s, 3H), 3.68 - 3.63 (m, 2H), 2.74 - 2.61 (m, 2H), 1.01 - 0.88 (m, 2H), 0.00 (s, 9H); ^{19}F NMR (377 MHz, DMSO-d_6) δ: -110.31 (br s, 1F), -119.27 (s, 1F); ^{13}C NMR (101 MHz, DMSO-d_6) δ: 171.18, 166.69, 161.79, 160.69, 158.22, 157.26, 154.83, 140.93, 140.93, 133.37, 133.33, 133.13, 133.03, 131.15, 129.12, 128.46, 127.36, 127.28, 126.38, 124.42, 124.39, 122.02, 121.35, 121.19, 119.03, 118.98, 118.85, 116.55, 116.34, 116.21, 115.95, 115.15, 76.85, 65.47, 51.41, 48.96, 41.36, 17.06, -1.37; LRMS (ESI+) *m/z*: 652.4 $[M + H]^+$; HRMS (ESI+) m/z $[M+H]^+$ calcd for $C_{30}H_{34}Cl_2F_2N_3O_5Si$ 652.1613, found 652.1617; $[\alpha]^{25}_D$ = +17.0 (c = 0.10 g/100mL, MeOH).

(S,E)-3-(4-((3-amino-1-(3-chloro-4-fluorophenyl)-3-oxopropyl)carbamoyl)-3-(4-chloro-2-fluorophenyl)-1-((2-(trimethylsilyl)ethoxy)methyl)-1H-pyrrol-2-yl)acrylic acid (**16**)

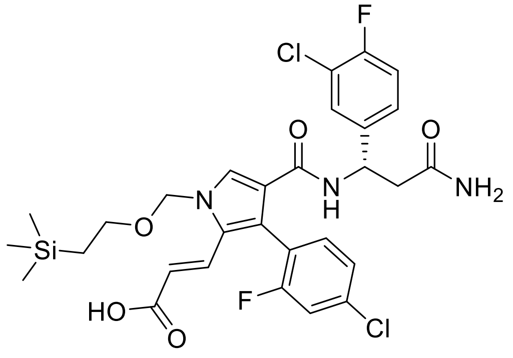

To a solution of methyl (*S*,*E*)-3-(4-((3-amino-1-(3-chloro-4-fluorophenyl)-3-oxopropyl)carbamoyl)-3-(4-chloro-2-fluorophenyl)-1-((2-(trimethylsilyl)ethoxy)methyl)-1*H*-pyrrol-2-yl)acrylate (**15**) (2.8 g, 4.3 mmol, 1.0 eq.) in THF (28 mL), water (14 mL) and MeOH (28 mL) was added lithium hydroxide monohydrate (0.9 g, 22 mmol, 5.0 eq) at room temperature. The reaction mixture was stirred for 3 h. The reaction mixture was poured into water (100 mL) and the aqueous layer washed with EtOAc (2 x 200 mL) before it was acidified with aqueous hydrochloric acid. The product was then extracted with EtOAc (3 x 300 mL). The combined organic layers were dried over anhydrous sodium sulfate. The desiccant was removed by filtration and the filtrate was concentrated under reduced pressure. The residue was purified by reverse phase flash column chromatography [C18 column; 40 to 45% MeCN in water + 0.1% formic acid] to afford (*S*,*E*)-3-(4-((3-amino-1-(3-chloro-4-fluorophenyl)-3-oxopropyl)carbamoyl)-3-(4-chloro-2-fluorophenyl)-1-((2-(trimethylsilyl)ethoxy)methyl)-1*H*-pyrrol-2-yl)acrylic acid (**16**) as a colourless solid.

Yield 1.5 g (57%); ^{1}H NMR (400 MHz, Methanol-d_4) δ ppm: 7.66 (s, 1H), 7.43 (d, J = 16.2 Hz, 1H), 7.37 (dd, J = 1.8, 7.0 Hz, 1H), 7.32 - 7.09 (m, 5H), 6.00 (d, J = 16.2 Hz, 1H), 5.46 (s, 2H), 5.33 - 5.25 (m, 1H), 3.70 - 3.61 (m, 2H), 2.75 - 2.60 (m, 2H), 1.01 - 0.90 (m, 2H), 0.00 (s, 8H); ^{19}F NMR (377 MHz, DMSO-d_6) δ: -110.29 (br s, 1F), -119.39 (s, 1F); ^{13}C NMR (101 MHz, DMSO-d_6) δ: 171.24, 161.98, 158.31, 141.08, 141.08, 133.44, 133.40, 132.80, 132.69, 128.45, 128.37, 127.31, 127.24, 127.10, 124.21, 121.59, 119.00, 118.83, 118.71, 116.50, 116.30, 115.80, 76.66, 65.36, 48.99, 41.44, 17.09, -1.38; LRMS (ESI+) *m/z*: 638.5 [M + H]$^+$; HRMS (ESI+) m/z [M+H]$^+$ calcd for $C_{29}H_{32}Cl_2F_2N_3O_5Si$ 638.1456, found 638.1451; $[\alpha]^{25}_D$ = +18.0 (c = 0.10 g/100mL, MeOH).

(S,E)-N-(3-amino-1-(3-chloro-4-fluorophenyl)-3-oxopropyl)-4-(4-chloro-2-fluorophenyl)-5-(3-oxo-3-(piperidin-1-yl)prop-1-en-1-yl)-1-((2-(trimethylsilyl)ethoxy)methyl)-1H-pyrrole-3-carboxamide (**17**)

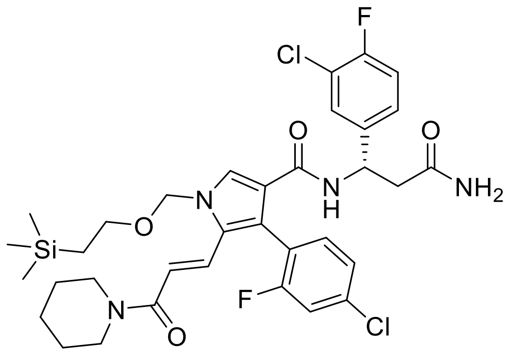

To a stirred solution of (*S*,*E*)-3-(4-((3-amino-1-(3-chloro-4-fluorophenyl)-3-oxopropyl)carbamoyl)-3-(4-chloro-2-fluorophenyl)-1-((2-(trimethylsilyl)ethoxy)methyl)-1*H*-pyrrol-2-yl)acrylic acid (**16**) (1.0 g, 1.6 mmol, 1.0 eq.) in DMF (6 mL) were added HATU (0.89 g, 2.3 mmol, 1.5 eq.) and TEA (0.47 mL, 4.7 mmol, 3.0 eq) under nitrogen atmosphere. The resulting reaction mixture was stirred at room temperature for 10 min before piperidine (0.16 g, 1.1 mmol, 1.9 eq.) was added. After stirring at room temperature for 2 h the reaction mixture was poured into ice cold water (100 mL) and extracted with EtOAc (3 x 50 mL). The combined organic layers were dried over anhydrous sodium sulfate. The desiccant was removed by filtration and the filtrate was concentrated under reduced pressure. The residue was purified reverse phase flash chromatography [C18 column; 40 to 45% MeCN in water + 0.1% formic acid] to afford to yield (*S*,*E*)-*N*-(3-amino-1-(3-chloro-4-fluorophenyl)-3-oxopropyl)-4-(4-chloro-2-fluorophenyl)-5-(3-oxo-3-(piperidin-1-yl)prop-1-en-1-yl)-1-((2-(trimethylsilyl)ethoxy)methyl)-1*H*-pyrrole-3-carboxamide (**17**) as a near colourless solid.

Yield 0.75 g (68%); ^{1}H NMR (400 MHz, DMSO-d_6) δ ppm: 8.25 (br d, J = 7.9 Hz, 1H), 7.82 (s, 1H), 7.48 - 7.40 (m, 2H), 7.38 - 7.17 (m, 6H), 6.83 (br s, 1H), 6.41 (d, J = 15.9 Hz, 1H), 5.46 (s, 2H), 5.24 - 5.16 (m, 1H), 3.61 (t, J = 7.9 Hz, 2H), 3.49 - 3.39 (m, 2H), 3.27 - 3.14 (m, 2H), 2.54 (br d, J = 7.3 Hz, 2H), 1.62 - 1.53 (m, 2H), 1.40 (br d, J = 0.6 Hz, 4H), 0.92 - 0.85 (m, 2H), -0.02 (s, 9H); ^{19}F NMR (377 MHz, DMSO-d_6) δ: -110.15 (br s, 1F), -119.30 (s, 1F); ^{13}C NMR (101 MHz, DMSO-d_6) δ: 171.74, 164.27, 162.50, 161.26, 158.80, 157.76, 155.32, 141.49, 141.49, 134.06, 134.02, 133.35, 133.24, 128.96, 128.50, 128.35, 128.35, 127.82, 127.74, 124.81, 124.81, 122.57, 122.41, 120.43, 119.53, 119.36, 119.21, 117.30, 117.00, 116.80, 116.60, 116.34, 77.23, 65.84, 49.46, 46.40, 43.07, 41.87, 26.84, 26.84, 25.85, 24.53, 17.64, -0.86; LRMS (ESI+) *m/z*: 705.4 $[M + H]^+$; HRMS (ESI+) m/z $[M+H]^+$ calcd for $C_{34}H_{41}Cl_2F_2N_4O_4Si$ 705.2242, found 705.2232; $[\alpha]^{25}_D$ = +9.2 (c = 0.10 g/100mL, MeOH).

(S,E)-3-(((4-(4-chloro-2-fluorophenyl)-5-(3-oxo-3-(piperidin-1-yl)prop-1-en-1-yl)-1H-pyrrol-3-yl)(l3-oxidaneylidene)methyl)amino)-3-(3-chloro-4-fluorophenyl)propanamide (**1**) OICR-41103

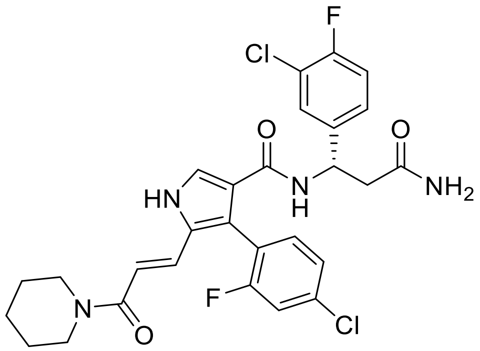

To a stirred solution of (*S*,*E*)-*N*-(3-amino-1-(3-chloro-4-fluorophenyl)-3-oxopropyl)-4-(4-chloro-2-fluorophenyl)-5-(3-oxo-3-(piperidin-1-yl)prop-1-en-1-yl)-1-((2-(trimethylsilyl)ethoxy)methyl)-1*H*-pyrrole-3-carboxamide (**17**) (0.75 g, 1.1 mmol, 1.0 eq.) in DCM (10 mL) was added TFA (5 mL) at room temperature. The reaction mixture was stirred at rt for 16 h. The volatiles were removed under reduced pressure and the residue was dissolved in EtOH (10 mL) and water (5 mL). Potassium carbonate (1.0 g, 7.4 mmol, 7.0 eq.) was added and the mixture was stirred at room temperature for 16 h. The reaction mixture was filtered through a pad of celite eluting with DCM (30 mL) and filtrate was concentrated under reduce pressure. The residue was purified by preparative HPLC to afford (S,E)-N-(3-amino-1-(3-chloro-4-fluorophenyl)-3-oxopropyl)-4-(4-chloro-2-fluorophenyl)-5-(3-oxo-3-(piperidin-1-yl)prop-1-en-1-yl)-1H-pyrrole-3-carboxamide (**1**) as a colourless solid.

Yield 0.25 g (41%); ^{1}H NMR (400 MHz, DMSO-d_6) δ ppm: 12.02 (br s, 1H), 8.18 (d, J = 7.9 Hz, 1H), 7.69 (s, 1H), 7.47 (dd, J = 2.1, 7.3 Hz, 1H), 7.40 - 7.25 (m, 4H), 7.23 - 7.17 (m, 1H), 7.06 - 6.92 (m, 2H), 6.84 (br s, 1H), 5.29 - 5.16 (m, 1H), 3.62 - 3.41 (m, 4H), 2.55 (br d, J = 7.3 Hz, 2H), 1.68 - 1.57 (m, 2H), 1.56 - 1.37 (m, 4H); ^{19}F NMR (377 MHz, DMSO-d_6) δ: -110.05 (br s, 1F), -119.38 (br s, 1F); ^{13}C NMR (101 MHz, DMSO-d_6) δ: 171.28, 164.00, 162.47, 160.94, 158.47, 157.23, 154.80, 141.22, 141.18, 133.63, 133.63, 132.46, 132.35, 128.86, 128.51, 127.90, 127.34, 127.27, 123.88, 123.88, 123.69, 121.49, 121.33, 119.49, 119.45, 119.00, 118.83, 116.54, 116.34, 115.88, 115.61, 113.76, 48.95, 45.92, 42.59, 42.54, 42.54, 41.58, 38.95, 26.50, 26.50, 25.44, 25.44, 24.17; LRMS (ESI+) *m/z*: 575.0 $[M + H]^+$; HRMS (ESI+) m/z $[M+H]^+$ calcd for $C_{28}H_{27}Cl_2F_2N_4O_3$ 575.1428, found 575.1416; $[\alpha]^{25}_{D}$ = +33.0 (c = 0.10 g/100mL, MeOH).

Synthesis of OICR-41103N (2)

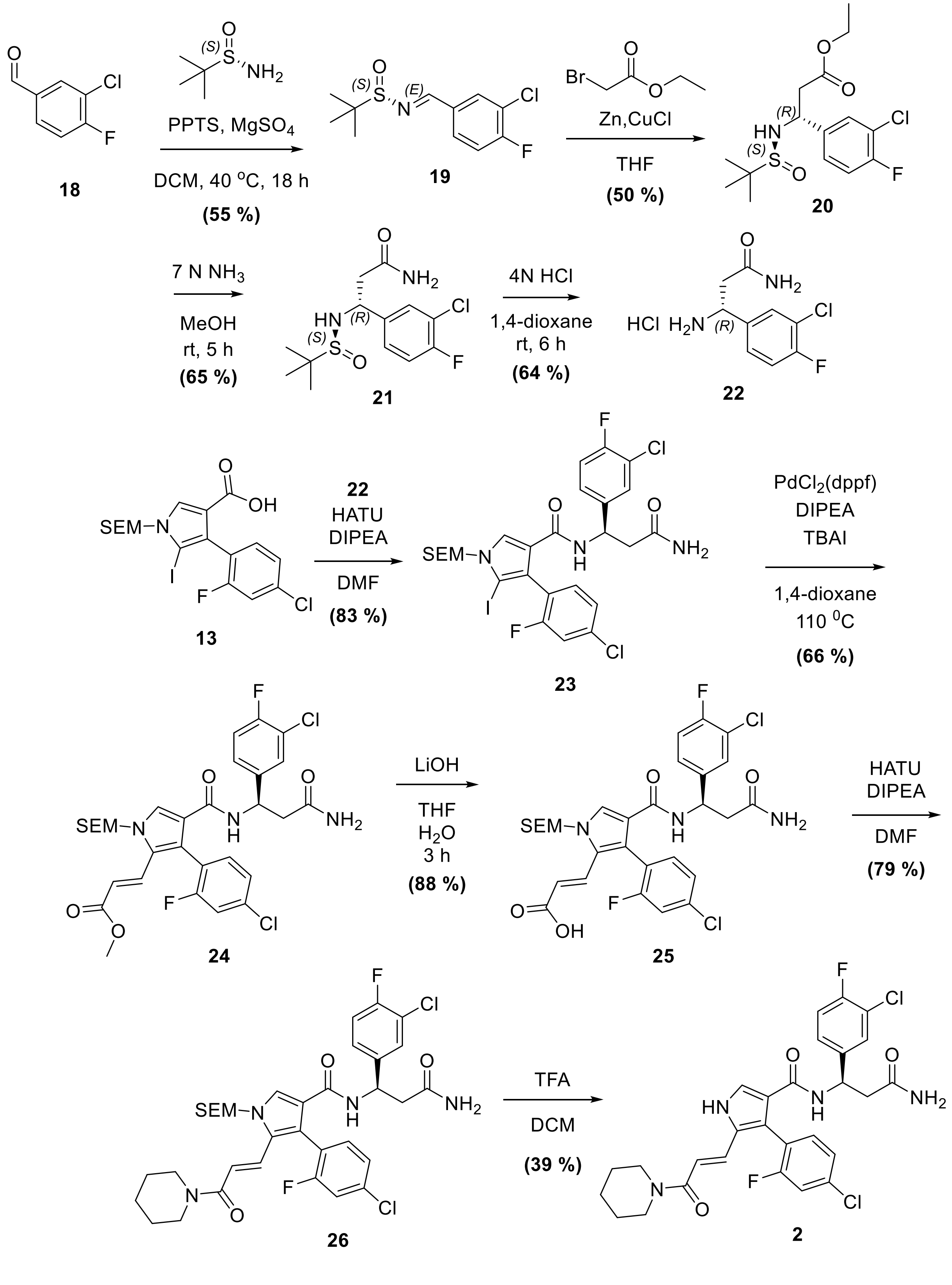

(S, E)-*N*-(3-chloro-4-fluorobenzylidene)-2-methylpropane-2-sulfinamide (**19**)

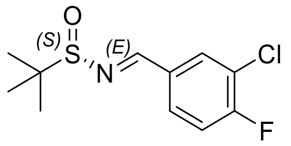

The title compound was prepared according to the procedure described for compound **4** substituting (*S*)-2-methylpropane-2-sulfinamide for (*R*)-2-methylpropane-2-sulfinamide.

Yield: 4.5 g (55%); ^{1}H NMR (400 MHz, DMSO-d_6) δ: 8.56 (s, 1H), 8.18 (dd, J = 1.8, 7.3 Hz, 1H), 8.04 - 7.96 (m, 1H), 7.65 - 7.57 (m, 1H), 1.18 (s, 9H); ^{19}F NMR (377 MHz, DMSO-d_6) δ: -109.95; ^{13}C NMR (101 MHz, DMSO-d_6) δ: 160.73, 158.27, 131.61, 131.61, 131.33, 130.21, 130.12, 120.72, 120.54, 117.89, 117.67, 59.59, 57.43, 47.37, 27.98, 24.14, 22.04; LRMS (ESI+) *m/z*: 262.2 $[M + H]^+$; HRMS (ESI+) m/z $[M+H]^+$ calcd for $C_{11}H_{14}ClFNOS$ 262.0469, found 262.0463; $[\alpha]^{25}_D$ = +2.7 (c = 1.0 g/100mL, MeOH).

ethyl (R)-3-(((S)-tert-butylsulfinyl)amino)-3-(3-chloro-4-fluorophenyl)propanoate (**20**)

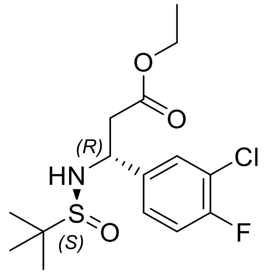

The title compound was prepared according to the procedure described for compound **5** substituting compound **19** for compound **4**.

Yield: 2.0 g (50%); ^{1}H NMR (400 MHz, DMSO-d_6) δ: 7.59 (br d, J = 7.9 Hz, 1H), 7.37 (br d, J = 7.9 Hz, 2H), 5.67 (br d, J = 6.1 Hz, 1H), 4.65 (q, J = 6.7 Hz, 1H), 4.09 - 3.95 (m, 2H), 3.00 (dd, J = 6.7, 15.9 Hz, 1H), 2.77 (dd, J = 7.9, 15.3 Hz, 1H), 1.13 - 1.08 (m, 3H), 1.06 (s, 9H); ^{19}F NMR (377 MHz, DMSO-d_6) δ: -118.31 (s, 1F); ^{13}C NMR (101 MHz, DMSO-d_6) δ: 169.82, 157.58, 155.13, 139.97, 139.97, 129.39, 128.05, 127.97, 119.20, 119.02, 116.75, 116.54, 60.10, 55.46, 55.21, 42.28, 22.38, 13.90; LRMS (ESI+) *m/z*: 350.3 $[M + H]^+$; HRMS (ESI+) m/z $[M+H]^+$ calcd for $C_{15}H_{22}ClFNO_3S$ 350.0993, found 350.0986; $[\alpha]^{25}_D$ = +31.2 (c = 0.10 g/100mL, MeOH).

(R)-3-(((S)-tert-butylsulfinyl)amino)-3-(3-chloro-4-fluorophenyl)propanamide (**21**)

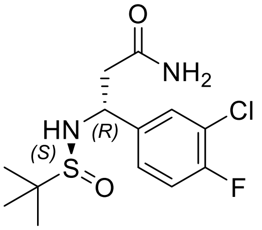

The title compound was prepared according to the procedure described for compound **6** substituting compound **20** for compound **5**.

Yield: 1.1 g (65%); ^{1}H NMR (400 MHz, DMSO-d_6) δ: 7.57 - 7.50 (m, 1H), 7.47 - 7.31 (m, 3H), 6.93 (br s, 1H), 5.84 (br d, J = 4.3 Hz, 1H), 4.67 - 4.57 (m, 1H), 2.72 - 2.63 (m, 1H), 2.61 - 2.52 (m, 1H), 1.09 (s, 9H); ^{19}F NMR (377 MHz, DMSO-d_6) δ: -118.53 (s, 1F); ^{13}C NMR (101 MHz, DMSO-d_6) δ: 171.68, 157.52, 155.07, 140.30, 140.30, 129.35, 128.10, 128.03, 119.12, 118.94, 116.70, 116.50, 54.99, 54.99, 42.71, 22.40; LRMS (ESI+) *m/z*: 321.1 $[M + H]^+$; HRMS (ESI+) m/z $[M+H]^+$ calcd for $C_{13}H_{19}ClFN_2O_2S$ 321.0840, found 321.0831; $[\alpha]^{25}_D$ = +92.8 (c = 0.10 g/100mL, MeOH).

(R)-3-amino-3-(3-chloro-4-fluorophenyl)propanamide hydrochloride(**22**)

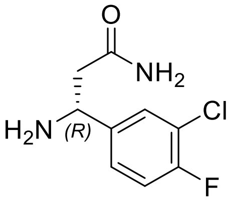

The title compound was prepared according to the procedure described for compound **7** substituting compound **21** for compound **6**.

Yield: 0.5 g (63%); ^{1}H NMR (400 MHz, DMSO-d_6) δ: 8.26 (s, 1H), 7.65 - 7.59 (m, 1H), 7.45 - 7.36 (m, 3H), 6.88 (br s, 1H), 4.31 (br t, J = 6.7 Hz, 1H), 2.47 - 2.43 (m, 2H); ^{19}F NMR (377 MHz, DMSO-d_6) δ: -118.84 (s, 1F); ^{13}C NMR (101 MHz, DMSO-d_6) δ: 171.48, 164.65, 157.57, 155.12, 140.52, 129.03, 127.71, 127.64, 119.15, 118.97, 116.67, 116.46, 51.01, 42.38; LRMS (ESI+) *m/z*: 217.1 $[M + H]^+$; HRMS (ESI+) m/z $[M+H]^+$ calcd for $C_9H_{11}ClFN_2O$ 217.0544, found 217.0538; $[\alpha]^{25}_D$ = -0.4 (c = 0.50 g/100mL, MeOH).

(R)-N-(3-amino-1-(3-chloro-4-fluorophenyl)-3-oxopropyl)-4-(4-chloro-2-fluorophenyl)-5-iodo-1-((2-(trimethylsilyl)ethoxy)methyl)-1H-pyrrole-3-carboxamide (**23**)

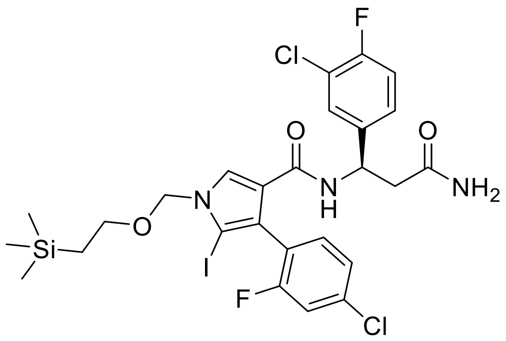

The title compound was prepared according to the procedure described for compound **14** substituting compound **22** for compound **7**.

Yield: 5.8 g (83%); ^{1}H NMR (400 MHz, DMSO-d_6) δ: 8.20 (br d, J = 7.9 Hz, 1H), 7.93 (s, 1H), 7.46 - 7.42 (m, 1H), 7.36 - 7.17 (m, 6H), 6.83 (br s, 1H), 5.33 (s, 2H), 5.23 - 5.15 (m, 1H), 3.58 (t, J = 7.9 Hz, 2H), 2.54 (br s, 2H), 0.92 - 0.85 (m, 2H), 0.00 (s, 9H); ^{19}F NMR (377 MHz, DMSO-d_6) δ: -109.77 (s, 1F), -119.36 (br s, 1F); ^{13}C NMR (101 MHz, DMSO-d_6) δ: 171.19, 161.73, 160.76, 158.28, 157.21, 154.78, 141.06, 141.02, 133.61, 133.57, 132.45, 132.35, 128.42, 127.32, 127.31, 127.29, 127.23, 123.85, 123.85, 123.56, 123.02, 122.87, 120.42, 118.97, 118.79, 116.49, 116.28, 115.82, 115.55, 79.30, 78.98, 65.38, 48.91, 41.38, 17.13, -1.30; LRMS (ESI+) *m/z*: 694.4 $[M + H]^+$; HRMS (ESI+) m/z $[M+H]^+$ calcd for $C_{26}H_{29}Cl_2F_2IN_3O_3Si$ 694.0368, found 694.0355; $[\alpha]^{25}_D$ = -3.8 (c = 0.10 g/100mL, MeOH).

methyl (R,E)-3-(4-((3-amino-1-(3-chloro-4-fluorophenyl)-3-oxopropyl)carbamoyl)-3-(4-chloro-2-fluorophenyl)-1-((2-(trimethylsilyl)ethoxy)methyl)-1H-pyrrol-2-yl)acrylate (**24**)

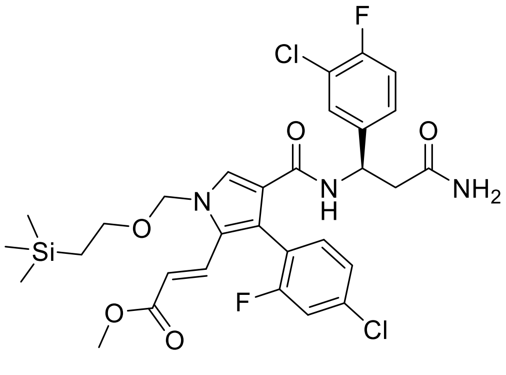

The title compound was prepared according to the procedure described for compound **15** substituting compound **23** for compound **14**.

Yield: 3.6 g (66%); ^{1}H NMR (400 MHz, DMSO-d_6) δ: 8.29 (br d, J = 7.9 Hz, 1H), 7.89 (s, 1H), 7.43 (br d, J = 8.5 Hz, 2H), 7.38 - 7.15 (m, 6H), 6.84 (br s, 1H), 5.89 - 5.79 (m, 1H), 5.48 (s, 2H), 5.25 - 5.13 (m, 1H), 3.68 - 3.54 (m, 5H), 2.54 (br s, 2H), 0.93 - 0.81 (m, 2H), 0.00 - -0.07 (m, 9H); ^{19}F NMR (377 MHz, DMSO-d_6) δ: -110.31 (br s, 1F), -119.27 (s, 1F); ^{13}C NMR (101 MHz, DMSO-d_6) δ: 171.16, 166.64, 161.77, 160.67, 158.20, 157.23, 154.80, 140.88, 140.88, 133.35, 133.31, 133.10, 133.00, 131.12, 129.07, 128.43, 127.30, 127.23, 126.36, 124.36, 124.36, 121.93, 121.33, 121.16, 118.99, 118.82, 116.49, 116.29, 116.16, 115.90, 115.15, 76.82, 65.45, 51.35, 48.94, 41.30, 17.06, -1.42; LRMS (ESI+) *m/z*: 652.0 [M + H]$^+$; HRMS (ESI+) m/z [M+H]$^+$ calcd for $C_{30}H_{34}Cl_2F_2N_3O_5Si$ 652.1613, found 652.1606; $[\alpha]^{25}_D$ = -12.0 (c = 0.10 g/100mL, MeOH).

(R,E)-3-(4-((3-amino-1-(3-chloro-4-fluorophenyl)-3-oxopropyl)carbamoyl)-3-(4-chloro-2-fluorophenyl)-1-((2-(trimethylsilyl)ethoxy)methyl)-1H-pyrrol-2-yl)acrylic acid (**25**)

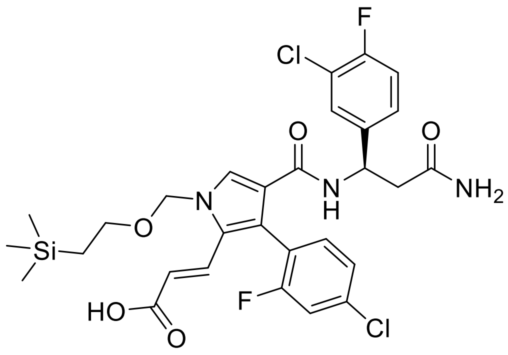

The title compound was prepared according to the procedure described for compound **16** substituting compound **24** for compound **15**.

Yield: 3.0 g (88%); ^{1}H NMR (400 MHz, DMSO-d_6) δ: 8.27 (br d, J = 8.5 Hz, 1H), 7.86 (s, 1H), 7.48 - 7.17 (m, 8H), 6.83 (br s, 1H), 5.79 (br d, J = 15.3 Hz, 1H), 5.46 (br s, 2H), 5.24 - 5.15 (m, 1H), 3.63 - 3.56 (m, 2H), 2.54 (br s, 2H), 0.88 (br t, J = 7.9 Hz, 2H), -0.04 (s, 9H); ^{19}F NMR (377 MHz, DMSO-d_6) δ: -110.30 (br s, 1F), -119.29 (s, 1F); ^{13}C NMR (101 MHz, DMSO-d_6) δ: 171.18, 169.13, 167.64, 161.86, 160.71, 158.24, 157.23, 154.79, 140.94, 140.91, 133.38, 133.37, 133.35, 132.97, 132.86, 130.32, 128.70, 128.43, 127.30, 127.22, 126.65, 124.32, 124.28, 121.49, 121.31, 121.31, 119.00, 118.84, 118.84, 117.41, 117.35, 116.49, 116.28, 116.11, 115.85, 76.75, 65.40, 48.94, 41.34, 38.97, 17.06, -1.39; LRMS (ESI+) *m/z*: 638.5 [M + H]$^+$; HRMS (ESI+) m/z [M+H]$^+$ calcd for $C_{29}H_{32}Cl_2F_2N_3O_5Si$ 638.1456, found 638.1462; $[\alpha]^{25}_D$ = -11.6 (c = 0.10 g/100mL, MeOH).

(R,E)-N-(3-amino-1-(3-chloro-4-fluorophenyl)-3-oxopropyl)-4-(4-chloro-2-fluorophenyl)-5-(3-oxo-3-(piperidin-1-yl)prop-1-en-1-yl)-1-((2-(trimethylsilyl)ethoxy)methyl)-1H-pyrrole-3-carboxamide (**26**)

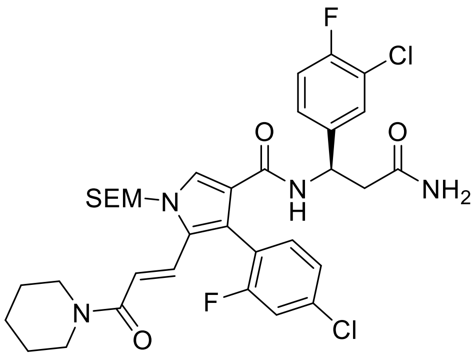

The title compound was prepared according to the procedure described for compound **17** substituting compound **25** for compound **16**.

Yield: 0.8 g (79%); ^{1}H NMR (400 MHz, DMSO-d_6) δ: 8.25 (br d, J = 8.5 Hz, 1H), 7.82 (s, 1H), 7.47 - 7.38 (m, 2H), 7.37 - 7.16 (m, 6H), 6.83 (br s, 1H), 6.40 (br d, J = 15.9 Hz, 1H), 5.45 (s, 2H), 5.20 (q, J = 7.3 Hz, 1H), 3.59 (br t, J = 7.9 Hz, 2H), 3.43 (br s, 1H), 3.20 (br s, 2H), 1.56 (br d, J = 4.3 Hz, 2H), 1.39 (br s, 4H), 0.94 - 0.83 (m, 2H), -0.03 (s, 9H); ^{19}F NMR (377 MHz, DMSO-d_6) δ: -110.15 (br s, 1F), -119.31 (s, 1F); ^{13}C NMR (101 MHz, DMSO-d_6) δ: 171.18, 163.72, 161.95, 160.72, 158.26, 157.21, 154.77, 140.98, 140.94, 133.51, 133.47, 132.79, 132.69, 128.42, 127.96, 127.54 (dd, J = 7.8, 56.7 Hz, 1C), 124.27, 124.27, 122.03, 121.86, 119.93, 118.98, 118.81, 118.63, 116.38 (br dd, J = 21.5, 42.1 Hz, 1C), 115.81, 76.68, 65.29, 48.91, 45.83, 42.48, 41.33, 26.32, 25.29, 23.98, 17.08, -1.40; LRMS (ESI+) *m/z*: 705.6 [M + H]$^+$; HRMS (ESI+) m/z [M+H]$^+$ calcd for $C_{34}H_{41}Cl_2F_2N_4O_4Si$ 705.2242, found 705.2251; $[\alpha]^{25}_D$ = -6.2 (c = 0.10 g/100mL, MeOH).

(R,E)-N-(3-amino-1-(3-chloro-4-fluorophenyl)-3-oxopropyl)-4-(4-chloro-2-fluorophenyl)-5-(3-oxo-3-(piperidin-1-yl)prop-1-en-1-yl)-1H-pyrrole-3-carboxamide (**2**) OICR-41103N

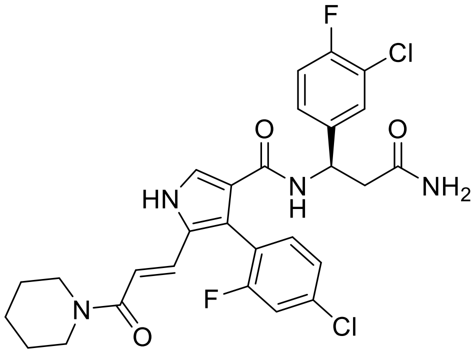

The title compound was prepared according to the procedure described for compound **1** substituting compound **26** for compound **17**.

Yield: 0.24 g (39%); ^{1}H NMR (400 MHz, DMSO-*d*$_6$) δ: 12.02 (br d, J = 0.6 Hz, 1H), 8.19 (d, J = 7.9 Hz, 1H), 7.69 (d, J = 2.4 Hz, 1H), 7.49 - 7.45 (m, 1H), 7.40 - 7.24 (m, 4H), 7.23 - 7.17 (m, 1H), 7.06 - 6.92 (m, 2H), 6.84 (br s, 1H), 5.30 - 5.16 (m, 1H), 3.61 - 3.43 (m, 4H), 2.55 (br d, J = 7.0 Hz, 2H), 1.61 (br d, J = 4.9 Hz, 2H), 1.56 - 1.39 (m, 4H); ^{19}F NMR (377 MHz, DMSO-*d*$_6$) δ: -110.05 (br d, J = 2.8 Hz, 1F), -119.40 (s, 1F); ^{13}C NMR (101 MHz, DMSO-*d*$_6$) δ: 171.23, 163.97, 162.43, 160.89, 158.43, 157.20, 154.76, 141.16, 141.12, 133.62, 133.58, 132.42, 132.32, 128.82, 128.47, 127.86, 127.29, 127.22, 123.89, 123.85, 123.62, 121.44, 121.29, 119.48, 119.37, 118.96, 118.78, 116.49, 116.28, 115.84, 115.57, 113.76, 48.90, 45.89, 42.51, 41.52, 26.43, 25.39, 24.12; LRMS (ESI+) *m/z*: 575.1 $[M + H]^+$; HRMS (ESI+) m/z $[M+H]^+$ calcd for $C_{28}H_{27}Cl_2F_2N_4O_3$ 575.1428, found 575.1424; $[\alpha]^{25}_{D}$ = -32.8 (c = 0.10 g/100mL, MeOH).

Synthesis of DCAF1 BODIPY tracer based on Novartis ligand (31)

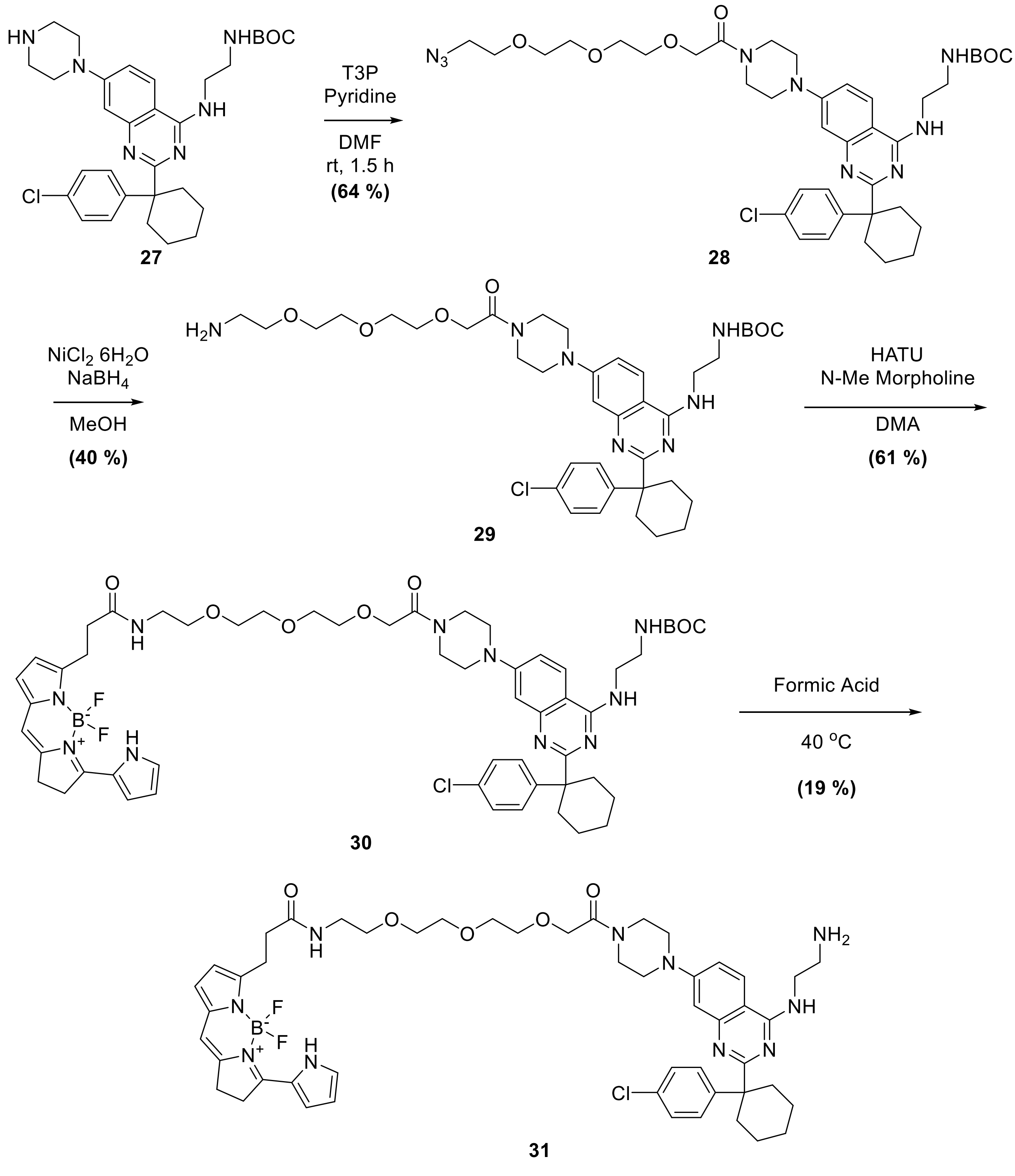

tert-butyl N-[2-({7-[4-(2-{2-[2-(2-azidoethoxy)ethoxy]ethoxy}acetyl)piperazin-1-yl]-2-[1-(4-chlorophenyl)cyclohexyl]quinazolin-4-yl}amino)ethyl]carbamate (**28**)

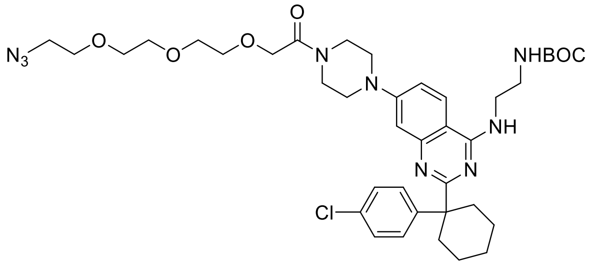

To a solution of tert-butyl N-[2-({2-[1-(4-chlorophenyl)cyclohexyl]-7-(piperazin-1-yl)quinazolin-4-yl}amino)ethyl]carbamate (0.10 g, 0.17 mmol, 1.0 eq.) and 11-Azido-3,6,9-trioxaundecanoic acid (0.041 g; 0.17 mmol, 1.0 eq) in DMF (3 mL) were added pyridine (0.14 mL, 1.7 mmol, 10 eq.) and T3P solution (50% in DMF, 0.60 mL, 1.01 mmol, 6.0 eq.). The reaction was stirred at room temperature for 1.5 h. The volatiles were removed under reduced pressure and the residue was partitioned between DCM and water. The layers were separated and the aqueous layer was extracted with additional DCM. The combined organic layers were concentrated to dryness and the residue was purified by flash column chromatography on silica gel [0 to 30% MeOH in DCM] to yield tert-butyl N-[2-({7-[4-(2-{2-[2-(2-azidoethoxy)ethoxy]ethoxy}acetyl)piperazin-1-yl]-2-[1-(4-chlorophenyl)cyclohexyl]quinazolin-4-yl}amino)ethyl]carbamate (**28**).

Yield: 0.088 g (64%); LRMS (ESI+) *m/z*: 780.2 $[M + H]^+$.

tert-butyl N-[2-({7-[4-(2-{2-[2-(2-aminoethoxy)ethoxy]ethoxy}acetyl)piperazin-1-yl]-2-[1-(4-chlorophenyl)cyclohexyl]quinazolin-4-yl}amino)ethyl]carbamate (**29**)

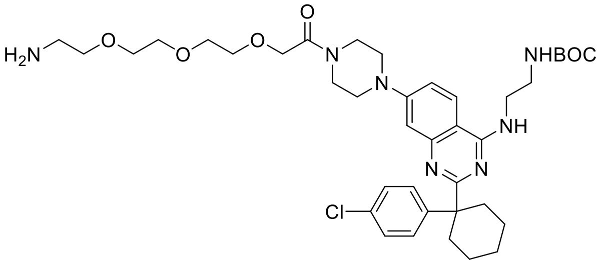

tert-butyl N-[2-({7-[4-(2-{2-[2-(2-azidoethoxy)ethoxy]ethoxy}acetyl)piperazin-1-yl]-2-[1-(4-chlorophenyl)cyclohexyl]quinazolin-4-yl}amino)ethyl]carbamate (**28**) (0.088 g, 0.11 mmol, 1.0 eq.) was dissolved in MeOH (6.5 mL). The solution was sonicated for 5 min and then reaction vessel was flushed with argon gas for 5 min. Nickel(II) chloride (0.0013 g, 0.01 mmol, 0.1 eq.) and sodium borohydride (0.010 g, 0.27 mmol, 2.5 eq.) were added and the solution was stirred at room temperarture for 3 h protected from light. Incomplete conversion was observed, additional

portions of nickel(II) chloride (0.0013 g, 0.01 mmol, 0.1 eq.) and sodium borohydride (0.0104 g, 0.27 mmol, 2.5 eq.) were added and the reaction was stirred for an additional 2 h at room temperarture protected from light. The solvent was partially evaporated under reduced pressure and the reaction mixture was diluted with water. The resulting precipitate was collected by filtration, washed with water and dried to afford tert-butyl N-[2-({7-[4-(2-{2-[2-(2-aminoethoxy)ethoxy]ethoxy}acetyl)piperazin-1-yl]-2-[1-(4-chlorophenyl)cyclohexyl]quinazolin-4-yl}amino)ethyl]carbamate (**29**) as an off-white solid that was used without further purification.

Yield: 0.041 g (39%); LRMS (ESI+) *m/z*: 377.6 $[M/2 + H]^+$.

N-(2-(2-(2-(2-(4-(4-((2-(((λ^1-methyl)(λ^1-oxidaneyl)boraneyl)amino)ethyl)amino)-2-(1-(4-chlorophenyl)cyclohexyl)quinazolin-7-yl)piperazin-1-yl)-2-oxoethoxy)ethoxy)ethoxy)ethyl)-3-(5,5-difluoro-3-(1H-pyrrol-2-yl)-2,5-dihydro-1H-4λ^4,5λ^4-dipyrrolo[1,2-c:2',1'-f][1,3,2]diazaborinin-7-yl)propanamide (**30**)

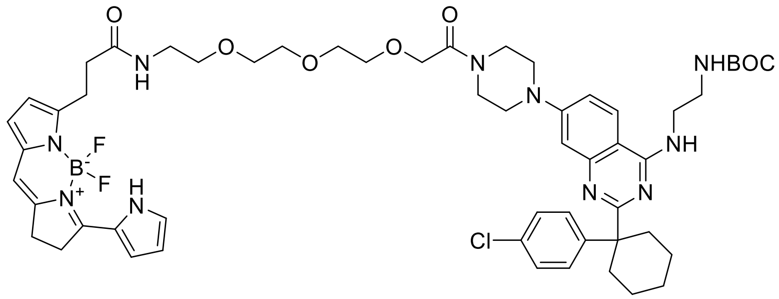

N-Methylmorpholine (4.1 µL, 37 µmol, 3.0 eq.) was added to a solution of 3-(5,5-difluoro-3-(1H-pyrrol-2-yl)-2,5-dihydro-1H-4λ^4,5λ^4-dipyrrolo[1,2-c:2',1'-f][1,3,2]diazaborinin-7-yl)propanoic acid (0.004 g, 12 µmol, 1.0 eq.) and HATU (0.007 g, 18 µmol, 1.5 eq.) in DMF (0.25 mL) The resulting solution was stirred at room temperature for 15 minutes. N-Methylmorpholine (9.5 µL, 86 µmol, 7 eq.) was added to a second solution of tert-butyl N-[2-({7-[4-(2-{2-[2-(2-aminoethoxy)ethoxy]ethoxy}acetyl)piperazin-1-yl]-2-[1-(4-chlorophenyl)cyclohexyl]quinazolin-4-yl}amino)ethyl]carbamate (**29**) (0.012 g, 12 µmol, 1.0 eq.) in DMF (0.25 mL). The two solutions were combined and stirred at room temperature for 1 h. The reaction mixture was concentrated to dryness and the residue was purified by preparative HPLC to afford N-(2-(2-(2-(2-(4-(4-((2-(((λ^1-methyl)(λ^1-oxidaneyl)boraneyl)amino)ethyl)amino)-2-(1-(4-chlorophenyl)cyclohexyl)quinazolin-7-yl)piperazin-1-yl)-2-oxoethoxy)ethoxy)ethoxy)ethyl)-3-(5,5-difluoro-3-(1H-pyrrol-2-yl)-2,5-dihydro-1H-4λ^4,5λ^4-dipyrrolo[1,2-c:2',1'-f][1,3,2]diazaborinin-7-yl)propanamide (**30**) as a blue solid.

Yield: 0.0093 g (61 %); LRMS (ESI+) *m/z*: 1065.2 $[M + H]^+$.

N-(2-(2-(2-(2-(4-(4-((2-aminoethyl)amino)-2-(1-(4-chlorophenyl)cyclohexyl)quinazolin-7-yl)piperazin-1-yl)-2-oxoethoxy)ethoxy)ethoxy)ethyl)-3-(5,5-difluoro-3-(1H-pyrrol-2-yl)-2,5-dihydro-1H-4λ^4,5λ^4-dipyrrolo[1,2-c:2',1'-f][1,3,2]diazaborinin-7-yl)propanamide (**31**)

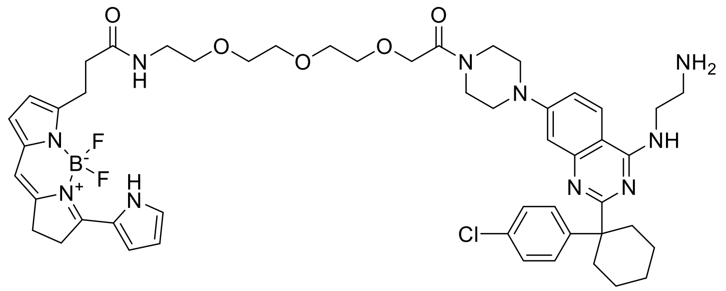

N-(2-(2-(2-(2-(4-(4-((2-(((λ^1-methyl)(λ^1-oxidaneyl)boraneyl)amino)ethyl)amino)-2-(1-(4-chlorophenyl)cyclohexyl)quinazolin-7-yl)piperazin-1-yl)-2-oxoethoxy)ethoxy)ethoxy)ethyl)-3-(5,5-difluoro-3-(1H-pyrrol-2-yl)-2,5-dihydro-1H-4λ^4,5λ^4-dipyrrolo[1,2-c:2',1'-f][1,3,2]diazaborinin-7-yl)propanamide (**30**) (0.0093 g, 0.01 mmol, 1.0 eq.) was dissolved in formic acid (1.0 mL) and the solution was stirred at 40 °C for 1 h. The volatiles were removed under reduced pressure and the residue was purified by preparative HPLC to afford chromatography to afford N-(2-(2-(2-(2-(4-(4-((2-aminoethyl)amino)-2-(1-(4-chlorophenyl)cyclohexyl)quinazolin-7-yl)piperazin-1-yl)-2-oxoethoxy)ethoxy)ethoxy)ethyl)-3-(5,5-difluoro-3-(1H-pyrrol-2-yl)-2,5-dihydro-1H-4λ^4,5λ^4-dipyrrolo[1,2-c:2',1'-f][1,3,2]diazaborinin-7-yl)propanamide (**31**) as a dark blue solid.

Yield: 0.0016 g (19%); LRMS (ESI+) *m/z*: 965.1 $[M + H]^+$.
