# Supplementary material for: OICR-41103 as a chemical probe for the DCAF1 WD40 domain
Source: Commun Biol. 2025 Jul 19;8:1076. doi: 10.1038/s42003-025-08491-0 (PMC12276300; doi:10.1038/s42003-025-08491-0)
Supplement: Supplementary file 2 — Description of Additional Supplementary Files [file 42003_2025_8491_MOESM2_ESM.pdf]

Description of Additional Supplementary Files

File name: Supplementary Data
Description: all source data underlying the graphs and charts presented in the main figures
