# Supplementary material for: OICR-41103 as a chemical probe for the DCAF1 WD40 domain
Source: Commun Biol. 2025 Jul 19;8:1076. doi: 10.1038/s42003-025-08491-0 (PMC12276300; doi:10.1038/s42003-025-08491-0)
Supplement: Supplementary file 4 — Reporting Summary [file 42003_2025_8491_MOESM4_ESM.pdf]

nature portfolio

Corresponding author(s): Rima Al-awar & Levon Halabelian

Last updated by author(s): Jun 17, 2025

Reporting Summary

Nature Portfolio wishes to improve the reproducibility of the work that we publish. This form provides structure for consistency and transparency in reporting. For further information on Nature Portfolio policies, see our Editorial Policies and the Editorial Policy Checklist.

Statistics

For all statistical analyses, confirm that the following items are present in the figure legend, table legend, main text, or Methods section.

n/a	Confirmed	
☐	☒	The exact sample size (n) for each experimental group/condition, given as a discrete number and unit of measurement
☒	☐	A statement on whether measurements were taken from distinct samples or whether the same sample was measured repeatedly
☒	☐	The statistical test(s) used AND whether they are one- or two-sided<br>*Only common tests should be described solely by name; describe more complex techniques in the Methods section.*
☒	☐	A description of all covariates tested
☒	☐	A description of any assumptions or corrections, such as tests of normality and adjustment for multiple comparisons
☐	☒	A full description of the statistical parameters including central tendency (e.g. means) or other basic estimates (e.g. regression coefficient) AND variation (e.g. standard deviation) or associated estimates of uncertainty (e.g. confidence intervals)
☒	☐	For null hypothesis testing, the test statistic (e.g. F, t, r) with confidence intervals, effect sizes, degrees of freedom and P value noted<br>*Give P values as exact values whenever suitable.*
☒	☐	For Bayesian analysis, information on the choice of priors and Markov chain Monte Carlo settings
☒	☐	For hierarchical and complex designs, identification of the appropriate level for tests and full reporting of outcomes
☒	☐	Estimates of effect sizes (e.g. Cohen's d, Pearson's r), indicating how they were calculated

Our web collection on statistics for biologists contains articles on many of the points above.

Software and code

Policy information about availability of computer code

Data collection	Diffraction data were collected on the CMCF-BM beamline at the Canadian Light Source.
Data analysis	Diffraction data were processed with HKL3000 and the structure solved by molecular replacement in Phaser using the DCAF1-8268 crystal structure (PDB ID: 8F8E) as the starting model. The model was refined by alternating cycles of manual rebuilding in Coot and refinement with Refmac within the CCP4 crystallographic suite.

For manuscripts utilizing custom algorithms or software that are central to the research but not yet described in published literature, software must be made available to editors and reviewers. We strongly encourage code deposition in a community repository (e.g. GitHub). See the Nature Portfolio guidelines for submitting code & software for further information.

Data

Policy information about availability of data

All manuscripts must include a data availability statement. This statement should provide the following information, where applicable:

- Accession codes, unique identifiers, or web links for publicly available datasets
- A description of any restrictions on data availability
- For clinical datasets or third party data, please ensure that the statement adheres to our policy

Atomic coordinates and structure factors for DCAF1-OICR-41103 structure have been deposited in the Protein Data bank under the accession code: 9D4E

Research involving human participants, their data, or biological material

Policy information about studies with human participants or human data. See also policy information about sex, gender (identity/presentation), and sexual orientation and race, ethnicity and racism.

Reporting on sex and gender	N/A
Reporting on race, ethnicity, or other socially relevant groupings	N/A
Population characteristics	N/A
Recruitment	N/A
Ethics oversight	N/A

Note that full information on the approval of the study protocol must also be provided in the manuscript.

Field-specific reporting

Please select the one below that is the best fit for your research. If you are not sure, read the appropriate sections before making your selection.

☒ Life sciences ☐ Behavioural & social sciences ☐ Ecological, evolutionary & environmental sciences

For a reference copy of the document with all sections, see nature.com/documents/nr-reporting-summary-flat.pdf

Life sciences study design

All studies must disclose on these points even when the disclosure is negative.

Sample size	Sample size was based on standard practice for in vitro assays. Experiments were performed with three biological replicates (n=3).Sample sizes are described in Methods and figure legends
Data exclusions	Resolution cutoff was applied to the DCAF1-OICR-41103 X-ray diffraction data using both CC1/2 and (1/delta) pre-established criteria.
Replication	All experiments were independently repeated three times with consistent results.
Randomization	Samples were not randomized, as randomization is not applicable to this type of in vitro cell-based assay.
Blinding	Subsets of X-ray diffraction amplitudes were withheld for calculation of Rfree values. For the remaining experiments, blinding was not performed as data collection was automated and sample identity was known.

Reporting for specific materials, systems and methods

We require information from authors about some types of materials, experimental systems and methods used in many studies. Here, indicate whether each material, system or method listed is relevant to your study. If you are not sure if a list item applies to your research, read the appropriate section before selecting a response.

Materials & experimental systems

n/a	Involved in the study	
☐	☒	Antibodies
☐	☒	Eukaryotic cell lines
☒	☐	Palaeontology and archaeology
☒	☐	Animals and other organisms
☒	☐	Clinical data
☒	☐	Dual use research of concern
☒	☐	Plants

Methods

n/a	Involved in the study	
☒	☐	ChIP-seq
☒	☐	Flow cytometry
☒	☐	MRI-based neuroimaging

Antibodies

Antibodies used	1) VPRBP (D5K5V) Rabbit mAb (Cell Signaling Technology #14966)<br>2) GAPDH (14C10) Rabbit mAb (Cell Signaling Technology #2118)
Validation	1) VPRBP (D5K5V) Rabbit mAb (Cell Signaling Technology #14966)<br>a) Validated by Cell signaling Technology using Western blot. Species Reactivity: Human, Mouse, Rat, Monkey<br>b) In house validation using western blot upon shRNA knockdown of the protein or over-expression of a Flag tagged DCAF1.<br>2) GAPDH (14C10) Rabbit mAb (Cell Signaling Technology #2118)<br>a) Validated by Cell signaling Technology using Western blot. Species Reactivity: Human, Mouse, Rat, Bovine, Pig

Eukaryotic cell lines

Policy information about cell lines and Sex and Gender in Research

Cell line source(s)	1) ALL parental cell lines purchased from ATCC<br>2) H460 cells expressing HiBiT-tagged WD40 DCAF1--> Engineered in-house using the Parental H460 (ATCC)<br>2) 293EMT cell line--> Engineered in-house<br>3) shCTR2, shPLK1, shDCAF1-2 and shDCAF1-7 in H1703, H1915 and H2170--> Engineered in-house using parental cell lines (ATCC)<br>4)HEK293T-kind gift from Sam Benchimol, York University, ATCC®CRL-3216™<br>5)MCF7-(ATCC® HTB-22™) purchased from ATCC<br>6)HCT116-(ATCC®CCL-247™) purchased from ATCC<br>7)U2Os-(ATCC®HTB-96™) urchased from ATCC
Authentication	Cell lines purchased from ATCC are routinely verified using short tandem repeat (STR).
Mycoplasma contamination	OICR lab policies:<br>1) Cell lines purchased from ATCC are not tested for Mycoplasma.<br>2) Cell lines provided by collaborates are routinely tested for Mycoplasma. No Mycoplasma contamination was found
Commonly misidentified lines (See ICLAC register)	N/A

Plants

Seed stocks	N/A
Novel plant genotypes	N/A
Authentication	N/A
